# Genome-wide meta-analysis identified novel variant associated with hallux valgus in Caucasians

**DOI:** 10.1186/s13047-020-0379-1

**Published:** 2020-03-04

**Authors:** Liubov Arbeeva, Michelle Yau, Braxton D. Mitchell, Rebecca D. Jackson, Kathleen Ryan, Yvonne M. Golightly, Marian T. Hannan, Amanda Nelson, Joanne M. Jordan, Marc C. Hochberg

**Affiliations:** 1 Thurston Arthritis Research Center, University of North Carolina, Thurston Arthritis Research Center, 3300 Thurston Building, Campus Box #7280, Chapel Hill, NC 27599-7280 USA; 2 Hebrew SeniorLife Marcus Institute for Aging Research and Harvard Medical School, Boston, MA USA; 3 University of Maryland School of Medicine, Baltimore, MD USA; 4 Geriatrics Research and Education Clinical Center, Baltimore Veterans Administration Medical Center, Baltimore, MD USA; 5 Department of Internal Medicine, Division of Endocrinology, Diabetes and Metabolism, The Ohio State University, Columbus, OH USA; 6 Department of Epidemiology, Gillings School of Global Public Health, University of North Carolina, Chapel Hill, NC USA; 7 Injury Prevention Research Center, University of North Carolina, Chapel Hill, North Carolina, USA; 8 Division of Physical Therapy, University of North Carolina, Chapel Hill, North Carolina, USA; 9 Department of Medicine, University of North Carolina, Chapel Hill, NC USA; 10 Department of Orthopedics, University of North Carolina, Chapel Hill, NC USA

**Keywords:** Genome-wide, Muscle disease, Genetic epidemiology, Rheumatology

## Abstract

**Background:**

Hallux valgus, one of the most common structural foot deformities, is highly heritable. However, previous efforts to elucidate the genetic underpinnings of hallux valgus through a genome-wide association study (GWAS) conducted in 4409 Caucasians did not identify genome-wide significant associations with hallux valgus in both gender-specific and sex-combined GWAS meta-analyses. In this analysis, we add newly available data and more densely imputed genotypes to identify novel genetic variants associated with hallux valgus.

**Methods:**

A total of 5925 individuals of European Ancestry were categorized into two groups: ‘hallux valgus present’ (*n* = 2314) or ‘no deformity’ (*n* = 3611) as determined by trained examiners or using the Manchester grading scale. Genotyping was performed using commercially available arrays followed by imputation to the Haplotype Reference Consortium (HRC) reference panel version 1.1. We conducted both sex-specific and sex-combined association analyses using logistic regression and generalized estimating equations as appropriate in each cohort. Results were then combined in a fixed-effects inverse-variance meta-analyses. Functional Mapping and Annotation web-based platform (FUMA) was used for positional mapping, gene and gene-set analyses.

**Results:**

We identified a novel locus in the intronic region of *CLCA2* on chromosome 1, rs55807512 (OR = 0.48, *p* = 2.96E-09), an expression quantitative trait locus for *COL24A1*, a member of the collagen gene family.

**Conclusion:**

In this report of the largest GWAS of hallux valgus to date, we identified a novel genome-wide significant locus for hallux valgus. Additional replication and functional follow-up will be needed to determine the functional role of this locus in hallux valgus biology.

## Introduction

Hallux valgus, one of the most common structural foot deformities, is characterized by abduction of the great toe (hallux) with respect to the first metatarsal joint [1]. Hallux valgus is associated with pain, functional limitation, increased risk for falls, and diminished quality of life [2–4]. The condition is multifactorial in origin and the etiology is not completely understood. Hallux valgus is associated with female sex, older age, lower body mass index (BMI), and certain footwear types [1, 5–7]. Structural factors, such as metatarsal length and head shape, first ray hypermobility, and hind-foot pronation, are also considered to be important in hallux valgus development [6]. Hallux valgus is heritable, with estimates ranging from 0.29 to 0.89, suggesting that genetics may influence the development of this deformity [8, 9]. Identifying genetic variants associated with hallux valgus using an agnostic genome-wide approach may provide insights into the development of hallux valgus and lead to new treatment strategies.

The first and only genome-wide association study (GWAS) of hallux valgus was conducted as a meta-analysis in 4409 Caucasians based on a combined analysis of the Framingham Heart Study (FHS), the Genetics of Generalized Osteoarthritis (GOGO) Study, and the Johnston County Osteoarthritis Project (JoCoOA) [10]. This study did not find genome-wide significant associations with hallux valgus in either gender-specific or sex-combined GWAS meta-analyses. In this report, we expand the prior genome-wide association analysis by including association results from the Osteoarthritis Initiative (OAI), in which hallux valgus has also been measured and genome-wide genotyping is available.

The objective of the present paper is to identify novel genetic variants associated with hallux valgus in this expanded sample and with deeper genotype imputation performed (i.e., from 1000 Genomes to the Haplotype Reference Consortium (HRC) reference panel). With the addition of the OAI, the GWA sample size increased to 5925 Caucasian participants, representing a 34% increase in size from the prior GWA sample of 4409 subjects.

## Methods

### Study cohorts and assessment of hallux valgus

The meta-analysis included participants of European ancestry from four cohort studies: the Framingham Heart Study (FHS), the Genetics of Generalized Osteoarthritis (GOGO) Study, the Johnston County Osteoarthritis Project (JoCoOA), and the Osteoarthritis Initiative (OAI).

### Framingham Heart Study

FHS is a community-based prospective study that began in 1948 with 5209 Framingham residents primarily white men and women of European-ancestry [11]. In 1972, 5124 offspring of the Original Cohort and their spouses were enrolled into the Offspring Cohort [12]. Our sample is limited to 2264 participants from Original and Offspring cohorts who were successfully genotyped and enrolled into Framingham Foot Study, an ancillary study of the FHS that was designed to examine the contribution of foot disorders to functional limitations [13]. Foot disorders, including hallux valgus, were assessed using a validated Foot Assessment Clinical Tool that captures the main features of common foot disorders by trained clinical examiners^14 15^. The validity of this tool was evaluated in a sample of elderly residents by comparing podiatry clinic findings to the results from the study examiners. The inter-observer and intra-observer reliability for hallux valgus were excellent [14, 15]. Hallux valgus was considered to be present if the angle of the hallux towards the lesser toes on either foot was observed to be greater than 15 degrees while weight-bearing, in either foot.

### Genetics of Generalized Osteoarthritis

GOGO is a multisite collaboration involving seven sites in the United States and United Kingdom (UK). The purpose of study was to identify chromosomal regions associated with increased predisposition to generalized osteoarthritis (OA). The GOGO cohort is a sample of 2728 participants with and without hand OA from 1145 qualified families (at least two siblings with polyarticular OA). The study design has been previously reported [16]. A total of 1231 participants were successfully genotyped and completed clinical examination of the feet, including hallux valgus assessment (same method as JoCoOA, described in next section).

### Johnston County Osteoarthritis Project

JoCoOA is an ongoing, community-based, prospective study of the occurrence of OA in Caucasian and African American residents in a rural North Carolina county [17, 18]. A total of 3187 participants were recruited at the 1991–97 baseline with an additional 1015 participants recruited into an enrichment cohort during 2003–2004. During the 2006–10 follow-up visit, 1695 participants completed clinical examination of the foot, including hallux valgus, performed by a trained clinical examiner. Of these, 919 successfully genotyped Caucasian participants were included into this study.

In GOGO and JoCoOA, structural deformities and conditions of the foot were classified as present and absent. Hallux valgus was assessed for each foot using a laminated foot diagram with two lines intersecting at 15°. Participants stood on the diagram with the medial edge of one foot against one line and their first metatarsophalangeal joint at the apex of the two lines. Hallux valgus was recorded as present if the angle of the great toe was greater than 15 degrees in either foot [5, 19, 20]. In JoCoOA, the inter-rater reliability for the hallux valgus measure was excellent for the left foot (kappa 0.84, 95% CI 0.73, 0.96) and good for the right foot (kappa 0.71, 95% CI 0.57, 0.92) [5].

### Osteoarthritis Initiative

The OAI is a multi-center, longitudinal, prospective study, designed to identify risk factors for the development and progression of symptomatic knee OA [21]. Participants were recruited at clinical centers in Columbus, Ohio; Baltimore, Maryland; Pittsburgh, Pennsylvania; and Providence, Rhode Island who either were at risk for or had symptomatic radiographic knee OA. A total of 4796 received a baseline evaluation between 2004 and 2006 and were invited to annual follow-up visits for up to 8 years. Hallux valgus was assessed at the 96 month follow-up visit. First, participants were asked if they had ever had a bunionectomy on one or both feet (yes/no). Next, the presence and severity of hallux valgus was determined using the Manchester grading scale, which is recommended as a simple, non-invasive screening tool for clinical and research purposes [22, 23]. A trained and certified examiner compared the participants’ feet to photographs showing four grades and assigned a grade of hallux valgus deformity (grades 1–4: no deformity, mild deformity, moderate deformity, severe deformity) for each participant’s right and left foot separately. Because the severity of hallux valgus was not measured in FHS, GOGO and JoCoOA, the Manchester grades were collapsed into dichotomous categories to indicate presence and absence of hallux valgus based on recommendations from Menz et al. [23, 24]. In the publication by Menz et al., re-test reliability and agreement between dichotomous scores obtained by the examiners and the participants were similar to the levels reported for four severity categories [24]. For our main analyses in OAI, hallux valgus was considered present if participants reported a prior bunionectomy or if one or both feet had a Manchester grade of 3 or 4 (moderate or severe deformity). Hallux valgus was considered absent if participants reported no prior bunionectomy and had a Manchester grade of 1 (no deformity) in both feet. In a sensitivity analysis, OAI participants with Manchester grade of 2 (mild deformity) were added to the ‘no deformity’ group. Therefore, OAI provided two sets of GWAS results: (1) for the main analyses with the original definition of hallux valgus (*N* = 1511), and (2) for a sensitivity analysis allowing mild deformity to be included in the ‘no deformity’ group (*N* = 2120).

### Genotyping, quality control (QC) and imputation

Details on genotyping and calling for each cohort were described elsewhere [10, 25]. In brief, genotyping was performed using commercially available arrays. To increase the number of tested SNPs and the overlap of variants available for analysis between different arrays, all Caucasian cohorts imputed genotypes to the most current HRC v1.1 reference panel [26] on the Michigan Imputation Server [27]. Additional details on genotyping and pre-imputation quality control in each study are listed in Supplementary Table 1.

### Genome-wide association analyses

Following imputation, each study conducted GWAS under an additive genetic model, for the total sample and for women and men separately, to test the effect of imputed allelic dose on presence vs. absence of hallux valgus. For JoCoOA and OAI, the logistic regression model in PLINK v1.90 software was applied [28]. To account for within-family correlations in FHS and GOGO, the generalized estimating equations (GEE) model with the kinship matrix implemented in the R package GEE-pack [29] was used. In sex-specific GWAS, the models were adjusted for age at the time of foot examination, BMI, recruitment site (for OAI and GOGO), and population structure using the principal components. In analyses combining results for men and women, the models were additionally adjusted for sex.

Prior to meta-analysis, we performed post-GWAS harmonization and QC of GWAS results from each cohort to track possible errors in the study-specific analyses. We used the standard protocol accompanied by EasyQC R package [30]. Specifically, we removed single nucleotide polymorphisms (SNPs) with low minor allele frequencies (MAF) (< 0.01), low imputation quality (< 0.6), low minor allele count (<=10), large absolute values of beta coefficients and standard errors (> = 10), low call rate (< 0.95), and deviations from Hardy-Weinberg equilibrium (*p* < 10^− 6^).

The association results were combined using an inverse variance weighted fixed-effects meta-analysis in METAL software [31], with correction for genomic control. This method weights effect size estimates using the inverse of the corresponding standard errors. As noted previously, in each of the main analyses conducted in men, women, and both sexes combined, we excluded OAI participants categorized with mild hallux valgus deformity (grade 2), but included these participants in a sensitivity analysis. Heterogeneity was assessed using the I^2^ metric from the complete study-level meta-analysis. Between-study heterogeneity was tested using the Cochran Q statistic and considered significant at *p* = 0.1. A genome-wide significance threshold was set at the level of *p* = 5.0 × 10^− 8^. The Manhattan plots were generated in R. LocusZoom (http://locuszoom.org/) was used to provide regional visualization of results. We performed approximate conditional analysis (e.g., association analysis conditioning on the primary associated SNPs) using Genome-wide Complex Trait Analysis tool (GCTA v1.24) [32] to identify independent signals in suggestive loci. We defined a locus as a chromosomal region at which adjacent pairs of associated SNPs are less than 1 Mb distant. The collinearity threshold was set at *r*^*2*^ = 0.9, so that highly correlated SNPs are not selected in model.

Finally, we attempted to replicate findings from the discovery analysis in the UK Biobank by looking up findings in a GWAS of hallux valgus that has been made publicly available by the Neale lab at the Broad Institute http://www.nealelab.is/blog/2017/9/11/details-and-considerations-of-the-uk-biobank-gwas. The Neale lab conducted GWAS for 2419 phenotypes in the UK Biobank, which included hallux valgus defined by self-report. For the purpose of simplifying the process of association testing, the linear model with adjustment for sex and 10 principal components was fitted for all outcomes. Fitting a linear model to a binary outcome such a hallux valgus can introduce biases in coefficients and *p*-values due to violation of asymptotic assumptions of a linear model, especially for SNPs with low MAF in studies with relatively small sample sizes. Therefore, we followed the authors’ recommendations to remove SNPs below an allele frequency threshold defined as 25 divided by the smallest case group or 25/2314 = 0.01. We considered SNPs to replicate if they reached a nominal significance of *p* = 0.05 in the Neale lab data.

### Functional annotation of SNPs and gene mapping

We performed functional annotation of GWAS results using Functional Mapping and Annotation of GWAS platform (FUMA) [33]. FUMA matches variants by chromosome, base-pair position, reference and alternate alleles to multiple publicly available databases to predict functional consequences for these SNPs, retrieve information on previously known SNP trait-association from the GWAS catalog, accommodate gene mapping, and to provide gene-based, pathway and tissue enrichment results. We also used PhenoScanner v2 to evaluate whether any of our associated or near-associated SNPs have been previously associated with musculoskeletal traits.

We assigned functional annotations to significant SNPs (*p* ≤ 5.0 × 10^− 7^ for analyses in the total sample; *p* ≤ 5.0 × 10^− 6^ for sex-specific analyses) and SNPs in linkage disequilibrium (LD) with significant SNPs (*r*^*2*^ > 0.6) using the SNP2GENE FUMA function, which incorporates tools from ANNOVAR, CADD, and RegulomeDB. ANNOVAR annotates functional effects of variants with respect to genes [34]. CADD predicts deleteriousness of the effect of a SNP on protein function. Higher CADD score refers to the more deleterious variants [35]. RegulomeDB scores variants based on information from expression quantitative trait loci (eQTLs) and chromatin marks. The score ranges from 1a to 7, where lower scores indicate increasing evidence that a variant is located in a functional region [36]. All LD information was calculated from the 1000 Genomes Phase 3 release reference panel.

SNPs were mapped to genes based on positional, eQTL, and 3D chromatin interaction mapping. Positional mapping was performed by selecting exonic and splicing SNPs with CADD score > =12.37. This threshold is recommended to restrict the mapping to deleterious coding SNPs [35]. We used eQTLs with false discovery rate (FDR) < 0.05 in 7 tissue types (adipose subcutaneous, whole blood, artery tibial, muscle skeletal, nerve tibial, cells transformed fibroblasts, skin sun exposed lower leg) from the Genotype Tissue Expression database (GTEx v7) [37, 38] and from additional data repositories (eQTLGen, xQTLServer [39], and MuTHER [40]). For chromatin interactions, Hi-C data in two tissues (psoas and mesenchymal stem cell) from GSE87112 were used; interactions were filtered by FDR < 10^− 6^. The MHC region was excluded from the analysis. We used MAGMA v1.07, which is integrated in FUMA to generate *p*-values quantifying the degree of association of genes and gene sets with hallux valgus [41]. GWAS summary statistics were aggregated to the level of whole genes to test the joint association of all markers in the gene with hallux valgus. This aggregation reduces the number of tests that are performed and identifies effects consisting of multiple weaker associations. Individual genes were then aggregated into groups of genes sharing certain biological, functional or other characteristics. We applied a default competitive model to test whether genes in a gene set are more strongly associated with hallux valgus than other gene sets. Tissue enrichment analyses were conducted in FUMA using two types of tissues from GTEx: 30 general tissue types from multiple organs and 53 specific tissue types within these organs.

## Results

### Characteristics of participants and prevalence of hallux valgus in the discovery sample

Sample characteristics of the 5925 Caucasian participants (2314 categorized as ‘hallux valgus present’ and 3611 categorized as ‘no deformity’) who were included in the main analysis are summarized in Table 1. The mean age of participants is 66, ranging from 39 to 100 years. JoCoOA participants were older and had higher BMI compared to the three other cohorts. Within cohorts, cases were more likely to be female and older compared to controls. Hallux valgus was less prevalent and the proportion of men was higher in FHS compared to the other cohorts. In the total sample, cases were slightly older (mean age 67.8 vs 64.5), and proportion of females was higher among cases than among those without deformity. There were no case-control differences with respect to BMI.

**Table 1:** Baseline characteristics of the participants of European Ancestry from the Framingham Heart Study (FHS), the Genetics of Generalized Osteoarthritis (GOGO) Study, the Johnston County Osteoarthritis Project (JoCoOA), and the Osteoarthritis Initiative (OAI)

	FHS		GOGO		JoCoOA		OAI	
	Cases	Controls	Cases	Controls	Cases	Controls	Cases	Controls
Total, N	709	1555	512	719	445	474	648	863
% cases	31.3%		41.6%		48.4%		42.9%	
Women, N	516	752	437	558	299	268	453	367
% cases	40.7%		43.9%		52.7%		55.2%	
Men, N	193	803	75	161	146	206	195	496
% cases	19.4%		31.8%		41.5%		28.2%	
Age Mean (SD)	70.1 (11.5)	66.6 (10.6)	67.2 (8.9)	64.2 (8.7)	71.3 (9.1)	67.1 (8.3)	62.5 (8.6)	60.0 (9.1)
BMI Mean (SD)	27.5 (5.0)	28.8 (5.5)	29.0 (6.2)	28.7 (6.0)	30.5 (6.5)	30.7 (6.2)	27.8 (4.5)	28.6 (4.6)

Values are Mean (SD) unless otherwise specified

*BMI* Body mass index

### GWAS meta-analysis for total sample (Caucasians)

**Fig. 1:**
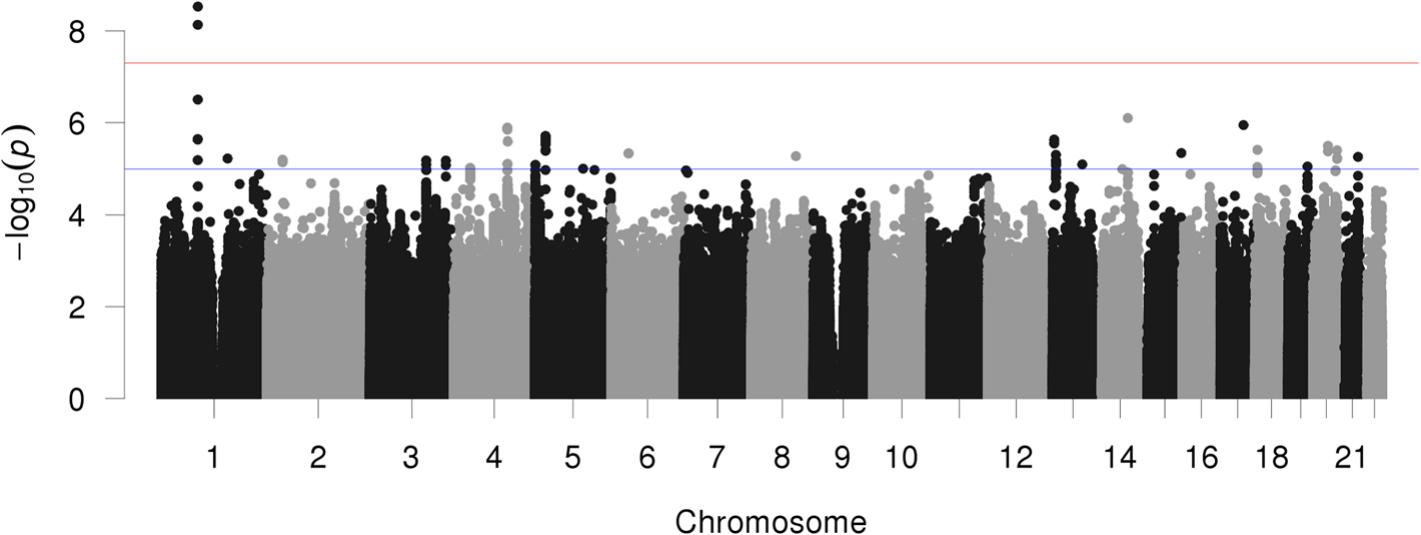
Manhattan plot represents the *p*-values of the entire GWAS on genomic scale. The *p*-values are ordered by chromosome and position on the corresponding chromosome (x-axis). The value on the y-axis is the (–log10) of the *p*-value and is equivalent to the number of zeros after the decimal point plus one. The graph looks like a Manhattan skyline because of local correlation of SNPs. The red line shows the threshold for genome-wide significance

After removal of SNPs that failed to meet the post-GWAS QC criteria, the number of variants included in meta-analysis was 7,410,639 in FHS, 7,695,976 in JoCoOA, 7,646,026 in GOGO, and 7,729,175 in OAI. The results of gender-combined meta-analysis are summarized in the Manhattan plot (Fig. 1).

**Fig. 2:**
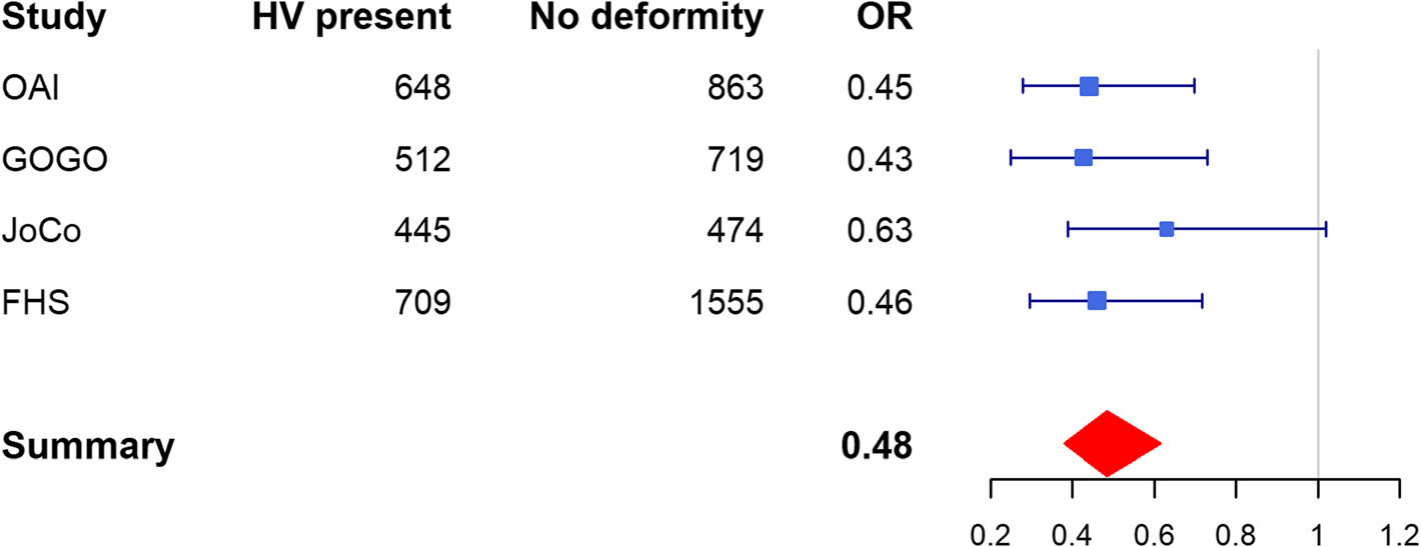
Forest plot of rs55807512 (*CLCA2*, chr1) association with hallux valgus in the meta-analysis

**Fig. 3:**
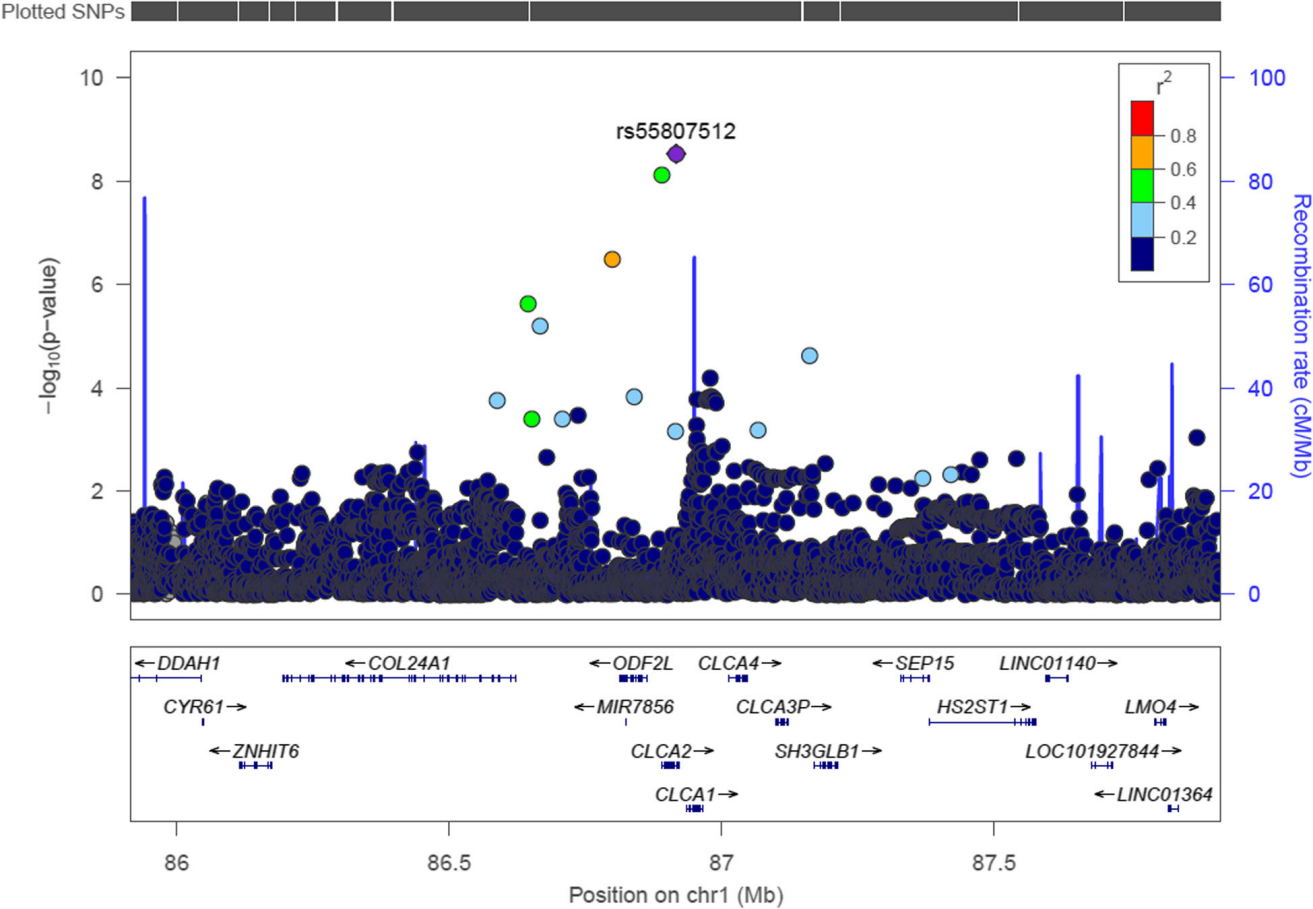
Regional association plot of *CLCA2* locus

A genome-wide significant association was found for two variants located in an intronic region of chromosome 1 within the *CLCA2* gene: rs55807512 (MAF = 4%, OR = 0.48, *p* = 2.96E-09) and rs12124247 (MAF = 3%, OR = 2.19, *p* = 7.38E-09). Effect direction was consistent across all four data sets (Fig. 2); these SNPs are in a weak LD (*r* [2] = 0.46). In conditional analysis, the effect of rs12124247 was attenuated and did not remain significant when conditioned on rs55807512 and vice versa indicating that both SNPs tag the same signal. No other SNPs were in a high LD (*r*^*2*^ > =0.8) with the top variant as shown in the regional plot (Fig. 3). Thirty additional SNPs were associated with hallux valgus at *p* < 5.0 × 10^− 6^ (Table 2, Supplementary Table 2). In the sensitivity analysis with an additional 609 OAI participants in the control group, the two top-hits remained significant (Table 2, Supplementary Table 3, Supplementary Figure 1) and no additional loci were identified.

**Table 2:** Top 10 SNPs from meta-analysis, adjusted for sex, BMI, age, study site, and principal components (total sample, *N* = 5925)

SNP rsID	Chr:Position	Nearest Gene	EA/OA	EAF	OR (95% CI)	*P*-value	Direction	I2	Het. P-value	*P*-value sens.
rs55807512	1:86916200	**CLCA2**	t/c	0.96	0.48 (0.38, 0.61)	2.96E-09	–	0	0.67	4.75E-10
rs12124247	1:86890244	**CLCA2**	a/g	0.03	2.19 (1.68, 2.86)	7.38E-09	++++	0	0.68	2.39E-09
rs146496015	1:86799387	ODF2L	t/g	0.06	1.61 (1.34, 1.94)	3.14E-07	++++	1.6	0.38	5.25E-07
rs12888772	14:81578926	**RP11-114 N19.3**	a/t	0.18	1.30 (1.17, 1.44)	7.92E-07	++++	0	0.96	1.55E-07
rs117155606	17:54325272	**ANKFN1**	t/c	0.01	5.53 (2.78, 11.02)	1.13E-06	? +???	0	1	1.13E-06
rs11728629	4:126712045	RP11-404I7.1	a/t	0.24	1.26 (1.15, 1.38)	1.28E-06	++++	0	0.8	1.32E-05
rs4314298	4:126710287	RP11-404I7.1	a/g	0.76	0.80 (0.73, 0.87)	1.42E-06	–	0	0.82	1.52E-05
rs116783165	5:25669440	CTD-2533 K21.1	c/g	0.03	1.72 (1.37, 2.15)	1.95E-06	++++	58.6	0.06	5.56E-06
rs116336450	5:25663890	CTD-2533 K21.1	t/c	0.97	0.58 (0.47, 0.73)	2.13E-06	–	60.5	0.06	5.87E-06
rs79433053	5:25668523	CTD-2533 K21.1	t/c	0.03	1.71 (1.37, 2.14)	2.17E-06	++++	60.3	0.06	5.98E-06

*Chr* Chromosome, *EA* Effect allele, *OA* Other allele, *OR* Odds ratio, *CI* Confidence interval, *Het* Heterogeneity, *sens* Sensitivity

Intron SNPs are indicated in bold

### Sex-specific GWAS meta-analysis

The association signals diminished in sex-specific analyses (Supplementary Tables 4 and 5). In both men and women, the top-hits from the sex-combined analysis did not reach the genome-wide significance level at 5.0 × 10^− 8^. In men, we found only a single SNP passing the post-GWAS QC in the three cohorts to be significantly associated with hallux valgus: rs141161671 (MAF = 1%, OR = 6.50, *p* = 3.22E-08), located in the intronic region of chromosome 2 within *AC007682.1* gene. The remaining SNPs with *p* < 5.0 × 10^− 6^ are listed in Supplementary Table 4. In women, we did not find any SNPs to be significantly associated with hallux valgus. However, rs55807512, the lead variant in the total sample analysis, was associated with hallux valgus with a *p*-value of 1.73E-06 (MAF = 4%, OR = 0.47). The remaining SNPs with *p* < 5.0 × 10^− 6^ are listed in Supplementary Table 5.

### Replication

In the UK Biobank data (according to the summary statistics provided by the Neale Lab), neither rs55807512 nor rs12124247 were associated with hallux valgus. Several SNPs with *p* < 5.0 × 10^− 6^ in this meta-analysis showed nominal evidence (*p* < 0.05) for association with hallux valgus in the UK Biobank data (Supplementary Tables 2–3).

### Functional annotation

**Fig. 4:**
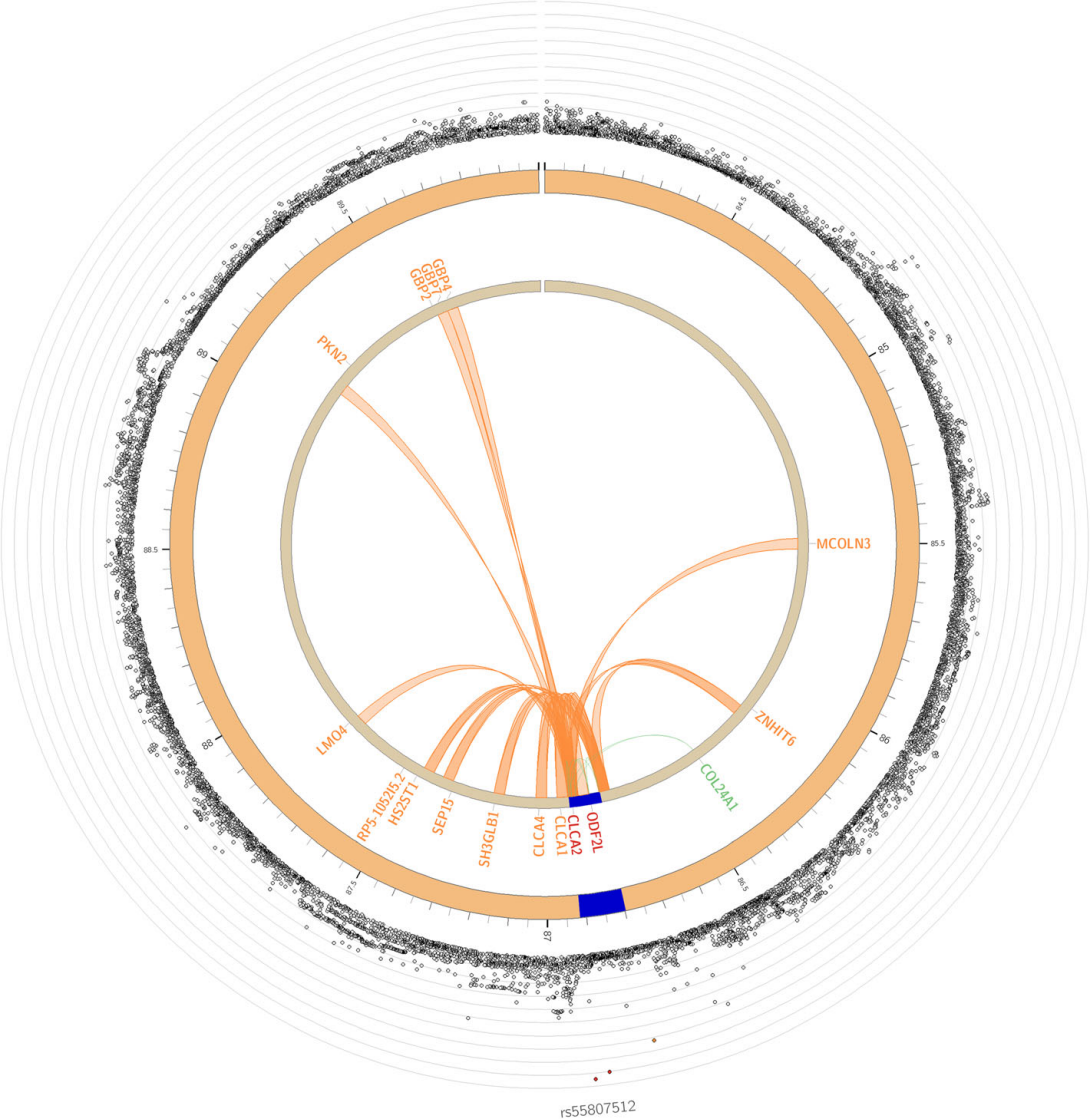
Circos plots of mapped gene on chromosome 1 locus. Genomic risk loci are highlighted in blue. Genes are mapped by 3-D chromatin interaction (orange) or eQTLs (green), or both (red)

FUMA identified one genomic risk locus on chromosome 1 tagged by the genome-wide significant lead SNP, rs55807512 (Fig. 4). No information on previously known SNP-trait associations was found for independent significant and tagged SNPs. Functional annotation of hallux valgus associated variants in *CLCA2* revealed that rs55807512 is among the top (< 10%) of deleterious mutations in the genome (CADD = 11.89). eQTL mapping showed that our top hits, rs55807512 and rs12124247, which are located in *CLCA2*, are eQTLs for *COL24A1* expression. 3D chromatin interactions revealed significant interactions between these genome-wide significant variants and 14 other genes on chromosome 1 (Fig. 4).

Gene and gene-set analyses did not show any significant associations. Of 18,722 protein coding genes tested, the most significantly associated gene was *RUFY1* (*p* = 4.8 × 10^− 6^, Supplementary Table 6). Of 10,673 gene sets tested, the most significantly associated gene sets were “furukawa_dusp6_targets_pci35_up”, “positive regulation of cartilage development”, and “positive regulation of chondrocyte differentiation” (*p* < 1 × 10^− 4^, Supplementary Table 7).

Tissue analyses on 30 general tissue types from multiple organs and 53 specific tissue types within these organs) did not reveal any statistically significant associations (Supplementary Figures 2–3).

## Discussion

In the expanded hallux valgus meta-analysis on individuals of European ancestry, we identified a novel locus for hallux valgus in *CLCA2*. This study presents an updated meta-analysis of the first genome-wide association screen performed in hallux valgus which did not identify genome-wide significant SNPs [10]. This can, in part, be attributed to relatively modest sample sizes. We increased the sample by including data from the OAI and imputed genotypes to the most current HRC reference panel.

The lead variant, rs55807512, located in an intronic region of chromosome 1 within *CLCA2* gene, had MAF around 4% and was not included in the first hallux valgus GWAS. Updating the imputation increased the number of low-frequency variants that were filtered out in previous analyses and can be studied reliably using the HRC reference panel. According to Entrez Gene database https://www.ncbi.nlm.nih.gov/gene, *CLCA2* encodes a member of the calcium-activated chloride channel regulator (CLCR) family of proteins that regulates transport of chloride across the plasma membrane. Although another member of CLCA family, *CLCA4*, has been reported to be associated with osteochondrosis in the horse [42], *CLCA2* has not been associated with bone formation or any musculoskeletal disorders. However, *COL24A1* may be the true gene of interest since our top hits were eQTLs for *COL24A1* expression. *COL24A1*, a member of the collagen gene family, is developmentally expressed in cornea and bone by osteoblasts and regulates osteoblast differentiation and mineralization through interactions with integrins, which leads to the activation of the TGF-β/ Smad signaling pathway [43–45]. Collagen type XXIV may be involved in structural differences between fibrillary collagens and affect fibril diameter [44, 46]. Abnormal collagen fibrils are associated with a wide spectrum of diseases of bone and cartilage, including hallux valgus [47, 48]. Uchiyama et al. [48] demonstrated that feet with hallux valgus have different structures of collagen fibers compared to normal feet. This may be in response to continuous stress to the medial collateral ligament, one of the important joint stabilizers, and lead to altered organization of collagen I and collagen III fibrils that could leave the first metatarsophalangeal joint unprotected during gait [48, 49].

An important paralog of *COL24A1* is *COL5A1*. Mutations in the *COL5A1* gene, encoding the alpha 1 of type V collagen, have been identified in patients with Ehlers-Danlos syndrome [50, 51] which has been linked to hallux valgus [52], Achilles tendinopathy [50], acquired injuries such as ACL tears [53], and with range of motion [50].

None of the top SNPs identified from the previous hallux valgus meta-analysis became more significant in our updated meta-analysis. Of the four SNPs that met *p* < 5E-6 in men, only r10224956 and rs4476613, reached nominal significance (*p* = 0.02 and *p* = 0.001, respectively) in our study. Of the six SNPs that met *p* < 5E-6 in women, only rs12214759 and rs2242411 reached suggestive significance (*p* = 6.70E-06 and *p* = 6.67E-05, respectively) in our study with the same direction of effect. Furthermore, none of the previously identified SNPs were associated with hallux valgus in the UK Biobank GWAS.

One of the difficulties in studying the genetics of hallux valgus is the lack of a standardized phenotype. The method of measuring hallux valgus in studies collecting such data is not always clearly described. Furthermore, hallux valgus prevalence in studies using self-report data may be under-reported or inaccurate due to a lack of a validated assessment tool for this condition and lack of standardization for terms used in questionnaires (e.g., “bunion” and “hallux valgus”) [1, 24].An important advantage to our study is the detailed assessment of hallux valgus based on objective criteria rather than self-report. Although the presence of hallux valgus was not measured using weight-bearing radiographs of the feet, the reference standard of angle measurement, the clinical measures we used have been previously validated and were conducted by trained examiners which should minimize potential sources of error. These tools have been reported as alternatives to radiographs due to lower cost and lack of radiographic exposure, particularly for large-scale cohort studies that include asymptomatic participants [23]. It is possible that in the absence of diagnostic tests and in-depth knowledge of participants’ medical history, several clinical diagnoses such as a bursa, prominent medial eminence of the first metatarsal, or bony swelling in joints with osteoarthritis can be misclassified as hallux valgus. However, these conditions are relatively rare in a general population and thus misclassification of these conditions likely had little effect on association results obtained from our meta-analysis. Importantly, another strength of our study is that it was not based on clinical cases only, but rather on a general population and therefore not affected by selection bias.

Our results should be interpreted in light of several limitations. First, hallux valgus was assessed across cohorts in two different ways (angular criteria vs. Manchester grading scale), which may lead to phenotypic misclassification and potential loss of statistical power. However, we assessed the distributions of the phenotype by cohort and compared distributions of key factors like age, sex, and BMI to ensure that there were no major differences. In all studies, participants categorized as ‘hallux valgus present’ were slightly older and were more likely to be female than those categorized as ‘no deformity’. As we noted previously, hallux valgus was less prevalent in FHS than in GOGO, JoCo, and OAI. This can be explained by the fact that FHS is a geographically-defined cohort study which did not specifically select individuals with or at risk of OA unlike OAI and GOGO. In addition, the lower prevalence of hallux valgus in FHS can be attributed to 1) differences in BMI and sex distributions and 2) environmental risk factors shared by family members leading to the development or prevention of hallux valgus [54]. Despite efforts to minimize bias and ensure that hallux valgus was classified using a comparable method to JoCo, GOGO, and FHS as described by Menz and others, heterogeneity resulting from pooling data across studies may still be present and we can only speculate how results would change if the OAI cohort had been assessed for hallux valgus using angular criteria. We note though that it is unlikely that our primary findings were driven by OAI or any single study since I^2^ values were low and showed little evidence for study heterogeneity. Misclassification is a potential problem in the OAI where participants have less severe forms of the condition. Participants with mild deformity, however, were excluded from our main analyses, and including these participants in the sensitivity analysis did not affect our novel findings. Overall, any misclassification and heterogeneity would likely bias associations toward the null and would not affect our findings, but may limit power for additional discoveries. Second, we were unable to assess the severity of hallux valgus because we were limited by the measurements available in the participating studies. As noted previously, using ordinal measurements of hallux valgus such as the Manchester grade can improve the statistical power compared to a dichotomous trait such as hallux valgus presence or absence [10, 22]. Third, we were unable to replicate our findings in a different independent population with a comparable level of phenotyping. To the best of our knowledge, there are no other Caucasian cohorts with well-defined hallux valgus phenotypes and genome-wide genotyping. In the UK Biobank data that we used for replication, the lead variant was not associated with hallux valgus. This may be explained in part by the use of different phenotype criteria and different statistical models (logistic vs. linear regression, BMI adjustment). The prevalence of hallux valgus was much lower (~ 2%) in the UK Biobank compared to our meta-analysis (31–48%). Replication of our findings in additional studies with identical phenotype criteria and design will be important in the future. Fourth, we did not evaluate whether our findings are generalizable to individuals of other ancestry groups. We included only participants of European Ancestry in the analyses. Although GWAS data were available for 600 African American (AA) participants (268 from OAI and 332 from JoCoOA), we did not perform meta-analysis on AA samples due to a small sample size and limited statistical power.

In conclusion, we reported the largest hallux valgus meta-analysis on individuals of European ancestry. Hallux valgus is a common foot disorder that is greatly understudied, particularly its possible genetic aspects. Building upon prior work, we aimed to identify novel genetic variants associated with hallux valgus, and found a novel variant in the gene *CLCA2*. In addition, our top-hits in *CLCA2* are eQTLs for a neighboring *COL24A1* gene and potentially pinpoint the true gene of interest from an associated locus. While observed results were attenuated and signal diminished in sex-specific analyses, this study provides new insights into hallux valgus biology and the findings for additional replication and functional follow-up.

## Supplementary information

**Additional file 1 Supplementary Fig. 1.** Manhattan plot for sensitivity meta-analysis of GWAS of hallux valgus, total sample. **Supplementary Fig. 2.** Tissue expression analysis of 30 general tissues. **Supplementary Fig. 3.** Tissue expression analysis of 53 specific tissues. **Supplementary Table 1.** Details on genotyping in each cohort. **Supplementary Table 2** (total)**. Supplementary Table 3** (sensitivity total)**. Supplementary Table 4** (men)**. Supplementary Table 5** (women)**. Supplementary Table 6.** The results of gene analysis generated by FUMA. **Supplementary Table 7.** The results of gene set analysis generated by FUMA**.**

## Data Availability

JoCo and GOGO are not publicly available data sources, and thus permission from the principal investigators is required for obtaining data (golight@email.unc.edu, amanda_nelson@med.unc.edu). OAI data is publicly available at https://data-archive.nimh.nih.gov/oai/. Participant-level phenotype and genotype data from the Framingham Heart Study are accessible from the U.S. National Center for Biotechnology Information (NCBI) database of Genotypes and Phenotypes (dbGaP) at https://dbgap.ncbi.nlm.nih.gov/ to approved scientific investigators pursuing research questions that are consistent with the informed consent agreements provided by individual research participants.
