# Supplementary material for: Genome-wide meta-analysis identified novel variant associated with hallux valgus in Caucasians
Source: J Foot Ankle Res. 2020 Mar 4;13:11. doi: 10.1186/s13047-020-0379-1 (PMC7057609; doi:10.1186/s13047-020-0379-1)
Supplement: Supplementary file 1 — Additional file 1 Supplementary Fig. 1. Manhattan plot for sensitivity meta-analysis of GWAS of hallux valgus, total sample. Supplementary Fig. 2. Tissue expression analysis of 30 general tissues. Supplementary Fig. 3. Tissue expression analysis of 53 specific tissues. Supplementary Table 1. Details on genotyping in each cohort. Supplementary Table 2 (total). Supplementary Table 3 (sensitivity total). Supplementary Table 4 (men). Supplementary Table 5 (women). Supplementary Table 6. The results of gene analysis generated by FUMA. Supplementary Table 7. The results of gene set analysis generated by FUMA. [file 13047_2020_379_MOESM1_ESM.docx]

**Supplementary Figure 1.** Manhattan plot for sensitivity meta-analysis of GWAS of hallux valgus, total sample.


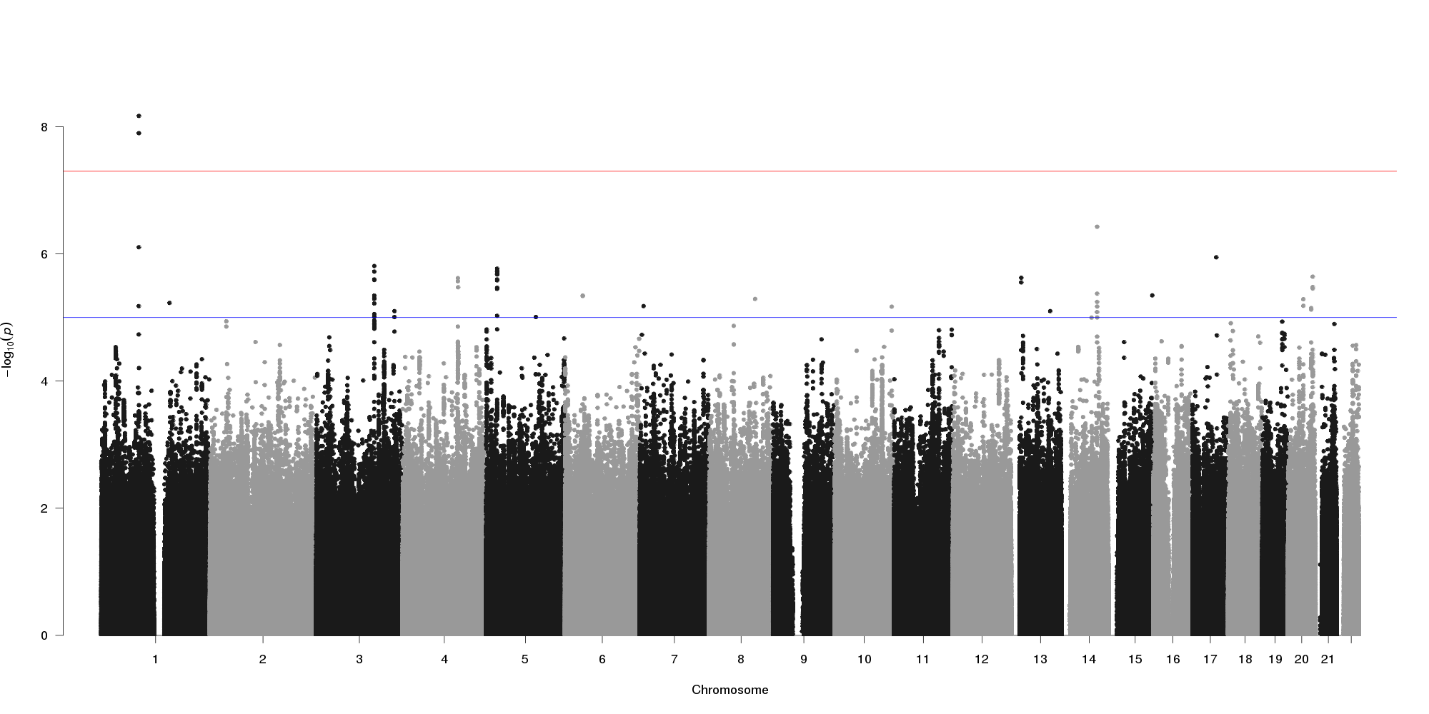


**Supplementary Figure 2.** Tissue expression analysis of 30 general tissues


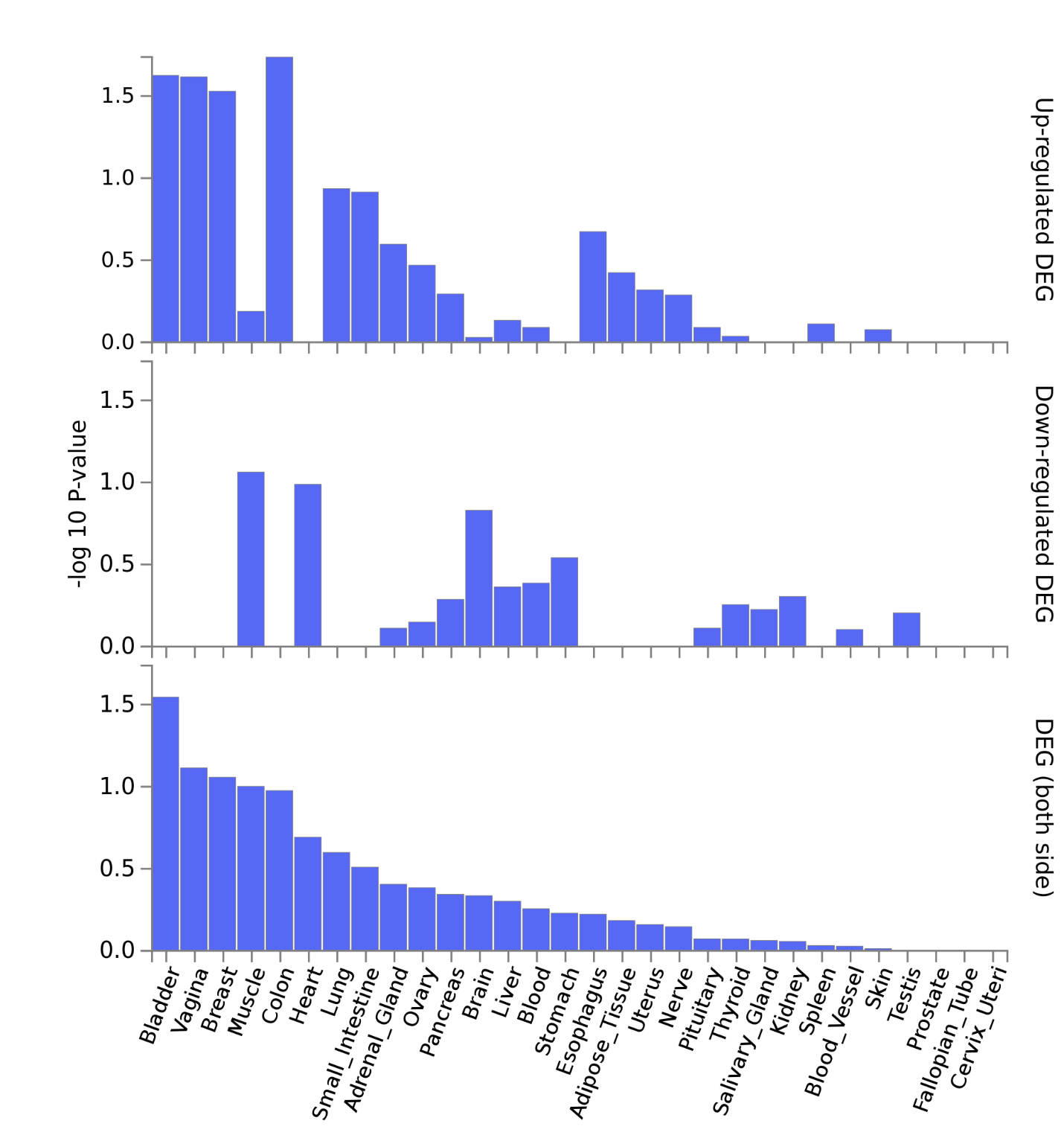


**Supplementary Figure 3.** Tissue expression analysis of 53 specific tissues

**
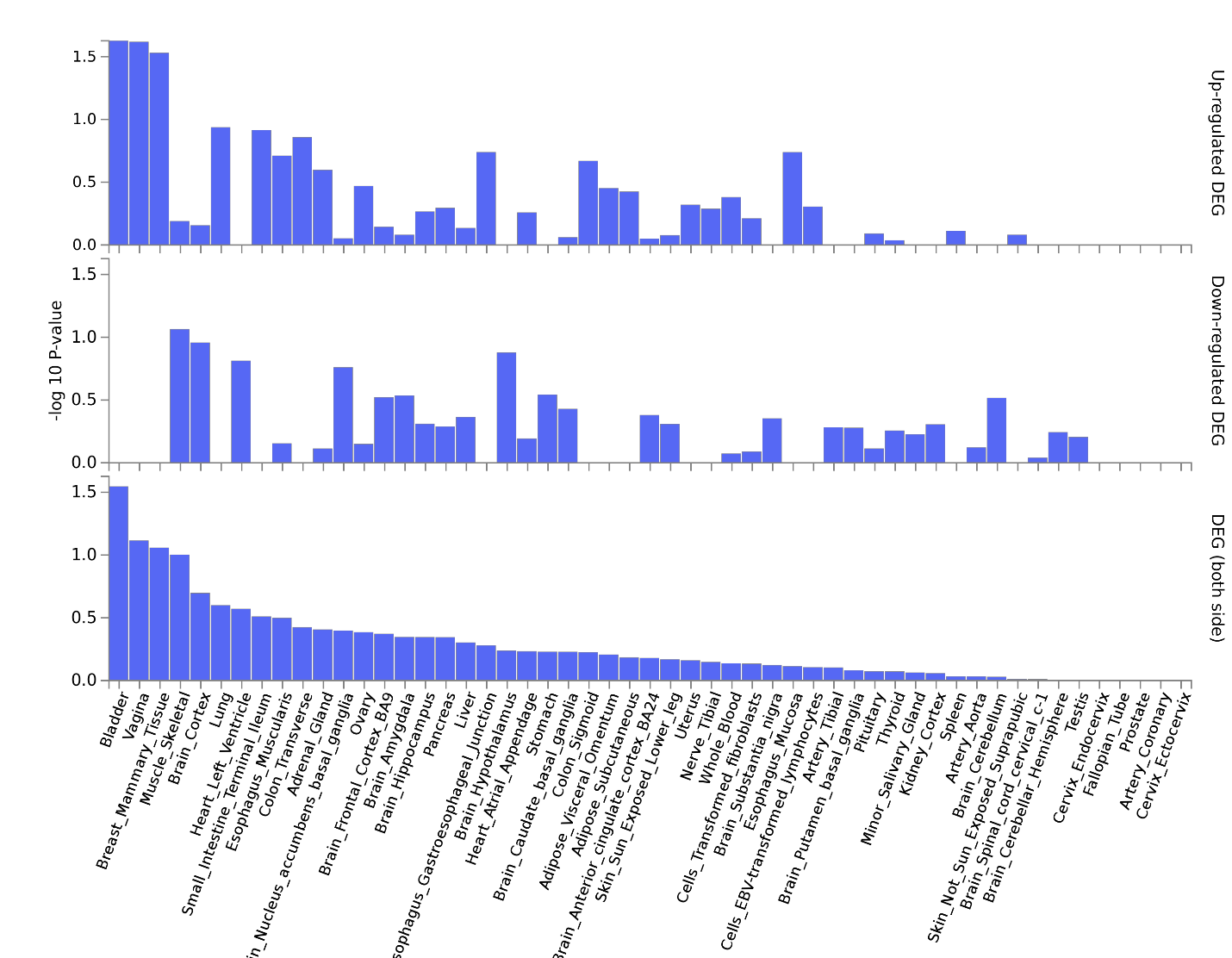
**

**Supplementary Table 1. Details on genotyping in each cohort.**

| **Cohort** | **Platform** | **Genotype calling Software** | **Sample** | **Marker** |  |  |
| --- | --- | --- | --- | --- | --- | --- |
|  |  |  | **Call Rate** | **Call Rate** | **HWE P-value** | **MAF** |
| *FHS* | Affymetrix 500K DualGeneChip+ 50K gene-centered MIP set | BRLMM | 99% | 96.90% | ≥10-6 | 1% |
| *JoCo* | Illumina IM - Duo | Illumina BeadStudio | 95% | 98% | ≥10-6 | 1% |
| *GOGO* | Illumina HumanHap 610 Quad v1 + custom SNPs | Illumina BeadStudio | 99% | 99% | ≥10-6 | 1% |
| *OAI* | Illumina Omni-Quad 2.5 M | Illumina BeadStudio | 99% | 99% | ≥10-6 | 1% |

**Supplementary Table 2 (total)**

| SNP rsID | Chr:Position | Nearest Gene (hgnc) | EA/OA | EAF | OR (95% CI) | P-value | Direction | I2 | Het. P-value |
| --- | --- | --- | --- | --- | --- | --- | --- | --- | --- |
| **rs55807512** | 1:86916200 | **CLCA2** | t/c | 0.96 | 0.48 (0.38, 0.61) | 2.96E-09 | ---- | 0 | 0.67 |
| **rs12124247** | 1:86890244 | **CLCA2** | a/g | 0.03 | 2.19 (1.68, 2.86) | 7.38E-09 | ++++ | 0 | 0.68 |
| rs146496015* | 1:86799387 | ODF2L | t/g | 0.06 | 1.61 (1.34, 1.94) | 3.14E-07 | ++++ | 1.6 | 0.38 |
| **rs12888772** | 14:81578926 | **RP11-114N19.3** | a/t | 0.18 | 1.30 (1.17, 1.44) | 7.92E-07 | ++++ | 0 | 0.96 |
| **rs117155606** | 17:54325272 | **ANKFN1** | t/c | 0.01 | 5.53 (2.78, 11.02) | 1.13E-06 | ?+??? | 0 | 1 |
| rs11728629 | 4:126712045 | RP11-404I7.1 | a/t | 0.24 | 1.26 (1.15, 1.38) | 1.28E-06 | ++++ | 0 | 0.8 |
| rs4314298 | 4:126710287 | RP11-404I7.1 | a/g | 0.76 | 0.80 (0.73, 0.87) | 1.42E-06 | ---- | 0 | 0.82 |
| rs116783165 | 5:25669440 | CTD-2533K21.1 | c/g | 0.03 | 1.72 (1.37, 2.15) | 1.95E-06 | ++++ | 58.6 | 0.06 |
| rs116336450 | 5:25663890 | CTD-2533K21.1 | t/c | 0.97 | 0.58 (0.47, 0.73) | 2.13E-06 | ---- | 60.5 | 0.06 |
| rs79433053 | 5:25668523 | CTD-2533K21.1 | t/c | 0.03 | 1.71 (1.37, 2.14) | 2.17E-06 | ++++ | 60.3 | 0.06 |
| rs115777891 | 5:25668049 | CTD-2533K21.1 | t/g | 0.03 | 1.71 (1.37, 2.14) | 2.18E-06 | ++++ | 60.4 | 0.06 |
| rs12143200* | 1:86644262 | COL24A1 | t/g | 0.97 | 0.56 (0.44, 0.71) | 2.28E-06 | ---- | 0 | 0.56 |
| rs76655016 | 13:23022108 | LINC00540 | a/t | 0.01 | 2.98 (1.90, 4.70) | 2.33E-06 | +++? | 0 | 0.46 |
| rs17546846 | 5:25659404 | CTD-2533K21.1 | t/c | 0.97 | 0.59 (0.47, 0.73) | 2.36E-06 | ---- | 61.1 | 0.05 |
| rs115780600 | 5:25659570 | CTD-2533K21.1 | a/g | 0.97 | 0.59 (0.47, 0.73) | 2.39E-06 | ---- | 61.2 | 0.05 |
| rs114356118 | 5:25659658 | CTD-2533K21.1 | t/c | 0.97 | 0.59 (0.47, 0.73) | 2.39E-06 | ---- | 61.2 | 0.05 |
| rs2067904 | 5:25672303 | CTD-2533K21.1 | t/c | 0.97 | 0.58 (0.47, 0.73) | 2.43E-06 | ---- | 59.8 | 0.06 |
| rs58302722 | 4:127415516 | RP11-404I7.1 | a/g | 0.02 | 1.82 (1.42, 2.34) | 2.52E-06 | ++++ | 0 | 0.50 |
| rs118167637 | 13:23024261 | LINC00540 | t/g | 0.99 | 0.34 (0.21, 0.53) | 2.78E-06 | ---? | 9 | 0.33 |
| rs2877504 | 5:25655531 | CTD-2533K21.1 | t/c | 0.03 | 1.69 (1.36, 2.10) | 2.86E-06 | ++++ | 60.4 | 0.06 |
| rs142096460 | 5:25664847 | CTD-2533K21.1 | a/t | 0.03 | 1.70 (1.36, 2.12) | 2.89E-06 | ++++ | 62.2 | 0.05 |
| **rs80177909** | 20:35389639 | **DSN1** | a/g | 0.98 | 2.17 (1.57, 3.00) | 3.13E-06 | ++++ | 0 | 0.51 |
| **rs77993129** | 18:6325546 | **L3MBTL4** | t/c | 0.97 | 1.84 (1.42, 2.38) | 3.82E-06 | ++++ | 0 | 0.64 |
| rs17546596 | 5:25646613 | CTD-2533K21.1 | t/g | 0.97 | 0.60 (0.48, 0.74) | 3.87E-06 | ---- | 60.4 | 0.06 |
| rs147360091 | 5:25653897 | CTD-2533K21.1 | a/g | 0.97 | 0.60 (0.48, 0.74) | 3.87E-06 | ---- | 60.6 | 0.05 |
| **rs4811981*** | 20:56871088 | **PPP4R1L** | t/c | 0.7 | 0.82 (0.75, 0.89) | 3.90E-06 | ---- | 29.8 | 0.23 |
| rs17467875 | 5:25644855 | CTD-2533K21.1 | a/t | 0.03 | 1.67 (1.34, 2.08) | 4.01E-06 | ++++ | 60.5 | 0.06 |
| **rs6029512** | 20:35415440 | **SOGA1** | a/g | 0.02 | 0.46 (0.33, 0.64) | 4.12E-06 | ---- | 0 | 0.49 |
| rs183663644* | 15:100426553 | CTD-2054N24.2 | t/c | 0.99 | 0.16 (0.07, 0.35) | 4.49E-06 | ?-?? | 0 | 1.00 |
| rs113871023 | 6:40822300 | ACTG1P9 | a/t | 0.99 | 7.37 (3.14, 17.32) | 4.52E-06 | ?+?? | 0 | 1.00 |
| rs76534747 | 6:40822752 | ACTG1P9 | t/c | 0.01 | 0.14 (0.06, 0.32) | 4.59E-06 | ?-?? | 0 | 1.00 |
| rs71218579 | 13:27061500 | WASF3 | a/g | 0.68 | 1.22 (1.12, 1.33) | 4.90E-06 | ++++ | 11.5 | 0.34 |

Intron SNPs are indicated in bold

* showed nominal evidence (p<0.05) for association with HV in UK BioBank

**Supplementary Table 3 (sensitivity total)**

| SNP rsID | Chr:Position | Nearest Gene (hgnc) | EA/OA | EAF | OR (95% CI) | P-value | Direction | I2 | Het. P-value |
| --- | --- | --- | --- | --- | --- | --- | --- | --- | --- |
| **rs55807512** | 1:86916200 | **CLCA2** | t/c | 0.96 | 0.48 (0.39, 0.61) | 4.75E-10 | ---- | 0 | 0.68 |
| **rs12124247** | 1:86890244 | **CLCA2** | a/g | 0.03 | 2.16 (1.68, 2.79) | 2.39E-09 | ++++ | 0 | 0.80 |
| **rs12888772** | 14:81578926 | **TSHR** | a/t | 0.18 | 1.31 (1.19, 1.45) | 1.55E-07 | ++++ | 0 | 0.99 |
| rs146496015* | 1:86799387 | ODF2L | t/g | 0.06 | 1.56 (1.31, 1.86) | 5.25E-07 | ++++ | 0 | 0.46 |
| **rs7257226*** | 19:46123929 | **EML2** | a/g | 0.13 | 0.73 (0.64, 0.83) | 7.66E-07 | ---- | 0 | 0.52 |
| **rs12462392*** | 19:46130579 | **EML2** | t/c | 0.87 | 1.36 (1.20, 1.54) | 1.06E-06 | ++++ | 0 | 0.47 |
| **rs117155606** | 17:54325272 | **ANKFN1** | t/c | 0.01 | 5.53 (2.78, 11.02) | 1.13E-06 | ?+?? | 0 | 1.00 |
| **rs28365786*** | 19:46127918 | **EML2** | a/g | 0.87 | 1.36 (1.20, 1.54) | 1.21E-06 | ++++ | 0 | 0.41 |
| **rs34049302*** | 19:46139329 | **EML2** | a/g | 0.87 | 1.35 (1.19, 1.53) | 1.70E-06 | ++++ | 0 | 0.60 |
| **rs8113155*** | 19:46128655 | **EML2** | t/c | 0.13 | 0.74 (0.65, 0.84) | 1.92E-06 | ---- | 0 | 0.43 |
| rs12151009* | 19:46141845 | EML2 | t/c | 0.87 | 1.35 (1.19, 1.53) | 1.94E-06 | ++++ | 0 | 0.53 |
| **rs35347616*** | 19:46140982 | **EML2** | c/g | 0.87 | 1.35 (1.19, 1.53) | 2.18E-06 | ++++ | 0 | 0.45 |
| rs7251952* | 19:46133251 | EML2 | t/c | 0.13 | 0.74 (0.65, 0.84) | 2.22E-06 | ---- | 0 | 0.45 |
| rs76655016 | 13:23022108 | LINC00540 | a/t | 0.01 | 2.77 (1.82, 4.23) | 2.29E-06 | +++? | 11 | 0.33 |
| rs12143200* | 1:86644262 | COL24A1 | t/g | 0.97 | 0.57 (0.45, 0.72) | 2.36E-06 | ---- | 0 | 0.48 |
| rs7252175* | 19:46133256 | EML2 | a/g | 0.13 | 0.74 (0.66, 0.84) | 2.54E-06 | ---- | 0 | 0.40 |
| rs118167637 | 13:23024261 | LINC00540 | t/g | 0.99 | 0.37 (0.24, 0.56) | 3.26E-06 | ---? | 34.4 | 0.22 |
| **rs9669266** | 12:3665905 | **PRMT8** | a/g | 0.47 | 0.83 (0.76, 0.90) | 3.48E-06 | ---- | 55.5 | 0.08 |
| **rs9669267** | 12:3665913 | **PRMT8** | a/g | 0.47 | 0.83 (0.76, 0.90) | 3.54E-06 | ---- | 55.4 | 0.08 |
| **rs34995718*** | 19:46115114 | **EML2** | t/c | 0.14 | 0.76 (0.67, 0.85) | 3.54E-06 | ---- | 0 | 0.66 |
| **rs36219972** | 19:55173768 | **LILRB4** | a/g | 0.05 | 1.68 (1.35, 2.09) | 4.14E-06 | +++? | 24.7 | 0.27 |
| rs183663644* | 15:100426553 | CTD-2054N24.2 | t/c | 0.99 | 0.16 (0.07, 0.35) | 4.49E-06 | ?-?? | 0 | 1.00 |
| rs113871023 | 6:40822300 | ACTG1P9 | a/t | 0.99 | 7.37 (3.14, 17.32) | 4.52E-06 | ?+?? | 0 | 1.00 |
| rs76534747 | 6:40822752 | ACTG1P9 | t/c | 0.01 | 0.14 (0.06, 0.32) | 4.59E-06 | ?-?? | 0 | 1.00 |

Intron SNPs are indicated in bold

* showed nominal evidence (p<0.05) for association with HV in UK BioBank

**Supplementary Table 4 (men)**

| SNP rsID | Chr:Position | Nearest Gene (hgnc) | EA/OA | EAF | OR (95% CI) | P-value | Direction | I2 | Het. P-value |
| --- | --- | --- | --- | --- | --- | --- | --- | --- | --- |
| rs141161671 | 2:52279316 | AC007682.1 | a/g | 0.01 | 6.50 (3.35, 12.62) | 3.22E-08 | ???+ | 0 | 1.00 |
| rs28395286 | 8:81327517 | RNU6-1213P | t/c | 0.05 | 2.07 (1.55, 2.76) | 7.01E-07 | ++++ | 0 | 1.00 |
| rs1859504 | 7:9124935 | RP4-668E10.4 | t/c | 0.68 | 0.68 (0.58, 0.79) | 1.16E-06 | ---- | 0 | 0.47 |
| rs12619631 | 2:117372907 | RP11-338I24.1 | a/g | 0.15 | 1.59 (1.32, 1.92) | 1.47E-06 | ++++ | 0 | 0.88 |
| rs72762109 | 1:236960697 | MTR | a/g | 0.99 | 0.06 (0.02, 0.20) | 1.52E-06 | ???- | 0 | 1.00 |
| rs17554213 | 2:117364747 | RP11-338I24.1 | t/c | 0.84 | 0.64 (0.53, 0.77) | 1.95E-06 | ---- | 0 | 0.94 |
| rs77038451 | 2:117359849 | RP11-338I24.1 | a/g | 0.15 | 1.57 (1.30, 1.90) | 2.36E-06 | ++++ | 0 | 0.91 |
| rs74863661 | 5:168297191 | SLIT3 | t/c | 0.15 | 1.57 (1.30, 1.89) | 2.47E-06 | ++++ | 0 | 0.77 |
| rs78762426 | 6:104721993 | RP3-359N14.1 | a/g | 0.08 | 1.89 (1.45, 2.46) | 2.50E-06 | ++++ | 0 | 0.98 |
| rs10176968 | 2:40036967 | SLC8A1-AS1 | a/g | 0.39 | 1.40 (1.22, 1.61) | 2.57E-06 | ++++ | 0 | 0.53 |
|  | 18:50130115 |  | a/g | 0.02 | 3.82 (2.18, 6.67) | 2.60E-06 | +?++ | 54.5 | 0.11 |
| rs6745380 | 2:40034051 | SLC8A1-AS1 | a/t | 0.61 | 0.71 (0.62, 0.82) | 2.62E-06 | ---- | 0 | 0.51 |
| rs112331738 | 6:104693747 | RP3-359N14.1 | a/c | 0.92 | 0.53 (0.41, 0.69) | 3.12E-06 | ---- | 0 | 0.96 |
| rs75358387 | 6:104705139 | RP3-359N14.1 | t/c | 0.92 | 0.53 (0.41, 0.70) | 3.13E-06 | ---- | 0 | 0.97 |
| rs191124187 | 21:41903851 | DSCAM | c/g | 0.01 | 10.71 (3.95, 29.03) | 3.18E-06 | ???+ | 0 | 1.00 |
| rs11868764 | 17:72064864 | LINC02074 | a/g | 0.97 | 0.34 (0.22, 0.53) | 3.22E-06 | ---- | 2.3 | 0.38 |
| rs2373698 | 2:40034977 | SLC8A1-AS1 | t/c | 0.61 | 0.72 (0.62, 0.83) | 3.31E-06 | ---- | 0 | 0.53 |
| rs13147821 | 4:80671379 | RP11-576N17.4 | a/t | 0.74 | 0.69 (0.59, 0.81) | 3.33E-06 | ---- | 26.3 | 0.25 |
| rs12618364 | 2:117390763 | RP11-338I24.1 | a/g | 0.15 | 1.57 (1.30, 1.89) | 3.34E-06 | ++++ | 0 | 0.86 |
| rs188650377 | 16:10286637 | GRIN2A | a/t | 0.99 | 0.07 (0.02, 0.22) | 3.39E-06 | ???- | 0 | 1.00 |
| rs386645199 | 2:40034674 | SLC8A1-AS1 | t/c | 0.39 | 1.39 (1.21, 1.60) | 3.79E-06 | ++++ | 0 | 0.54 |
| rs60891386 | 2:117382035 | RP11-338I24.1 | t/g | 0.16 | 1.55 (1.29, 1.87) | 4.06E-06 | ++++ | 0 | 0.94 |
| rs117798261 | 18:50044745 | DCC | t/c | 0.02 | 3.44 (2.03, 5.83) | 4.17E-06 | +?++ | 72 | 0.03 |
| rs142973841 | 12:124648832 | RFLNA | t/c | 0.02 | 3.00 (1.88, 4.80) | 4.35E-06 | -+?+ | 95.5 | 0.00 |
| rs347133 | 3:32442673 | CMTM7 | a/g | 0.74 | 0.69 (0.59, 0.81) | 4.35E-06 | -+-- | 39 | 0.18 |
| rs2110671 | 2:40035535 | SLC8A1-AS1 | t/c | 0.39 | 1.39 (1.21, 1.60) | 4.41E-06 | ++++ | 0 | 0.47 |
| rs6733149 | 2:51836284 | AC007682.1 | a/c | 0.99 | 0.18 (0.09, 0.37) | 4.50E-06 | ???- | 0 | 1.00 |
|  | 8:52512282 |  | t/g | 0.99 | 0.15 (0.07, 0.34) | 4.56E-06 | ???- | 0 | 1.00 |
| rs75323088 | 2:117358765 | RP11-338I24.1 | t/g | 0.83 | 0.66 (0.55, 0.79) | 4.97E-06 | ---- | 0 | 0.95 |

**Supplementary Table 5 (women)**

| SNP rsID | Chr:Position | Nearest Gene (hgnc) | EA/OA | EAF | OR (95% CI) | P-value | Direction | I2 | Het. P-value |
| --- | --- | --- | --- | --- | --- | --- | --- | --- | --- |
| rs140676665 | 2:199519654 | AC019330.1 | a/g | 0.01 | 0.14 (0.07, 0.29) | 1.41E-07 | ?-?- | 91.4 | 0.001 |
| rs9850315 | 3:177148591 | U8 | c/g | 0.02 | 2.43 (1.73, 3.41) | 3.42E-07 | ++-+ | 60.3 | 0.056 |
| rs9851118 | 3:177143248 | U8 | t/c | 0.02 | 2.42 (1.72, 3.39) | 3.63E-07 | ++-+ | 57.1 | 0.072 |
| rs9856070 | 3:177143882 | U8 | a/g | 0.02 | 2.42 (1.72, 3.40) | 3.64E-07 | ++-+ | 57.9 | 0.068 |
| rs9851138 | 3:177143296 | U8 | t/c | 0.02 | 2.42 (1.72, 3.39) | 3.69E-07 | ++-+ | 57.6 | 0.070 |
| rs9810245 | 3:177151247 | U8 | a/t | 0.02 | 2.41 (1.72, 3.39) | 4.17E-07 | ++-+ | 60.5 | 0.055 |
| rs9850245 | 3:177148733 | U8 | c/g | 0.98 | 0.41 (0.29, 0.58) | 4.41E-07 | --+- | 59.6 | 0.060 |
| rs139705512 | 2:192160970 | MYO1B | a/g | 0.02 | 3.05 (1.97, 4.70) | 4.98E-07 | ++++ | 0 | 0.768 |
| rs150013648 | 8:105459422 | DPYS | t/c | 0.02 | 3.43 (2.12, 5.56) | 5.21E-07 | +?++ | 0 | 0.787 |
| rs9877042 | 3:177153670 | U8 | c/g | 0.02 | 2.40 (1.70, 3.38) | 5.48E-07 | ++-+ | 59.6 | 0.060 |
| rs539720802 | 19:42398954 | ARHGEF1 | t/g | 0.01 | 0.12 (0.05, 0.27) | 5.60E-07 | ???- | 0 | 1.000 |
| rs145273870 | 2:199514362 | AC019330.1 | c/g | 0.01 | 0.15 (0.07, 0.32) | 5.84E-07 | ?-?- | 91.8 | 0.000 |
| rs79147928 | 22:49005909 | FAM19A5 | a/g | 0.14 | 0.65 (0.55, 0.77) | 7.59E-07 | ---- | 20.5 | 0.287 |
| rs117568517 | 19:42398949 | ARHGEF1 | t/c | 0.99 | 7.40 (3.32, 16.48) | 9.77E-07 | ???+ | 0 | 1.000 |
| rs77247192 | 22:49005638 | FAM19A5 | a/g | 0.12 | 0.64 (0.53, 0.77) | 1.18E-06 | ---- | 46.6 | 0.132 |
| rs3891969 | 6:170075736 | WDR27 | a/g | 0.15 | 1.40 (1.22, 1.61) | 1.68E-06 | ++++ | 48.1 | 0.123 |
| rs116712252 | 6:64596749 | EYS | a/g | 0.01 | 0.09 (0.03, 0.24) | 1.70E-06 | ?-?+ | 95.5 | 0.000 |
| rs55807512 | 1:86916200 | CLCA2 | t/c | 0.96 | 0.47 (0.35, 0.64) | 1.73E-06 | ---- | 35.9 | 0.197 |
| rs117974989 | 11:132575991 | OPCML | a/g | 0.01 | 0.33 (0.21, 0.52) | 2.12E-06 | --?- | 86.4 | 0.001 |
| rs145025296 | 2:192292708 | MYO1B | t/c | 0.98 | 0.30 (0.18, 0.49) | 2.30E-06 | --?- | 0 | 0.404 |
| rs117417356 | 11:132574165 | OPCML | t/c | 0.99 | 3.00 (1.90, 4.73) | 2.61E-06 | ++?+ | 86.3 | 0.001 |
| rs77473284 | 11:132574928 | OPCML | t/g | 0.99 | 2.99 (1.89, 4.73) | 2.67E-06 | ++?+ | 86.3 | 0.001 |
| rs28413842 | 22:49002819 | FAM19A5 | a/g | 0.13 | 0.66 (0.56, 0.79) | 3.46E-06 | ---- | 10.9 | 0.338 |
| rs140687271 | 13:63340985 | LINC00448 | a/c | 0.01 | 0.13 (0.05, 0.31) | 4.51E-06 | ?-?- | 63 | 0.100 |
| rs2631419 | 11:24767384 | LUZP2 | t/c | 0.32 | 1.29 (1.16, 1.44) | 4.56E-06 | ++++ | 0 | 0.815 |
| rs11707593 | 3:181462478 | SOX2-OT | t/c | 0.24 | 1.32 (1.17, 1.49) | 4.77E-06 | ++++ | 6.4 | 0.361 |
| rs2631418 | 11:24768199 | LUZP2 | c/g | 0.68 | 0.77 (0.69, 0.86) | 4.85E-06 | ---- | 0 | 0.833 |
| rs1345416 | 3:181462046 | SOX2-OT | a/g | 0.77 | 0.76 (0.67, 0.85) | 4.85E-06 | ---- | 0 | 0.416 |

**Supplementary Table 6. The results of gene analysis generated by FUMA**

| GENE | CHR | START | STOP | NSNPS | NPARAM | N | ZSTAT | P | SYMBOL |
| --- | --- | --- | --- | --- | --- | --- | --- | --- | --- |
| ENSG00000176783 | 5 | 178977559 | 179037027 | 175 | 23 | 5925 | 4.4253 | 4.82E-06 | RUFY1 |
| ENSG00000133466 | 22 | 37576207 | 37595425 | 78 | 11 | 5925 | 4.0127 | 3.00E-05 | C1QTNF6 |
| ENSG00000185739 | 16 | 4239375 | 4292081 | 228 | 40 | 5925 | 4.0015 | 3.15E-05 | SRL |
| ENSG00000101084 | 20 | 35234137 | 35240960 | 10 | 5 | 5925 | 3.9701 | 3.59E-05 | C20orf24 |
| ENSG00000259399 | 20 | 35202956 | 35240787 | 39 | 17 | 5925 | 3.9414 | 4.05E-05 | TGIF2-C20orf24 |
| ENSG00000125965 | 20 | 34021145 | 34042568 | 36 | 9 | 5925 | 3.9185 | 4.45E-05 | GDF5 |
| ENSG00000148600 | 10 | 85954410 | 85979377 | 87 | 15 | 5925 | 3.8751 | 5.33E-05 | CDHR1 |
| ENSG00000135697 | 16 | 81272053 | 81324747 | 201 | 30 | 5925 | 3.7593 | 8.52E-05 | BCMO1 |
| ENSG00000111218 | 12 | 3490515 | 3703139 | 562 | 93 | 5925 | 3.7339 | 9.43E-05 | PRMT8 |
| ENSG00000016490 | 1 | 86934051 | 86965972 | 121 | 27 | 5925 | 3.6739 | 0.00011943 | CLCA1 |
| ENSG00000124224 | 20 | 56806188 | 56884495 | 222 | 20 | 5925 | 3.6652 | 0.00012357 | PPP4R1L |
| ENSG00000143643 | 1 | 231041989 | 231114621 | 261 | 19 | 5925 | 3.6299 | 0.00014174 | TTC13 |
| ENSG00000101019 | 20 | 33890369 | 33999944 | 183 | 12 | 5925 | 3.6279 | 0.00014286 | UQCC1 |
| ENSG00000197241 | 1 | 9063359 | 9086404 | 75 | 22 | 5925 | 3.6258 | 0.00014405 | SLC2A7 |
| ENSG00000169045 | 5 | 179041179 | 179061785 | 54 | 12 | 5925 | 3.5999 | 0.00015919 | HNRNPH1 |
| ENSG00000117862 | 1 | 52485803 | 52521843 | 63 | 8 | 5925 | 3.5373 | 0.00020208 | TXNDC12 |
| ENSG00000150625 | 4 | 176554085 | 176923815 | 1187 | 93 | 5925 | 3.4997 | 0.00023291 | GPM6A |
| ENSG00000158201 | 18 | 19230858 | 19284766 | 85 | 11 | 5925 | 3.4617 | 0.00026836 | ABHD3 |
| ENSG00000123636 | 2 | 160175490 | 160473203 | 889 | 57 | 5925 | 3.457 | 0.00027308 | BAZ2B |
| ENSG00000105229 | 19 | 4007644 | 4039384 | 112 | 13 | 5925 | 3.4484 | 0.00028196 | PIAS4 |
| ENSG00000176659 | 20 | 58630980 | 58648008 | 41 | 7 | 5925 | 3.4467 | 0.00028369 | C20orf197 |
| ENSG00000198841 | 1 | 52497775 | 52499488 | 3 | 1 | 5925 | 3.4287 | 0.00030321 | KTI12 |
| ENSG00000124209 | 20 | 56884752 | 56942563 | 189 | 25 | 5925 | 3.4033 | 0.00033295 | RAB22A |
| ENSG00000204217 | 2 | 203241659 | 203432474 | 339 | 25 | 5925 | 3.3864 | 0.00035403 | BMPR2 |
| ENSG00000186260 | 16 | 14165178 | 14360630 | 465 | 16 | 5925 | 3.3795 | 0.00036311 | MKL2 |
| ENSG00000180758 | 1 | 9160364 | 9189229 | 78 | 12 | 5925 | 3.3726 | 0.00037226 | GPR157 |
| ENSG00000223638 | 19 | 56270380 | 56274541 | 9 | 3 | 5925 | 3.3681 | 0.0003784 | RFPL4A |
| ENSG00000153551 | 3 | 32433163 | 32524559 | 363 | 43 | 5925 | 3.367 | 0.00038 | CMTM7 |
| ENSG00000065491 | 6 | 37225548 | 37300746 | 150 | 14 | 5925 | 3.363 | 0.00038548 | TBC1D22B |
| ENSG00000204183 | 20 | 34020827 | 34023248 | 5 | 1 | 5925 | 3.3602 | 0.00038947 | GDF5OS |
| ENSG00000104522 | 8 | 144694788 | 144700218 | 20 | 5 | 5925 | 3.3425 | 0.00041515 | TSTA3 |
| ENSG00000107719 | 10 | 72238577 | 72328205 | 410 | 48 | 5925 | 3.3219 | 0.00044698 | PALD1 |
| ENSG00000213741 | 14 | 50043390 | 50065408 | 46 | 7 | 5925 | 3.3125 | 0.00046229 | RPS29 |
| ENSG00000253459 | 14 | 50064193 | 50065882 | 6 | 3 | 5925 | 3.2739 | 0.00053044 | AL139099.1 |
| ENSG00000131203 | 8 | 39759794 | 39785963 | 52 | 11 | 5925 | 3.2687 | 0.0005403 | IDO1 |
| ENSG00000149636 | 20 | 35380194 | 35402221 | 19 | 13 | 5925 | 3.2518 | 0.00057347 | DSN1 |
| ENSG00000172738 | 6 | 37179956 | 37225931 | 70 | 16 | 5925 | 3.2029 | 0.00068036 | TMEM217 |
| ENSG00000256463 | 18 | 76740275 | 76762677 | 50 | 12 | 5925 | 3.1715 | 0.00075828 | SALL3 |
| ENSG00000114480 | 3 | 81538850 | 81811312 | 491 | 36 | 5925 | 3.1688 | 0.00076525 | GBE1 |
| ENSG00000101079 | 20 | 35280169 | 35374481 | 100 | 38 | 5925 | 3.153 | 0.00080803 | NDRG3 |
| ENSG00000175202 | 15 | 72968124 | 72978490 | 17 | 6 | 5925 | 3.1474 | 0.00082379 | HIGD2B |
| ENSG00000196417 | 19 | 53893046 | 53930574 | 198 | 30 | 5925 | 3.1253 | 0.00088797 | ZNF765 |
| ENSG00000183473 | 22 | 37600278 | 37608362 | 20 | 6 | 5925 | 3.1145 | 0.0009213 | SSTR3 |
| ENSG00000106682 | 7 | 73588575 | 73611431 | 23 | 9 | 5925 | 3.1091 | 0.00093821 | EIF4H |
| ENSG00000112742 | 6 | 80713604 | 80752244 | 150 | 12 | 5925 | 3.1027 | 0.00095877 | TTK |
| ENSG00000177409 | 7 | 92759368 | 92777682 | 37 | 12 | 5925 | 3.1023 | 0.0009602 | SAMD9L |
| ENSG00000122707 | 9 | 36036430 | 36124448 | 162 | 22 | 5925 | 3.0715 | 0.001065 | RECK |
| ENSG00000214954 | 8 | 92114060 | 92231464 | 300 | 15 | 5925 | 3.0668 | 0.0010817 | LRRC69 |
| ENSG00000236027 | 11 | 125658006 | 125661495 | 9 | 4 | 5925 | 3.0601 | 0.0011064 | PATE3 |
| ENSG00000100109 | 22 | 26887191 | 26908471 | 63 | 17 | 5925 | 3.0566 | 0.0011192 | TFIP11 |
| ENSG00000178172 | 5 | 147582357 | 147594700 | 12 | 5 | 5925 | 3.0555 | 0.0011235 | SPINK6 |
| ENSG00000179397 | 1 | 244617679 | 244804479 | 587 | 29 | 5925 | 3.0505 | 0.0011424 | C1orf101 |
| ENSG00000140650 | 16 | 8882680 | 8943188 | 275 | 25 | 5925 | 2.9881 | 0.0014036 | PMM2 |
| ENSG00000167088 | 18 | 19192228 | 19210417 | 27 | 6 | 5925 | 2.9843 | 0.0014212 | SNRPD1 |
| ENSG00000187801 | 1 | 40915774 | 40929390 | 52 | 16 | 5925 | 2.9733 | 0.001473 | ZFP69B |
| ENSG00000135362 | 11 | 36317838 | 36486754 | 662 | 68 | 5925 | 2.9639 | 0.001519 | PRR5L |
| ENSG00000140463 | 15 | 72978527 | 73030817 | 105 | 19 | 5925 | 2.9524 | 0.0015766 | BBS4 |
| ENSG00000141446 | 18 | 19109242 | 19180845 | 81 | 11 | 5925 | 2.9433 | 0.0016237 | ESCO1 |
| ENSG00000205147 | 19 | 4041127 | 4043154 | 10 | 4 | 5925 | 2.9371 | 0.0016563 | AC016586.1 |
| ENSG00000167173 | 15 | 75487984 | 75504510 | 13 | 5 | 5925 | 2.9353 | 0.0016661 | C15orf39 |
| ENSG00000143228 | 1 | 163236366 | 163325554 | 415 | 31 | 5925 | 2.9302 | 0.0016938 | NUF2 |
| ENSG00000050555 | 9 | 133884469 | 133969860 | 319 | 43 | 5925 | 2.9071 | 0.0018238 | LAMC3 |
| ENSG00000177879 | 5 | 115177178 | 115249778 | 145 | 23 | 5925 | 2.8982 | 0.0018764 | AP3S1 |
| ENSG00000165506 | 14 | 50091892 | 50101948 | 34 | 5 | 5925 | 2.8884 | 0.0019363 | DNAAF2 |
| ENSG00000166557 | 15 | 79603404 | 79704334 | 384 | 25 | 5925 | 2.8703 | 0.0020505 | TMED3 |
| ENSG00000159763 | 7 | 142829170 | 142836839 | 18 | 5 | 5925 | 2.8697 | 0.0020543 | PIP |
| ENSG00000118707 | 20 | 35201891 | 35222353 | 17 | 9 | 5925 | 2.862 | 0.0021046 | TGIF2 |
| ENSG00000150054 | 10 | 28339922 | 28623415 | 1238 | 46 | 5925 | 2.833 | 0.0023056 | MPP7 |
| ENSG00000069206 | 8 | 24298443 | 24384483 | 231 | 27 | 5925 | 2.8252 | 0.0023629 | ADAM7 |
| ENSG00000204033 | 10 | 85980254 | 85985345 | 21 | 6 | 5925 | 2.821 | 0.0023933 | LRIT2 |
| ENSG00000112685 | 6 | 485133 | 693117 | 720 | 58 | 5925 | 2.8134 | 0.0024509 | EXOC2 |
| ENSG00000134686 | 1 | 33789224 | 33896653 | 304 | 21 | 5925 | 2.8121 | 0.0024609 | PHC2 |
| ENSG00000267901 | 14 | 71683031 | 71683462 | 1 | 1 | 5925 | 2.8075 | 0.002496 | AC004817.1 |
| ENSG00000006744 | 17 | 12895708 | 12921504 | 153 | 8 | 5925 | 2.8052 | 0.0025144 | ELAC2 |
| ENSG00000172531 | 11 | 67165654 | 67188654 | 43 | 8 | 5925 | 2.8015 | 0.0025431 | PPP1CA |
| ENSG00000101082 | 20 | 35240721 | 35274619 | 41 | 21 | 5925 | 2.8006 | 0.0025501 | SLA2 |
| ENSG00000268898 | 2 | 40488147 | 40490117 | 11 | 4 | 5925 | 2.7993 | 0.002561 | AC007377.1 |
| ENSG00000148602 | 10 | 85991349 | 86001217 | 35 | 7 | 5925 | 2.7979 | 0.002572 | LRIT1 |
| ENSG00000182986 | 19 | 53367043 | 53400946 | 134 | 19 | 5925 | 2.7942 | 0.0026017 | ZNF320 |
| ENSG00000198538 | 19 | 53300662 | 53360853 | 270 | 34 | 5925 | 2.7877 | 0.0026541 | ZNF28 |
| ENSG00000144644 | 3 | 30767692 | 30936257 | 577 | 41 | 5925 | 2.7813 | 0.0027072 | GADL1 |
| ENSG00000141447 | 18 | 21742008 | 21977844 | 572 | 74 | 5925 | 2.7809 | 0.0027108 | OSBPL1A |
| ENSG00000255112 | 18 | 11851395 | 11854448 | 6 | 4 | 5925 | 2.7741 | 0.0027674 | CHMP1B |
| ENSG00000148604 | 10 | 86004809 | 86019716 | 58 | 14 | 5925 | 2.7693 | 0.0028087 | RGR |
| ENSG00000167658 | 19 | 3976054 | 3985467 | 31 | 10 | 5925 | 2.7616 | 0.0028758 | EEF2 |
| ENSG00000145782 | 5 | 115163893 | 115177555 | 27 | 9 | 5925 | 2.7589 | 0.0028997 | ATG12 |
| ENSG00000138744 | 4 | 76831809 | 76862204 | 136 | 14 | 5925 | 2.7567 | 0.0029197 | NAAA |
| ENSG00000197745 | 11 | 62063754 | 62066536 | 9 | 4 | 5925 | 2.7547 | 0.0029376 | SCGB1D4 |
| ENSG00000205413 | 7 | 92728829 | 92747336 | 70 | 14 | 5925 | 2.7524 | 0.0029583 | SAMD9 |
| ENSG00000142698 | 1 | 34632484 | 34684732 | 191 | 27 | 5925 | 2.7431 | 0.0030429 | C1orf94 |
| ENSG00000057593 | 13 | 113760105 | 113774995 | 25 | 7 | 5925 | 2.7393 | 0.0030786 | F7 |
| ENSG00000103269 | 16 | 725666 | 728268 | 1 | 1 | 5925 | 2.739 | 0.003081 | RHBDL1 |
| ENSG00000154162 | 5 | 21750782 | 22853731 | 2854 | 119 | 5925 | 2.7381 | 0.0030899 | CDH12 |
| ENSG00000213988 | 19 | 20188803 | 20237885 | 134 | 24 | 5925 | 2.7354 | 0.0031148 | ZNF90 |
| ENSG00000167774 | 19 | 8373167 | 8386263 | 41 | 5 | 5925 | 2.735 | 0.0031193 | NDUFA7 |
| ENSG00000171501 | 9 | 125315391 | 125316493 | 13 | 4 | 5925 | 2.7349 | 0.0031198 | OR1N2 |
| ENSG00000185551 | 15 | 96869167 | 96883492 | 14 | 3 | 5925 | 2.7337 | 0.0031311 | NR2F2 |
| ENSG00000267855 | 19 | 8373490 | 8386280 | 40 | 5 | 5925 | 2.7308 | 0.0031591 | NDUFA7 |
| ENSG00000125746 | 19 | 46110252 | 46148887 | 97 | 16 | 5925 | 2.7281 | 0.0031852 | EML2 |
| ENSG00000184361 | 17 | 43331760 | 43339479 | 24 | 5 | 5925 | 2.7249 | 0.0032164 | SPATA32 |
| ENSG00000163872 | 3 | 183415606 | 183530413 | 209 | 18 | 5925 | 2.7233 | 0.0032312 | YEATS2 |
| ENSG00000149485 | 11 | 61567099 | 61596790 | 36 | 5 | 5925 | 2.7086 | 0.0033784 | FADS1 |
| ENSG00000171885 | 18 | 24432002 | 24445782 | 36 | 9 | 5925 | 2.7064 | 0.0034008 | AQP4 |
| ENSG00000029639 | 6 | 155578643 | 155635627 | 236 | 25 | 5925 | 2.7038 | 0.0034276 | TFB1M |
| ENSG00000153446 | 16 | 5094123 | 5116111 | 65 | 18 | 5925 | 2.7001 | 0.0034657 | C16orf89 |
| ENSG00000127324 | 12 | 71518865 | 71835678 | 953 | 58 | 5925 | 2.6985 | 0.0034824 | TSPAN8 |
| ENSG00000167775 | 19 | 8367011 | 8373240 | 37 | 4 | 5925 | 2.6983 | 0.0034848 | CD320 |
| ENSG00000072110 | 14 | 69340860 | 69446157 | 399 | 47 | 5925 | 2.6942 | 0.0035276 | ACTN1 |
| ENSG00000131848 | 19 | 56732681 | 56879752 | 479 | 75 | 5925 | 2.6939 | 0.0035311 | ZSCAN5A |
| ENSG00000149557 | 11 | 125315646 | 125366213 | 115 | 19 | 5925 | 2.6867 | 0.0036084 | FEZ1 |
| ENSG00000161677 | 19 | 51009255 | 51014610 | 11 | 5 | 5925 | 2.6865 | 0.0036104 | JOSD2 |
| ENSG00000197595 | 13 | 113301358 | 113338811 | 126 | 22 | 5925 | 2.6833 | 0.0036447 | C13orf35 |
| ENSG00000181381 | 4 | 169277886 | 169458937 | 735 | 61 | 5925 | 2.6831 | 0.0036467 | DDX60L |
| ENSG00000204188 | 6 | 33551515 | 33556803 | 23 | 9 | 5925 | 2.6806 | 0.003675 | GGNBP1 |
| ENSG00000112782 | 6 | 45868045 | 46048132 | 569 | 61 | 5925 | 2.6765 | 0.0037201 | CLIC5 |
| ENSG00000103266 | 16 | 730224 | 732870 | 4 | 2 | 5925 | 2.6741 | 0.0037466 | STUB1 |
| ENSG00000108018 | 10 | 108333421 | 108924292 | 1682 | 116 | 5925 | 2.6648 | 0.0038511 | SORCS1 |
| ENSG00000162992 | 2 | 182537815 | 182545603 | 8 | 3 | 5925 | 2.6632 | 0.0038702 | NEUROD1 |
| ENSG00000157045 | 16 | 15131710 | 15149921 | 45 | 7 | 5925 | 2.6627 | 0.0038762 | NTAN1 |
| ENSG00000173409 | 1 | 231114727 | 231136341 | 64 | 10 | 5925 | 2.651 | 0.0040125 | ARV1 |
| ENSG00000139734 | 13 | 60239717 | 60738121 | 1253 | 39 | 5925 | 2.6485 | 0.0040422 | DIAPH3 |
| ENSG00000176597 | 3 | 182971032 | 183016292 | 116 | 7 | 5925 | 2.6472 | 0.0040581 | B3GNT5 |
| ENSG00000113761 | 5 | 176449697 | 176508190 | 191 | 15 | 5925 | 2.6467 | 0.0040644 | ZNF346 |
| ENSG00000172508 | 11 | 67182439 | 67193078 | 17 | 6 | 5925 | 2.6431 | 0.0041079 | CARNS1 |
| ENSG00000033011 | 16 | 5083703 | 5137380 | 181 | 40 | 5925 | 2.637 | 0.0041827 | ALG1 |
| ENSG00000185070 | 14 | 85996488 | 86095034 | 340 | 40 | 5925 | 2.6341 | 0.0042179 | FLRT2 |
| ENSG00000100601 | 14 | 78138747 | 78174363 | 144 | 21 | 5925 | 2.6315 | 0.00425 | ALKBH1 |
| ENSG00000157954 | 7 | 5229819 | 5273457 | 163 | 27 | 5925 | 2.6281 | 0.0042931 | WIPI2 |
| ENSG00000175463 | 11 | 67171386 | 67177560 | 15 | 5 | 5925 | 2.6276 | 0.0042996 | TBC1D10C |
| ENSG00000185650 | 14 | 69254377 | 69263190 | 20 | 6 | 5925 | 2.6198 | 0.0043987 | ZFP36L1 |
| ENSG00000107187 | 9 | 139088096 | 139096955 | 23 | 8 | 5925 | 2.6178 | 0.004425 | LHX3 |
| ENSG00000176593 | 19 | 58513429 | 58522600 | 39 | 7 | 5925 | 2.616 | 0.0044488 | CTD-2368P22.1 |
| ENSG00000124227 | 20 | 56793551 | 56803709 | 25 | 7 | 5925 | 2.6035 | 0.0046133 | ANKRD60 |
| ENSG00000214753 | 11 | 62480102 | 62494821 | 22 | 6 | 5925 | 2.6013 | 0.0046438 | HNRNPUL2 |
| ENSG00000131591 | 1 | 1017198 | 1051741 | 113 | 15 | 5925 | 2.6002 | 0.0046584 | C1orf159 |
| ENSG00000241484 | 22 | 45098355 | 45258665 | 1018 | 121 | 5925 | 2.5963 | 0.0047123 | ARHGAP8 |
| ENSG00000125844 | 20 | 17594323 | 17662940 | 161 | 31 | 5925 | 2.5943 | 0.0047392 | RRBP1 |
| ENSG00000065268 | 19 | 983174 | 994569 | 70 | 9 | 5925 | 2.5939 | 0.0047445 | WDR18 |
| ENSG00000154265 | 17 | 67240452 | 67323385 | 181 | 20 | 5925 | 2.5916 | 0.0047764 | ABCA5 |
| ENSG00000169213 | 1 | 52373628 | 52456436 | 165 | 22 | 5925 | 2.5872 | 0.0048385 | RAB3B |
| ENSG00000124813 | 6 | 45295894 | 45632086 | 966 | 74 | 5925 | 2.582 | 0.0049122 | RUNX2 |
| ENSG00000248405 | 22 | 45098113 | 45258586 | 1015 | 119 | 5925 | 2.5792 | 0.0049516 | PRR5-ARHGAP8 |
| ENSG00000187624 | 17 | 260118 | 273510 | 40 | 8 | 5925 | 2.5769 | 0.004984 | C17orf97 |
| ENSG00000159322 | 15 | 73043710 | 73078187 | 72 | 12 | 5925 | 2.5768 | 0.0049866 | ADPGK |
| ENSG00000187642 | 1 | 910579 | 917497 | 24 | 6 | 5925 | 2.5743 | 0.0050218 | C1orf170 |
| ENSG00000248099 | 19 | 17927321 | 17932383 | 16 | 5 | 5925 | 2.5734 | 0.005035 | INSL3 |
| ENSG00000091317 | 3 | 32522804 | 32544900 | 51 | 14 | 5925 | 2.5644 | 0.005167 | CMTM6 |
| ENSG00000198324 | 12 | 111798455 | 111806925 | 13 | 4 | 5925 | 2.563 | 0.0051887 | FAM109A |
| ENSG00000126215 | 14 | 104163946 | 104181841 | 35 | 10 | 5925 | 2.5623 | 0.0051987 | XRCC3 |
| ENSG00000197860 | 5 | 64961755 | 65018862 | 96 | 16 | 5925 | 2.5481 | 0.0054154 | SGTB |
| ENSG00000143248 | 1 | 163080911 | 163291577 | 843 | 53 | 5925 | 2.5465 | 0.0054401 | RGS5 |
| ENSG00000115738 | 2 | 8818975 | 8824583 | 10 | 5 | 5925 | 2.5447 | 0.0054679 | ID2 |
| ENSG00000056586 | 9 | 125606835 | 125667620 | 101 | 16 | 5925 | 2.5352 | 0.0056192 | RC3H2 |
| ENSG00000156222 | 15 | 85427885 | 85518876 | 402 | 53 | 5925 | 2.5329 | 0.0056563 | SLC28A1 |
| ENSG00000091181 | 3 | 3111233 | 3168297 | 283 | 66 | 5925 | 2.5315 | 0.0056794 | IL5RA |
| ENSG00000156795 | 8 | 124428965 | 124479470 | 274 | 8 | 5925 | 2.5299 | 0.0057051 | WDYHV1 |
| ENSG00000156194 | 4 | 76781020 | 76823724 | 85 | 15 | 5925 | 2.529 | 0.0057202 | PPEF2 |
| ENSG00000136485 | 17 | 61627822 | 61671639 | 50 | 16 | 5925 | 2.5262 | 0.0057659 | DCAF7 |
| ENSG00000171858 | 20 | 60962172 | 60963576 | 4 | 2 | 5925 | 2.5219 | 0.0058361 | RPS21 |
| ENSG00000164327 | 5 | 38938021 | 39074510 | 280 | 14 | 5925 | 2.52 | 0.0058675 | RICTOR |
| ENSG00000204293 | 11 | 124252291 | 124253258 | 2 | 1 | 5925 | 2.5175 | 0.0059088 | OR8B2 |
| ENSG00000130270 | 19 | 1782074 | 1812275 | 93 | 23 | 5925 | 2.5172 | 0.0059154 | ATP8B3 |
| ENSG00000136144 | 13 | 50106082 | 50159719 | 267 | 14 | 5925 | 2.5153 | 0.005947 | RCBTB1 |
| ENSG00000188167 | 3 | 33131913 | 33138293 | 6 | 4 | 5925 | 2.514 | 0.005968 | TMPPE |
| ENSG00000183520 | 1 | 38474930 | 38490496 | 33 | 14 | 5925 | 2.5126 | 0.0059915 | UTP11L |
| ENSG00000068784 | 2 | 45615819 | 45839304 | 666 | 31 | 5925 | 2.5124 | 0.005995 | SRBD1 |
| ENSG00000149639 | 20 | 35405845 | 35492089 | 121 | 27 | 5925 | 2.5076 | 0.006078 | SOGA1 |
| ENSG00000100104 | 22 | 26879843 | 26890624 | 25 | 8 | 5925 | 2.5073 | 0.0060828 | SRRD |
| ENSG00000165643 | 9 | 138585253 | 138591374 | 36 | 7 | 5925 | 2.5067 | 0.0060935 | SOHLH1 |
| ENSG00000073331 | 4 | 113206665 | 113363776 | 439 | 62 | 5925 | 2.5045 | 0.0061307 | ALPK1 |
| ENSG00000166503 | 15 | 83784320 | 83876770 | 156 | 13 | 5925 | 2.4996 | 0.006216 | HDGFRP3 |
| ENSG00000131059 | 20 | 31805116 | 31815564 | 12 | 7 | 5925 | 2.4941 | 0.0063134 | BPIFA3 |
| ENSG00000144036 | 2 | 72403113 | 73053170 | 1017 | 30 | 5925 | 2.4897 | 0.0063923 | EXOC6B |
| ENSG00000149930 | 16 | 29984962 | 30003582 | 28 | 5 | 5925 | 2.4867 | 0.0064466 | TAOK2 |
| ENSG00000168143 | 6 | 54711569 | 54806820 | 339 | 24 | 5925 | 2.4858 | 0.0064634 | FAM83B |
| ENSG00000164600 | 7 | 31377075 | 31380508 | 5 | 2 | 5925 | 2.483 | 0.0065143 | NEUROD6 |
| ENSG00000197826 | 4 | 81256874 | 81884910 | 1724 | 42 | 5925 | 2.4798 | 0.0065732 | C4orf22 |
| ENSG00000006282 | 17 | 48620419 | 48633213 | 46 | 8 | 5925 | 2.4769 | 0.0066273 | SPATA20 |
| ENSG00000130479 | 19 | 17830051 | 17845325 | 65 | 9 | 5925 | 2.4745 | 0.0066703 | MAP1S |
| ENSG00000131368 | 3 | 15083967 | 15106842 | 35 | 9 | 5925 | 2.4703 | 0.0067496 | MRPS25 |
| ENSG00000268208 | 14 | 78227711 | 78228033 | 3 | 1 | 5925 | 2.47 | 0.0067554 | AC008372.1 |
| ENSG00000149932 | 16 | 29952206 | 29984373 | 46 | 7 | 5925 | 2.4687 | 0.0067809 | TMEM219 |
| ENSG00000148513 | 10 | 37414785 | 37673039 | 594 | 33 | 5925 | 2.4645 | 0.0068602 | ANKRD30A |
| ENSG00000169282 | 3 | 155755490 | 156256545 | 1389 | 70 | 5925 | 2.4635 | 0.0068794 | KCNAB1 |
| ENSG00000103174 | 16 | 5074845 | 5084142 | 32 | 9 | 5925 | 2.458 | 0.0069853 | NAGPA |
| ENSG00000135090 | 12 | 118587606 | 118810750 | 444 | 25 | 5925 | 2.4526 | 0.0070912 | TAOK3 |
| ENSG00000090006 | 19 | 41098789 | 41135725 | 110 | 15 | 5925 | 2.4514 | 0.0071151 | LTBP4 |
| ENSG00000071054 | 2 | 102313312 | 102511149 | 383 | 31 | 5925 | 2.4507 | 0.0071293 | MAP4K4 |
| ENSG00000196800 | 5 | 147549296 | 147554961 | 8 | 3 | 5925 | 2.4489 | 0.0071655 | SPINK14 |
| ENSG00000153048 | 16 | 8946799 | 8962866 | 101 | 21 | 5925 | 2.4482 | 0.0071792 | CARHSP1 |
| ENSG00000196917 | 12 | 123104824 | 123215390 | 274 | 33 | 5925 | 2.4446 | 0.0072498 | HCAR1 |
| ENSG00000174953 | 3 | 153990335 | 154042286 | 97 | 16 | 5925 | 2.4433 | 0.0072765 | DHX36 |
| ENSG00000175764 | 9 | 124584207 | 124855885 | 992 | 61 | 5925 | 2.4406 | 0.0073309 | TTLL11 |
| ENSG00000175482 | 11 | 67118248 | 67124443 | 10 | 3 | 5925 | 2.4405 | 0.0073343 | POLD4 |
| ENSG00000079393 | 10 | 76854192 | 76868979 | 47 | 11 | 5925 | 2.4385 | 0.007375 | DUSP13 |
| ENSG00000116704 | 1 | 67465015 | 67519782 | 165 | 16 | 5925 | 2.4356 | 0.0074326 | SLC35D1 |
| ENSG00000116691 | 1 | 12079523 | 12092102 | 24 | 6 | 5925 | 2.4334 | 0.0074786 | MIIP |
| ENSG00000102466 | 13 | 102372134 | 103054124 | 1941 | 119 | 5925 | 2.4322 | 0.0075046 | FGF14 |
| ENSG00000184459 | 22 | 32809834 | 32860471 | 225 | 50 | 5925 | 2.4312 | 0.0075252 | BPIFC |
| ENSG00000175505 | 11 | 67131639 | 67141648 | 21 | 6 | 5925 | 2.4239 | 0.0076773 | CLCF1 |
| ENSG00000137497 | 11 | 71713910 | 71791739 | 159 | 10 | 5925 | 2.421 | 0.0077385 | NUMA1 |
| ENSG00000188996 | 6 | 655939 | 656963 | 4 | 2 | 5925 | 2.4208 | 0.0077422 | HUS1B |
| ENSG00000269169 | 19 | 999601 | 999952 | 2 | 1 | 5925 | 2.4187 | 0.0077871 | AC004528.1 |
| ENSG00000198160 | 1 | 67390578 | 67454302 | 139 | 14 | 5925 | 2.4154 | 0.0078591 | MIER1 |
| ENSG00000182004 | 1 | 203830731 | 203839678 | 34 | 6 | 5925 | 2.4148 | 0.0078726 | SNRPE |
| ENSG00000100312 | 22 | 51176624 | 51183762 | 14 | 7 | 5925 | 2.4118 | 0.0079367 | ACR |
| ENSG00000256053 | 14 | 104029299 | 104073860 | 109 | 14 | 5925 | 2.4092 | 0.0079927 | APOPT1 |
| ENSG00000110172 | 11 | 89934328 | 89956532 | 70 | 9 | 5925 | 2.4084 | 0.0080113 | CHORDC1 |
| ENSG00000171217 | 6 | 155585147 | 155597682 | 64 | 10 | 5925 | 2.4082 | 0.0080167 | CLDN20 |
| ENSG00000197548 | 3 | 11313995 | 11599139 | 634 | 23 | 5925 | 2.408 | 0.0080198 | ATG7 |
| ENSG00000164815 | 7 | 103766788 | 103848495 | 251 | 23 | 5925 | 2.4061 | 0.0080621 | ORC5 |
| ENSG00000267710 | 19 | 56784138 | 56821819 | 113 | 19 | 5925 | 2.4059 | 0.0080673 | AC006116.20 |
| ENSG00000120159 | 9 | 26840683 | 26892802 | 128 | 22 | 5925 | 2.3973 | 0.0082572 | CAAP1 |
| ENSG00000100577 | 14 | 77787227 | 77797940 | 40 | 11 | 5925 | 2.397 | 0.0082658 | GSTZ1 |
| ENSG00000070366 | 17 | 1963133 | 2207065 | 637 | 28 | 5925 | 2.3962 | 0.0082823 | SMG6 |
| ENSG00000116288 | 1 | 8014351 | 8045565 | 68 | 13 | 5925 | 2.3926 | 0.0083658 | PARK7 |
| ENSG00000142082 | 11 | 215458 | 236931 | 114 | 12 | 5925 | 2.3924 | 0.0083696 | SIRT3 |
| ENSG00000114757 | 3 | 179512746 | 179754841 | 686 | 48 | 5925 | 2.3888 | 0.0084521 | PEX5L |
| ENSG00000185627 | 11 | 236546 | 252983 | 102 | 14 | 5925 | 2.3865 | 0.0085057 | PSMD13 |
| ENSG00000196526 | 4 | 7760441 | 7941653 | 940 | 43 | 5925 | 2.3855 | 0.0085288 | AFAP1 |
| ENSG00000254469 | 11 | 71576555 | 71639700 | 253 | 7 | 5925 | 2.3832 | 0.0085823 | RP11-849H4.2 |
| ENSG00000165409 | 14 | 81421333 | 81612646 | 733 | 53 | 5925 | 2.3819 | 0.0086109 | TSHR |
| ENSG00000135824 | 1 | 182615239 | 182653711 | 104 | 14 | 5925 | 2.3805 | 0.0086446 | RGS8 |
| ENSG00000147421 | 8 | 28747911 | 28922281 | 221 | 26 | 5925 | 2.3773 | 0.0087207 | HMBOX1 |
| ENSG00000234857 | 11 | 62457747 | 62494856 | 64 | 9 | 5925 | 2.3757 | 0.0087577 | HNRNPUL2-BSCL2 |
| ENSG00000161671 | 19 | 50979657 | 50986608 | 20 | 8 | 5925 | 2.3663 | 0.008984 | EMC10 |
| ENSG00000184154 | 11 | 71791382 | 71821828 | 68 | 7 | 5925 | 2.3625 | 0.0090762 | LRTOMT |
| ENSG00000166704 | 19 | 58488421 | 58514717 | 97 | 10 | 5925 | 2.3621 | 0.0090864 | ZNF606 |
| ENSG00000119431 | 9 | 116135699 | 116139279 | 20 | 7 | 5925 | 2.3618 | 0.0090923 | HDHD3 |
| ENSG00000171053 | 11 | 125616188 | 125619762 | 25 | 8 | 5925 | 2.3609 | 0.0091147 | PATE1 |
| ENSG00000198909 | 17 | 61699775 | 61773663 | 96 | 8 | 5925 | 2.3601 | 0.0091343 | MAP3K3 |
| ENSG00000101439 | 20 | 23608534 | 23619110 | 33 | 3 | 5925 | 2.3597 | 0.0091439 | CST3 |
| ENSG00000197386 | 4 | 3076408 | 3245676 | 363 | 24 | 5925 | 2.3584 | 0.0091766 | HTT |
| ENSG00000149357 | 11 | 71796941 | 71814433 | 39 | 6 | 5925 | 2.3584 | 0.0091779 | LAMTOR1 |
| ENSG00000042088 | 14 | 90421283 | 90511106 | 240 | 17 | 5925 | 2.3572 | 0.009207 | TDP1 |
| ENSG00000159625 | 16 | 57728705 | 57765717 | 74 | 14 | 5925 | 2.3561 | 0.0092339 | CCDC135 |
| ENSG00000114861 | 3 | 71003844 | 71633140 | 1354 | 133 | 5925 | 2.3536 | 0.0092954 | FOXP1 |
| ENSG00000196405 | 14 | 100437786 | 100610573 | 476 | 35 | 5925 | 2.3536 | 0.0092961 | EVL |
| ENSG00000128285 | 22 | 41074754 | 41078818 | 9 | 4 | 5925 | 2.3533 | 0.0093032 | MCHR1 |
| ENSG00000241852 | 8 | 22457114 | 22461663 | 6 | 2 | 5925 | 2.3532 | 0.0093075 | C8orf58 |
| ENSG00000145423 | 4 | 154701744 | 154710272 | 16 | 6 | 5925 | 2.3498 | 0.0093907 | SFRP2 |
| ENSG00000164484 | 6 | 130686879 | 130764208 | 241 | 24 | 5925 | 2.3445 | 0.0095272 | TMEM200A |
| ENSG00000164136 | 4 | 142557752 | 142655140 | 187 | 22 | 5925 | 2.3427 | 0.0095729 | IL15 |
| ENSG00000221883 | 3 | 48955221 | 48956818 | 1 | 1 | 5925 | 2.3406 | 0.009626 | ARIH2OS |
| ENSG00000152147 | 2 | 38978676 | 39012142 | 80 | 13 | 5925 | 2.3405 | 0.0096294 | GEMIN6 |
| ENSG00000143768 | 1 | 226124298 | 226129189 | 6 | 3 | 5925 | 2.3386 | 0.0096787 | LEFTY2 |
| ENSG00000068650 | 13 | 113344643 | 113541482 | 628 | 54 | 5925 | 2.3383 | 0.0096866 | ATP11A |
| ENSG00000173436 | 1 | 19923477 | 19956314 | 76 | 14 | 5925 | 2.3371 | 0.0097163 | MINOS1 |
| ENSG00000172366 | 16 | 691813 | 698474 | 6 | 3 | 5925 | 2.3279 | 0.0099587 | FAM195A |
| ENSG00000136404 | 15 | 83776159 | 83813606 | 47 | 9 | 5925 | 2.3278 | 0.0099626 | TM6SF1 |
| ENSG00000162222 | 11 | 62495541 | 62507765 | 19 | 5 | 5925 | 2.3206 | 0.010154 | TTC9C |
| ENSG00000087301 | 14 | 52897308 | 53019240 | 295 | 18 | 5925 | 2.3197 | 0.010179 | TXNDC16 |
| ENSG00000154655 | 18 | 5954705 | 6415236 | 1231 | 111 | 5925 | 2.319 | 0.010198 | L3MBTL4 |
| ENSG00000253506 | 17 | 59667794 | 59668563 | 4 | 1 | 5925 | 2.3187 | 0.010204 | NACA2 |
| ENSG00000163393 | 1 | 116519119 | 116612675 | 219 | 26 | 5925 | 2.3161 | 0.010276 | SLC22A15 |
| ENSG00000146909 | 7 | 156742417 | 156765876 | 141 | 14 | 5925 | 2.3154 | 0.010295 | NOM1 |
| ENSG00000180354 | 7 | 30174426 | 30202378 | 102 | 19 | 5925 | 2.3115 | 0.010403 | MTURN |
| ENSG00000196361 | 19 | 11562141 | 11591861 | 79 | 14 | 5925 | 2.3113 | 0.010408 | ELAVL3 |
| ENSG00000158457 | 7 | 128784712 | 128808671 | 79 | 9 | 5925 | 2.3102 | 0.010438 | TSPAN33 |
| ENSG00000163960 | 3 | 196074533 | 196159345 | 147 | 22 | 5925 | 2.31 | 0.010443 | UBXN7 |
| ENSG00000197408 | 19 | 41497204 | 41524303 | 120 | 17 | 5925 | 2.3096 | 0.010455 | CYP2B6 |
| ENSG00000181472 | 6 | 151685252 | 151712683 | 60 | 14 | 5925 | 2.3085 | 0.010484 | ZBTB2 |
| ENSG00000105063 | 19 | 55741148 | 55770363 | 63 | 20 | 5925 | 2.3052 | 0.010577 | PPP6R1 |
| ENSG00000124279 | 5 | 7859272 | 7869150 | 35 | 10 | 5925 | 2.3048 | 0.01059 | FASTKD3 |
| ENSG00000161929 | 17 | 5112256 | 5138155 | 82 | 13 | 5925 | 2.3034 | 0.010628 | SCIMP |
| ENSG00000126214 | 14 | 104028233 | 104167888 | 343 | 24 | 5925 | 2.3031 | 0.010636 | KLC1 |
| ENSG00000107807 | 10 | 102889257 | 102897545 | 17 | 6 | 5925 | 2.303 | 0.01064 | TLX1 |
| ENSG00000137522 | 11 | 71639747 | 71708643 | 183 | 10 | 5925 | 2.3019 | 0.010671 | RNF121 |
| ENSG00000168282 | 14 | 50087489 | 50090198 | 2 | 1 | 5925 | 2.2939 | 0.010899 | MGAT2 |
| ENSG00000143891 | 2 | 38893052 | 38968379 | 204 | 28 | 5925 | 2.2922 | 0.010947 | GALM |
| ENSG00000120437 | 6 | 160181360 | 160200144 | 77 | 12 | 5925 | 2.2921 | 0.010951 | ACAT2 |
| ENSG00000064995 | 6 | 34845555 | 34855866 | 23 | 7 | 5925 | 2.2892 | 0.011034 | TAF11 |
| ENSG00000205744 | 19 | 6467218 | 6482568 | 49 | 11 | 5925 | 2.2891 | 0.011035 | DENND1C |
| ENSG00000122711 | 9 | 33218363 | 33248565 | 119 | 13 | 5925 | 2.2881 | 0.011067 | SPINK4 |
| ENSG00000116251 | 1 | 6241329 | 6269449 | 68 | 13 | 5925 | 2.2851 | 0.011154 | RPL22 |
| ENSG00000148386 | 9 | 138555168 | 138558268 | 8 | 5 | 5925 | 2.2843 | 0.011176 | LCN9 |
| ENSG00000224997 | 14 | 104177607 | 104179149 | 3 | 2 | 5925 | 2.2824 | 0.011234 | AL049840.1 |
| ENSG00000125648 | 19 | 6436090 | 6465214 | 109 | 17 | 5925 | 2.2777 | 0.011373 | SLC25A23 |
| ENSG00000135845 | 1 | 172339329 | 172413230 | 169 | 25 | 5925 | 2.2774 | 0.011383 | PIGC |
| ENSG00000186130 | 9 | 125670335 | 125675609 | 11 | 4 | 5925 | 2.2768 | 0.011398 | ZBTB6 |
| ENSG00000162267 | 3 | 52828784 | 52843025 | 28 | 9 | 5925 | 2.2758 | 0.011428 | ITIH3 |
| ENSG00000047617 | 12 | 5641035 | 6055398 | 1437 | 108 | 5925 | 2.2718 | 0.011549 | ANO2 |
| ENSG00000146678 | 7 | 45927956 | 45933267 | 17 | 6 | 5925 | 2.2708 | 0.011579 | IGFBP1 |
| ENSG00000157601 | 21 | 42792231 | 42831141 | 198 | 17 | 5925 | 2.263 | 0.011818 | MX1 |
| ENSG00000179889 | 16 | 15068448 | 15233196 | 185 | 30 | 5925 | 2.2606 | 0.011892 | PDXDC1 |
| ENSG00000269738 | 9 | 125148344 | 125148928 | 2 | 1 | 5925 | 2.2586 | 0.011955 | AL162424.1 |
| ENSG00000168000 | 11 | 62457747 | 62477317 | 37 | 7 | 5925 | 2.255 | 0.012065 | BSCL2 |
| ENSG00000058866 | 3 | 185823457 | 186080026 | 667 | 106 | 5925 | 2.2533 | 0.012119 | DGKG |
| ENSG00000197930 | 14 | 53106634 | 53162618 | 123 | 15 | 5925 | 2.2526 | 0.012141 | ERO1L |
| ENSG00000162585 | 1 | 2115903 | 2144159 | 51 | 11 | 5925 | 2.2523 | 0.012152 | C1orf86 |
| ENSG00000196792 | 14 | 31363005 | 31495607 | 316 | 20 | 5925 | 2.2512 | 0.012186 | STRN3 |
| ENSG00000128585 | 7 | 130794855 | 131181395 | 998 | 101 | 5925 | 2.2493 | 0.012246 | MKLN1 |
| ENSG00000109832 | 11 | 125773271 | 125793158 | 60 | 11 | 5925 | 2.2481 | 0.012284 | DDX25 |
| ENSG00000111249 | 12 | 111471828 | 111788358 | 440 | 39 | 5925 | 2.2476 | 0.0123 | CUX2 |
| ENSG00000159873 | 22 | 29168662 | 29185283 | 50 | 10 | 5925 | 2.2466 | 0.012333 | CCDC117 |
| ENSG00000185899 | 7 | 143140546 | 143141502 | 2 | 1 | 5925 | 2.2438 | 0.012423 | TAS2R60 |
| ENSG00000184857 | 16 | 8874241 | 8891505 | 98 | 16 | 5925 | 2.2406 | 0.012526 | TMEM186 |
| ENSG00000130957 | 9 | 97321002 | 97356075 | 191 | 19 | 5925 | 2.2405 | 0.01253 | FBP2 |
| ENSG00000137038 | 9 | 7796490 | 7888380 | 334 | 39 | 5925 | 2.2381 | 0.012606 | TMEM261 |
| ENSG00000267114 | 19 | 45453301 | 45457264 | 20 | 2 | 5925 | 2.2368 | 0.012649 | CTB-129P6.11 |
| ENSG00000223501 | 6 | 33218049 | 33239824 | 61 | 9 | 5925 | 2.2337 | 0.012752 | VPS52 |
| ENSG00000105996 | 7 | 27139721 | 27142430 | 5 | 2 | 5925 | 2.2331 | 0.01277 | HOXA2 |
| ENSG00000080854 | 11 | 133778459 | 133826880 | 142 | 27 | 5925 | 2.2312 | 0.012833 | IGSF9B |
| ENSG00000111671 | 12 | 6980099 | 6998522 | 37 | 4 | 5925 | 2.2306 | 0.012854 | SPSB2 |
| ENSG00000108465 | 17 | 46045176 | 46059140 | 41 | 8 | 5925 | 2.2306 | 0.012855 | CDK5RAP3 |
| ENSG00000101412 | 20 | 32263489 | 32274210 | 16 | 8 | 5925 | 2.2306 | 0.012855 | E2F1 |
| ENSG00000154229 | 17 | 64298754 | 64806861 | 1799 | 118 | 5925 | 2.2281 | 0.012938 | PRKCA |
| ENSG00000166090 | 14 | 23842018 | 23845612 | 12 | 6 | 5925 | 2.2271 | 0.01297 | IL25 |
| ENSG00000134717 | 1 | 52521797 | 52556388 | 56 | 13 | 5925 | 2.2254 | 0.013027 | BTF3L4 |
| ENSG00000104524 | 8 | 144686083 | 144691943 | 23 | 7 | 5925 | 2.2242 | 0.013069 | PYCRL |
| ENSG00000133028 | 17 | 10583654 | 10601692 | 29 | 4 | 5925 | 2.2235 | 0.01309 | SCO1 |
| ENSG00000088305 | 20 | 31350191 | 31397162 | 138 | 13 | 5925 | 2.223 | 0.013109 | DNMT3B |
| ENSG00000077498 | 11 | 88910620 | 89028927 | 326 | 20 | 5925 | 2.2229 | 0.013112 | TYR |
| ENSG00000083937 | 3 | 87276421 | 87304698 | 79 | 17 | 5925 | 2.2223 | 0.013131 | CHMP2B |
| ENSG00000122507 | 7 | 33168856 | 33645680 | 1460 | 62 | 5925 | 2.2219 | 0.013144 | BBS9 |
| ENSG00000186998 | 22 | 29601840 | 29655586 | 202 | 35 | 5925 | 2.218 | 0.013277 | EMID1 |
| ENSG00000102970 | 16 | 57438679 | 57449974 | 46 | 13 | 5925 | 2.215 | 0.013378 | CCL17 |
| ENSG00000117308 | 1 | 24122089 | 24127271 | 2 | 1 | 5925 | 2.2146 | 0.013392 | GALE |
| ENSG00000163749 | 4 | 77234154 | 77343021 | 200 | 22 | 5925 | 2.2146 | 0.013393 | CCDC158 |
| ENSG00000170324 | 10 | 49364601 | 49482941 | 177 | 18 | 5925 | 2.2142 | 0.013409 | FRMPD2 |
| ENSG00000146648 | 7 | 55086714 | 55324313 | 804 | 92 | 5925 | 2.2133 | 0.013437 | EGFR |
| ENSG00000119509 | 9 | 102861538 | 103063282 | 440 | 17 | 5925 | 2.2113 | 0.013508 | INVS |
| ENSG00000178150 | 19 | 48675575 | 48790865 | 514 | 42 | 5925 | 2.207 | 0.013655 | ZNF114 |
| ENSG00000134825 | 11 | 61535973 | 61560274 | 37 | 9 | 5925 | 2.2055 | 0.013711 | TMEM258 |
| ENSG00000146574 | 7 | 6833765 | 6866401 | 27 | 7 | 5925 | 2.2046 | 0.01374 | CCZ1B |
| ENSG00000162438 | 1 | 15764935 | 15775737 | 25 | 9 | 5925 | 2.2039 | 0.013766 | CTRC |
| ENSG00000167491 | 19 | 19496635 | 19619740 | 220 | 23 | 5925 | 2.2023 | 0.013824 | GATAD2A |
| ENSG00000184163 | 1 | 1177826 | 1182102 | 11 | 2 | 5925 | 2.2016 | 0.013847 | FAM132A |
| ENSG00000184160 | 4 | 3768075 | 3770251 | 3 | 2 | 5925 | 2.2005 | 0.013886 | ADRA2C |
| ENSG00000135898 | 2 | 231772033 | 231825781 | 194 | 38 | 5925 | 2.1996 | 0.013919 | GPR55 |
| ENSG00000164542 | 7 | 36363830 | 36429734 | 175 | 27 | 5925 | 2.1994 | 0.013923 | KIAA0895 |
| ENSG00000129204 | 17 | 5019733 | 5078329 | 127 | 16 | 5925 | 2.1992 | 0.013934 | USP6 |
| ENSG00000180440 | 13 | 37248049 | 37271976 | 64 | 15 | 5925 | 2.1973 | 0.014 | SERTM1 |
| ENSG00000146457 | 6 | 160146617 | 160177351 | 82 | 14 | 5925 | 2.1969 | 0.014015 | WTAP |
| ENSG00000187742 | 9 | 91933421 | 91974557 | 76 | 16 | 5925 | 2.1957 | 0.014058 | SECISBP2 |
| ENSG00000049759 | 18 | 55711599 | 56068772 | 1130 | 98 | 5925 | 2.1954 | 0.014066 | NEDD4L |
| ENSG00000124134 | 20 | 43720951 | 43729753 | 16 | 5 | 5925 | 2.1928 | 0.014159 | KCNS1 |
| ENSG00000133794 | 11 | 13298199 | 13408813 | 294 | 42 | 5925 | 2.1908 | 0.014233 | ARNTL |
| ENSG00000171462 | 6 | 43418090 | 43424370 | 12 | 3 | 5925 | 2.1875 | 0.014352 | DLK2 |
| ENSG00000112667 | 6 | 43193367 | 43197222 | 8 | 4 | 5925 | 2.1871 | 0.014367 | DNPH1 |
| ENSG00000171862 | 10 | 89622870 | 89731687 | 148 | 36 | 5925 | 2.1847 | 0.014456 | PTEN |
| ENSG00000188997 | 11 | 77882295 | 77899868 | 45 | 7 | 5925 | 2.1827 | 0.014527 | KCTD21 |
| ENSG00000145451 | 4 | 175558065 | 175750465 | 732 | 64 | 5925 | 2.1826 | 0.014534 | GLRA3 |
| ENSG00000018699 | 2 | 32853099 | 33046118 | 727 | 46 | 5925 | 2.1825 | 0.014537 | TTC27 |
| ENSG00000100344 | 22 | 44319619 | 44360368 | 175 | 18 | 5925 | 2.182 | 0.014556 | PNPLA3 |
| ENSG00000134504 | 18 | 24034874 | 24237365 | 595 | 87 | 5925 | 2.1809 | 0.014594 | KCTD1 |
| ENSG00000177425 | 12 | 79968759 | 80084877 | 110 | 12 | 5925 | 2.1799 | 0.014631 | PAWR |
| ENSG00000160410 | 19 | 41082757 | 41097305 | 46 | 11 | 5925 | 2.1795 | 0.014649 | SHKBP1 |
| ENSG00000110435 | 11 | 34937376 | 35042138 | 411 | 21 | 5925 | 2.179 | 0.014667 | PDHX |
| ENSG00000110200 | 11 | 71817424 | 71823826 | 11 | 4 | 5925 | 2.1786 | 0.014682 | ANAPC15 |
| ENSG00000162419 | 1 | 28995244 | 29045865 | 71 | 8 | 5925 | 2.1778 | 0.014712 | GMEB1 |
| ENSG00000171914 | 15 | 62682725 | 63136830 | 1307 | 121 | 5925 | 2.1746 | 0.014829 | TLN2 |
| ENSG00000164500 | 7 | 50135632 | 50199426 | 233 | 17 | 5925 | 2.1738 | 0.014859 | C7orf72 |
| ENSG00000185920 | 9 | 98205262 | 98279339 | 170 | 20 | 5925 | 2.1735 | 0.014871 | PTCH1 |
| ENSG00000101413 | 20 | 36661948 | 36720768 | 111 | 9 | 5925 | 2.1696 | 0.015019 | RPRD1B |
| ENSG00000196150 | 8 | 146076632 | 146127553 | 82 | 11 | 5925 | 2.1661 | 0.015153 | ZNF250 |
| ENSG00000180998 | 14 | 53019866 | 53104431 | 197 | 27 | 5925 | 2.165 | 0.015194 | GPR137C |
| ENSG00000077935 | 22 | 45739944 | 45809500 | 186 | 13 | 5925 | 2.1633 | 0.01526 | SMC1B |
| ENSG00000106591 | 7 | 42971799 | 42988557 | 56 | 9 | 5925 | 2.1628 | 0.015277 | MRPL32 |
| ENSG00000111664 | 12 | 6949118 | 6956557 | 10 | 5 | 5925 | 2.1624 | 0.015292 | GNB3 |
| ENSG00000157426 | 4 | 57204456 | 57253666 | 150 | 20 | 5925 | 2.1613 | 0.015338 | AASDH |
| ENSG00000112706 | 6 | 76630832 | 76782395 | 311 | 37 | 5925 | 2.1517 | 0.015709 | IMPG1 |
| ENSG00000197013 | 19 | 21679484 | 21739072 | 169 | 12 | 5925 | 2.1512 | 0.015731 | ZNF429 |
| ENSG00000161992 | 16 | 610422 | 615528 | 19 | 8 | 5925 | 2.15 | 0.015776 | C16orf11 |
| ENSG00000188517 | 4 | 109731877 | 110223813 | 1531 | 88 | 5925 | 2.1499 | 0.015782 | COL25A1 |
| ENSG00000102547 | 13 | 49882786 | 50018262 | 383 | 35 | 5925 | 2.1478 | 0.015863 | CAB39L |
| ENSG00000175130 | 1 | 32799433 | 32801980 | 1 | 1 | 5925 | 2.1437 | 0.01603 | MARCKSL1 |
| ENSG00000131795 | 1 | 145507598 | 145513536 | 2 | 1 | 5925 | 2.1407 | 0.016149 | RBM8A |
| ENSG00000197562 | 16 | 639357 | 679272 | 127 | 11 | 5925 | 2.1402 | 0.016171 | RAB40C |
| ENSG00000145681 | 5 | 82933624 | 83017432 | 252 | 38 | 5925 | 2.1398 | 0.016186 | HAPLN1 |
| ENSG00000130023 | 6 | 170151718 | 170181680 | 99 | 13 | 5925 | 2.1383 | 0.016244 | ERMARD |
| ENSG00000114854 | 3 | 52485118 | 52488086 | 3 | 2 | 5925 | 2.1372 | 0.016292 | TNNC1 |
| ENSG00000184465 | 6 | 169857307 | 170102159 | 766 | 30 | 5925 | 2.1353 | 0.016367 | WDR27 |
| ENSG00000134824 | 11 | 61560452 | 61634826 | 166 | 20 | 5925 | 2.1345 | 0.0164 | FADS2 |
| ENSG00000171496 | 9 | 125329778 | 125330838 | 6 | 2 | 5925 | 2.1333 | 0.01645 | OR1L8 |
| ENSG00000145428 | 4 | 154631277 | 154681387 | 195 | 15 | 5925 | 2.1323 | 0.016492 | RNF175 |
| ENSG00000170464 | 5 | 138744279 | 138780180 | 40 | 6 | 5925 | 2.1309 | 0.016547 | DNAJC18 |
| ENSG00000177596 | 13 | 74987094 | 74993252 | 31 | 11 | 5925 | 2.1302 | 0.016576 | AL355390.1 |
| ENSG00000142615 | 1 | 15783223 | 15798586 | 61 | 11 | 5925 | 2.1293 | 0.016614 | CELA2A |
| ENSG00000161133 | 22 | 20704868 | 20745048 | 72 | 21 | 5925 | 2.1288 | 0.016635 | USP41 |
| ENSG00000116667 | 1 | 184356192 | 184598154 | 604 | 38 | 5925 | 2.1271 | 0.016705 | C1orf21 |
| ENSG00000166170 | 14 | 104022881 | 104029168 | 9 | 2 | 5925 | 2.1266 | 0.016727 | BAG5 |
| ENSG00000136490 | 17 | 61773262 | 61778532 | 5 | 3 | 5925 | 2.1259 | 0.016757 | LIMD2 |
| ENSG00000164691 | 6 | 159455500 | 159466184 | 41 | 6 | 5925 | 2.1233 | 0.016863 | TAGAP |
| ENSG00000177669 | 8 | 29989340 | 30002202 | 34 | 6 | 5925 | 2.1213 | 0.016949 | MBOAT4 |
| ENSG00000174899 | 3 | 157261035 | 157395538 | 456 | 36 | 5925 | 2.121 | 0.01696 | C3orf55 |
| ENSG00000164190 | 5 | 36876861 | 37066515 | 241 | 31 | 5925 | 2.121 | 0.016962 | NIPBL |
| ENSG00000123975 | 9 | 91926113 | 91931618 | 18 | 8 | 5925 | 2.1208 | 0.016969 | CKS2 |
| ENSG00000183844 | 21 | 42676139 | 42729358 | 173 | 28 | 5925 | 2.1207 | 0.016974 | FAM3B |
| ENSG00000181982 | 4 | 24807739 | 24981826 | 522 | 38 | 5925 | 2.1198 | 0.017011 | CCDC149 |
| ENSG00000146414 | 6 | 146185381 | 146285559 | 157 | 11 | 5925 | 2.1185 | 0.017067 | SHPRH |
| ENSG00000108187 | 10 | 70042417 | 70092806 | 178 | 12 | 5925 | 2.1181 | 0.017082 | PBLD |
| ENSG00000133115 | 13 | 39540062 | 39565203 | 90 | 18 | 5925 | 2.1172 | 0.017124 | STOML3 |
| ENSG00000156427 | 5 | 170846660 | 170884627 | 88 | 20 | 5925 | 2.117 | 0.01713 | FGF18 |
| ENSG00000138463 | 3 | 122513642 | 122599986 | 197 | 27 | 5925 | 2.1167 | 0.017143 | DIRC2 |
| ENSG00000116754 | 1 | 70671365 | 70718735 | 59 | 9 | 5925 | 2.1142 | 0.01725 | SRSF11 |
| ENSG00000134030 | 18 | 46065417 | 46389588 | 1120 | 143 | 5925 | 2.1131 | 0.017296 | CTIF |
| ENSG00000178057 | 3 | 49057892 | 49060928 | 1 | 1 | 5925 | 2.1109 | 0.01739 | NDUFAF3 |
| ENSG00000134326 | 2 | 6980701 | 7006766 | 28 | 13 | 5925 | 2.1104 | 0.017412 | CMPK2 |
| ENSG00000268080 | 8 | 94241867 | 94242388 | 1 | 1 | 5925 | 2.1084 | 0.0175 | AC016885.1 |
| ENSG00000106077 | 7 | 73150424 | 73153197 | 6 | 3 | 5925 | 2.1071 | 0.017554 | ABHD11 |
| ENSG00000197448 | 7 | 142941186 | 142967947 | 17 | 10 | 5925 | 2.1067 | 0.017572 | GSTK1 |
| ENSG00000102760 | 13 | 42031695 | 42045018 | 37 | 11 | 5925 | 2.1065 | 0.017579 | RGCC |
| ENSG00000185238 | 11 | 20409076 | 20530840 | 302 | 21 | 5925 | 2.105 | 0.017647 | PRMT3 |
| ENSG00000053524 | 3 | 182895831 | 183146566 | 745 | 52 | 5925 | 2.1049 | 0.01765 | MCF2L2 |
| ENSG00000088247 | 19 | 6413359 | 6424805 | 39 | 5 | 5925 | 2.1047 | 0.017661 | KHSRP |
| ENSG00000156017 | 9 | 77595936 | 77643339 | 91 | 15 | 5925 | 2.1027 | 0.017746 | C9orf41 |
| ENSG00000197555 | 14 | 71787166 | 72207946 | 617 | 37 | 5925 | 2.1001 | 0.01786 | SIPA1L1 |
| ENSG00000064835 | 3 | 87308554 | 87325737 | 37 | 9 | 5925 | 2.0983 | 0.017941 | POU1F1 |
| ENSG00000070785 | 1 | 45316450 | 45452282 | 352 | 14 | 5925 | 2.0982 | 0.017943 | EIF2B3 |
| ENSG00000163171 | 2 | 37869032 | 37965611 | 375 | 46 | 5925 | 2.0915 | 0.018244 | CDC42EP3 |
| ENSG00000120937 | 1 | 11917521 | 11918988 | 1 | 1 | 5925 | 2.0913 | 0.01825 | NPPB |
| ENSG00000173020 | 11 | 67033881 | 67054027 | 22 | 7 | 5925 | 2.0913 | 0.01825 | ADRBK1 |
| ENSG00000112659 | 6 | 43149913 | 43192325 | 95 | 15 | 5925 | 2.0905 | 0.018288 | CUL9 |
| ENSG00000256500 | 14 | 104029299 | 104152261 | 303 | 20 | 5925 | 2.09 | 0.018307 | RP11-73M18.2 |
| ENSG00000120438 | 6 | 160199530 | 160210781 | 32 | 7 | 5925 | 2.0887 | 0.018369 | TCP1 |
| ENSG00000118804 | 4 | 77172886 | 77232752 | 232 | 20 | 5925 | 2.0885 | 0.018376 | FAM47E-STBD1 |
| ENSG00000101574 | 18 | 2537524 | 2571508 | 117 | 12 | 5925 | 2.0867 | 0.018456 | METTL4 |
| ENSG00000106355 | 7 | 32524951 | 32534895 | 42 | 7 | 5925 | 2.0866 | 0.018463 | LSM5 |
| ENSG00000155100 | 8 | 92082424 | 92099323 | 28 | 6 | 5925 | 2.0853 | 0.018519 | OTUD6B |
| ENSG00000237353 | 11 | 125703211 | 125709964 | 34 | 8 | 5925 | 2.0844 | 0.018561 | PATE4 |
| ENSG00000111707 | 12 | 118814185 | 118855840 | 118 | 12 | 5925 | 2.0828 | 0.018636 | SUDS3 |
| ENSG00000004766 | 7 | 92861653 | 92988338 | 180 | 31 | 5925 | 2.0808 | 0.018727 | CCDC132 |
| ENSG00000272414 | 4 | 77172874 | 77232282 | 230 | 20 | 5925 | 2.0803 | 0.018747 | FAM47E-STBD1 |
| ENSG00000129933 | 19 | 19431490 | 19469563 | 87 | 13 | 5925 | 2.0784 | 0.018834 | MAU2 |
| ENSG00000118245 | 2 | 217724181 | 217724787 | 1 | 1 | 5925 | 2.0783 | 0.01884 | TNP1 |
| ENSG00000090447 | 16 | 4307187 | 4323076 | 25 | 5 | 5925 | 2.0773 | 0.018889 | TFAP4 |
| ENSG00000150630 | 4 | 177604689 | 177713881 | 295 | 19 | 5925 | 2.0766 | 0.018917 | VEGFC |
| ENSG00000214900 | 14 | 50448430 | 50474238 | 91 | 16 | 5925 | 2.0763 | 0.018932 | C14orf182 |
| ENSG00000105483 | 19 | 48684027 | 48759203 | 294 | 28 | 5925 | 2.0748 | 0.019003 | CARD8 |
| ENSG00000155530 | 7 | 133812052 | 133949343 | 350 | 14 | 5925 | 2.0745 | 0.019017 | LRGUK |
| ENSG00000130024 | 6 | 170104001 | 170124151 | 35 | 8 | 5925 | 2.0723 | 0.019117 | PHF10 |
| ENSG00000213171 | 1 | 151772740 | 151778630 | 7 | 2 | 5925 | 2.0704 | 0.019205 | LINGO4 |
| ENSG00000100099 | 22 | 26839389 | 26879803 | 133 | 18 | 5925 | 2.0696 | 0.019245 | HPS4 |
| ENSG00000268818 | 22 | 36023040 | 36031181 | 27 | 8 | 5925 | 2.0695 | 0.019248 | AL049747.1 |
| ENSG00000270136 | 1 | 19923473 | 19984549 | 174 | 31 | 5925 | 2.0657 | 0.019426 | MINOS1-NBL1 |
| ENSG00000117543 | 1 | 101455179 | 101491644 | 47 | 7 | 5925 | 2.0647 | 0.019478 | DPH5 |
| ENSG00000170549 | 5 | 3596168 | 3601517 | 14 | 4 | 5925 | 2.0626 | 0.019574 | IRX1 |
| ENSG00000266997 | 18 | 47322163 | 47363993 | 104 | 17 | 5925 | 2.0624 | 0.019584 | RP11-886H22.1 |
| ENSG00000166426 | 15 | 78632666 | 78640572 | 21 | 9 | 5925 | 2.059 | 0.019745 | CRABP1 |
| ENSG00000101335 | 20 | 35169887 | 35178228 | 14 | 8 | 5925 | 2.0573 | 0.019828 | MYL9 |
| ENSG00000165501 | 14 | 50065415 | 50081390 | 48 | 6 | 5925 | 2.0555 | 0.019915 | LRR1 |
| ENSG00000206418 | 18 | 8609443 | 8639379 | 87 | 9 | 5925 | 2.0542 | 0.019978 | RAB12 |
| ENSG00000161999 | 16 | 731671 | 734529 | 5 | 2 | 5925 | 2.0525 | 0.020062 | JMJD8 |
| ENSG00000111537 | 12 | 68548548 | 68553527 | 5 | 2 | 5925 | 2.0513 | 0.020119 | IFNG |
| ENSG00000175445 | 8 | 19759228 | 19824769 | 256 | 20 | 5925 | 2.0509 | 0.020136 | LPL |
| ENSG00000135423 | 12 | 56864736 | 56882198 | 22 | 6 | 5925 | 2.05 | 0.020184 | GLS2 |
| ENSG00000186117 | 11 | 55578854 | 55579952 | 6 | 1 | 5925 | 2.0483 | 0.020265 | OR5L1 |
| ENSG00000123268 | 12 | 51157493 | 51214905 | 120 | 14 | 5925 | 2.0481 | 0.020277 | ATF1 |
| ENSG00000015171 | 10 | 180405 | 300577 | 210 | 23 | 5925 | 2.0473 | 0.020312 | ZMYND11 |
| ENSG00000009844 | 6 | 142468367 | 142545826 | 146 | 11 | 5925 | 2.0421 | 0.02057 | VTA1 |
| ENSG00000066084 | 12 | 50898768 | 51142450 | 435 | 21 | 5925 | 2.0409 | 0.020631 | DIP2B |
| ENSG00000179979 | 4 | 1385340 | 1389780 | 11 | 3 | 5925 | 2.0407 | 0.020641 | CRIPAK |
| ENSG00000145284 | 4 | 83550692 | 83720010 | 813 | 62 | 5925 | 2.0397 | 0.02069 | SCD5 |
| ENSG00000146453 | 6 | 160221298 | 160241736 | 65 | 14 | 5925 | 2.0383 | 0.020759 | PNLDC1 |
| ENSG00000188396 | 1 | 45271585 | 45272957 | 3 | 2 | 5925 | 2.0383 | 0.02076 | TCTEX1D4 |
| ENSG00000211448 | 14 | 80663873 | 80854100 | 370 | 44 | 5925 | 2.038 | 0.020776 | DIO2 |
| ENSG00000140025 | 14 | 90261013 | 90421121 | 491 | 44 | 5925 | 2.0368 | 0.020834 | EFCAB11 |
| ENSG00000172613 | 11 | 67159176 | 67165881 | 15 | 6 | 5925 | 2.0364 | 0.020854 | RAD9A |
| ENSG00000149196 | 11 | 86013253 | 86056969 | 151 | 16 | 5925 | 2.0361 | 0.020869 | C11orf73 |
| ENSG00000168538 | 4 | 184580420 | 184634745 | 199 | 21 | 5925 | 2.0336 | 0.020996 | TRAPPC11 |
| ENSG00000100445 | 14 | 24908972 | 24912111 | 19 | 2 | 5925 | 2.0328 | 0.021039 | SDR39U1 |
| ENSG00000115484 | 2 | 62095224 | 62115939 | 64 | 11 | 5925 | 2.0308 | 0.021139 | CCT4 |
| ENSG00000165215 | 7 | 73183328 | 73184600 | 3 | 1 | 5925 | 2.03 | 0.021179 | CLDN3 |
| ENSG00000168916 | 5 | 123972608 | 124084500 | 321 | 37 | 5925 | 2.0298 | 0.021188 | ZNF608 |
| ENSG00000104853 | 19 | 45457842 | 45496599 | 121 | 18 | 5925 | 2.0298 | 0.021191 | CLPTM1 |
| ENSG00000112238 | 6 | 100054606 | 100063454 | 11 | 5 | 5925 | 2.0294 | 0.021207 | PRDM13 |
| ENSG00000174672 | 11 | 1411129 | 1483919 | 336 | 33 | 5925 | 2.0292 | 0.021217 | BRSK2 |
| ENSG00000165140 | 9 | 97365415 | 97402531 | 134 | 18 | 5925 | 2.0269 | 0.021338 | FBP1 |
| ENSG00000166152 | 16 | 49407734 | 49433342 | 79 | 11 | 5925 | 2.0229 | 0.021541 | C16orf78 |
| ENSG00000188112 | 6 | 42068856 | 42110357 | 164 | 32 | 5925 | 2.02 | 0.021691 | C6orf132 |
| ENSG00000180386 | 17 | 39431911 | 39432949 | 1 | 1 | 5925 | 2.0185 | 0.02177 | KRTAP9-7 |
| ENSG00000179826 | 11 | 18142502 | 18160027 | 39 | 8 | 5925 | 2.0184 | 0.021777 | MRGPRX3 |
| ENSG00000137203 | 6 | 10393419 | 10419892 | 46 | 12 | 5925 | 2.0168 | 0.021859 | TFAP2A |
| ENSG00000144015 | 2 | 96257766 | 96265526 | 3 | 2 | 5925 | 2.016 | 0.021902 | TRIM43 |
| ENSG00000164307 | 5 | 96096521 | 96143803 | 236 | 22 | 5925 | 2.0141 | 0.022001 | ERAP1 |
| ENSG00000170469 | 5 | 138732252 | 138739777 | 8 | 2 | 5925 | 2.0126 | 0.022076 | SPATA24 |
| ENSG00000125772 | 20 | 5525085 | 5591672 | 178 | 23 | 5925 | 2.0123 | 0.022096 | GPCPD1 |
| ENSG00000070367 | 14 | 57670518 | 57735726 | 81 | 12 | 5925 | 2.0122 | 0.022098 | EXOC5 |
| ENSG00000092847 | 1 | 36335409 | 36395211 | 55 | 10 | 5925 | 2.0113 | 0.022148 | AGO1 |
| ENSG00000048828 | 9 | 96214004 | 96328397 | 333 | 21 | 5925 | 2.0105 | 0.02219 | FAM120A |
| ENSG00000152208 | 4 | 93225550 | 94695707 | 3773 | 128 | 5925 | 2.0097 | 0.02223 | GRID2 |
| ENSG00000135773 | 1 | 230883130 | 230937749 | 251 | 32 | 5925 | 2.0095 | 0.022241 | CAPN9 |
| ENSG00000105146 | 19 | 57742377 | 57746916 | 14 | 3 | 5925 | 2.0095 | 0.022243 | AURKC |
| ENSG00000215912 | 1 | 2567415 | 2718286 | 125 | 15 | 5925 | 2.0092 | 0.022259 | TTC34 |
| ENSG00000136824 | 9 | 106856541 | 106903698 | 85 | 8 | 5925 | 2.0075 | 0.022347 | SMC2 |
| ENSG00000111669 | 12 | 6976283 | 6980112 | 6 | 1 | 5925 | 2.0067 | 0.022388 | TPI1 |
| ENSG00000273266 | 12 | 54447661 | 54449814 | 5 | 3 | 5925 | 2.006 | 0.02243 | HOXC4 |
| ENSG00000167757 | 19 | 51525472 | 51531295 | 16 | 8 | 5925 | 2.0053 | 0.022464 | KLK11 |
| ENSG00000123297 | 12 | 58176372 | 58201854 | 39 | 7 | 5925 | 2.0048 | 0.022491 | TSFM |
| ENSG00000149927 | 16 | 30016830 | 30034591 | 34 | 5 | 5925 | 2.004 | 0.022533 | DOC2A |
| ENSG00000111667 | 12 | 6961292 | 6975796 | 12 | 5 | 5925 | 2.0036 | 0.022555 | USP5 |
| ENSG00000105695 | 19 | 35783028 | 35804707 | 56 | 17 | 5925 | 2.0035 | 0.02256 | MAG |
| ENSG00000221888 | 1 | 247920676 | 247921982 | 5 | 2 | 5925 | 2.0032 | 0.022575 | OR1C1 |
| ENSG00000166106 | 11 | 130318869 | 130346532 | 69 | 17 | 5925 | 2.0018 | 0.022651 | ADAMTS15 |
| ENSG00000159263 | 21 | 38071433 | 38122218 | 158 | 42 | 5925 | 2.0014 | 0.022672 | SIM2 |
| ENSG00000262664 | 17 | 1945327 | 1946724 | 6 | 2 | 5925 | 2.0012 | 0.022686 | OVCA2 |
| ENSG00000064309 | 11 | 125825691 | 125933230 | 363 | 40 | 5925 | 2.0003 | 0.022732 | CDON |
| ENSG00000135045 | 9 | 77561497 | 77567802 | 13 | 6 | 5925 | 2.0001 | 0.022743 | C9orf40 |
| ENSG00000108468 | 17 | 46147414 | 46178883 | 91 | 13 | 5925 | 1.9998 | 0.02276 | CBX1 |
| ENSG00000100219 | 22 | 29190543 | 29196585 | 8 | 4 | 5925 | 1.9989 | 0.022809 | XBP1 |
| ENSG00000179038 | 11 | 66963391 | 66964638 | 2 | 1 | 5925 | 1.9983 | 0.022844 | AP001885.1 |
| ENSG00000196968 | 10 | 75532049 | 75540009 | 10 | 2 | 5925 | 1.9973 | 0.022899 | FUT11 |
| ENSG00000116285 | 1 | 8064464 | 8086368 | 35 | 8 | 5925 | 1.9968 | 0.022922 | ERRFI1 |
| ENSG00000181240 | 19 | 6426048 | 6433790 | 22 | 6 | 5925 | 1.9962 | 0.022956 | SLC25A41 |
| ENSG00000241595 | 17 | 39405939 | 39406904 | 3 | 1 | 5925 | 1.9946 | 0.023043 | KRTAP9-4 |
| ENSG00000245848 | 19 | 33790840 | 33793470 | 5 | 1 | 5925 | 1.994 | 0.023079 | CEBPA |
| ENSG00000129596 | 5 | 115140430 | 115152651 | 12 | 7 | 5925 | 1.9934 | 0.023112 | CDO1 |
| ENSG00000072182 | 2 | 220378892 | 220403494 | 54 | 11 | 5925 | 1.9931 | 0.023124 | ASIC4 |
| ENSG00000241123 | 21 | 45999332 | 46000481 | 9 | 3 | 5925 | 1.993 | 0.023133 | KRTAP10-5 |
| ENSG00000141720 | 17 | 36921942 | 36956379 | 78 | 17 | 5925 | 1.9923 | 0.023169 | PIP4K2B |
| ENSG00000115561 | 2 | 86730554 | 86948245 | 451 | 39 | 5925 | 1.9921 | 0.023182 | CHMP3 |
| ENSG00000165646 | 10 | 119000604 | 119038941 | 113 | 25 | 5925 | 1.9916 | 0.023209 | SLC18A2 |
| ENSG00000091947 | 17 | 42088556 | 42101314 | 29 | 8 | 5925 | 1.9913 | 0.023226 | TMEM101 |
| ENSG00000187479 | 11 | 43946892 | 43965888 | 80 | 24 | 5925 | 1.9912 | 0.02323 | C11orf96 |
| ENSG00000170482 | 5 | 138702885 | 138720242 | 22 | 6 | 5925 | 1.9901 | 0.023291 | SLC23A1 |
| ENSG00000154188 | 8 | 108261721 | 108510283 | 831 | 55 | 5925 | 1.9891 | 0.023348 | ANGPT1 |
| ENSG00000198083 | 17 | 39411636 | 39412616 | 1 | 1 | 5925 | 1.9879 | 0.02341 | KRTAP9-9 |
| ENSG00000187258 | 7 | 34697851 | 34917944 | 781 | 50 | 5925 | 1.9875 | 0.023432 | NPSR1 |
| ENSG00000231500 | 6 | 33239787 | 33244287 | 19 | 5 | 5925 | 1.9872 | 0.023448 | RPS18 |
| ENSG00000141349 | 17 | 42148103 | 42153709 | 10 | 5 | 5925 | 1.9866 | 0.023484 | G6PC3 |
| ENSG00000197724 | 9 | 96338689 | 96441869 | 444 | 29 | 5925 | 1.9852 | 0.023564 | PHF2 |
| ENSG00000249884 | 2 | 86732791 | 86948245 | 446 | 37 | 5925 | 1.9845 | 0.023597 | RNF103-CHMP3 |
| ENSG00000167632 | 8 | 140742586 | 141468678 | 2309 | 182 | 5925 | 1.9842 | 0.023615 | TRAPPC9 |
| ENSG00000189143 | 7 | 73213872 | 73247014 | 74 | 7 | 5925 | 1.9833 | 0.023668 | CLDN4 |
| ENSG00000206483 | 3 | 126290625 | 126327398 | 107 | 20 | 5925 | 1.9831 | 0.023678 | TXNRD3NB |
| ENSG00000181264 | 11 | 120195838 | 120204391 | 16 | 4 | 5925 | 1.9796 | 0.023873 | TMEM136 |
| ENSG00000153012 | 4 | 25000469 | 25032501 | 147 | 24 | 5925 | 1.9782 | 0.023952 | LGI2 |
| ENSG00000171448 | 9 | 125677845 | 125693779 | 33 | 10 | 5925 | 1.9753 | 0.024117 | ZBTB26 |
| ENSG00000250733 | 8 | 140943416 | 140946190 | 9 | 4 | 5925 | 1.9736 | 0.024213 | C8orf17 |
| ENSG00000111737 | 12 | 120532899 | 120555306 | 67 | 14 | 5925 | 1.9728 | 0.024259 | RAB35 |
| ENSG00000124275 | 5 | 7851299 | 7906138 | 267 | 29 | 5925 | 1.9693 | 0.024459 | MTRR |
| ENSG00000214841 | 7 | 34685709 | 34699772 | 65 | 13 | 5925 | 1.969 | 0.024474 | AC005493.1 |
| ENSG00000179348 | 3 | 128198270 | 128212028 | 42 | 14 | 5925 | 1.9687 | 0.024493 | GATA2 |
| ENSG00000142959 | 1 | 45249257 | 45253377 | 2 | 1 | 5925 | 1.9669 | 0.024595 | BEST4 |
| ENSG00000187003 | 9 | 111624603 | 111626035 | 3 | 2 | 5925 | 1.9659 | 0.024657 | ACTL7A |
| ENSG00000181562 | 14 | 21214051 | 21216539 | 16 | 4 | 5925 | 1.9639 | 0.024771 | EDDM3A |
| ENSG00000141582 | 17 | 77806955 | 77813228 | 10 | 4 | 5925 | 1.9636 | 0.024787 | CBX4 |
| ENSG00000112110 | 6 | 160210844 | 160219468 | 49 | 7 | 5925 | 1.9628 | 0.024833 | MRPL18 |
| ENSG00000112116 | 6 | 52101479 | 52109335 | 25 | 8 | 5925 | 1.9626 | 0.024844 | IL17F |
| ENSG00000213121 | 6 | 153552455 | 153668623 | 414 | 32 | 5925 | 1.9612 | 0.024929 | AL590867.1 |
| ENSG00000234906 | 19 | 45449243 | 45452822 | 15 | 3 | 5925 | 1.96 | 0.024999 | APOC2 |
| ENSG00000139083 | 12 | 11802788 | 12048336 | 686 | 97 | 5925 | 1.9595 | 0.025028 | ETV6 |
| ENSG00000180251 | 2 | 103089762 | 103150431 | 251 | 18 | 5925 | 1.9592 | 0.025044 | SLC9A4 |
| ENSG00000171940 | 20 | 52183604 | 52226446 | 94 | 27 | 5925 | 1.9581 | 0.025111 | ZNF217 |
| ENSG00000186166 | 11 | 118868852 | 118886501 | 46 | 6 | 5925 | 1.9564 | 0.025211 | CCDC84 |
| ENSG00000179593 | 17 | 7942335 | 7952452 | 53 | 13 | 5925 | 1.9555 | 0.025262 | ALOX15B |
| ENSG00000242802 | 7 | 4815253 | 4833943 | 105 | 11 | 5925 | 1.9554 | 0.025266 | AP5Z1 |
| ENSG00000127863 | 13 | 24144509 | 24250232 | 373 | 41 | 5925 | 1.9554 | 0.025268 | TNFRSF19 |
| ENSG00000168961 | 17 | 25956824 | 25976586 | 47 | 5 | 5925 | 1.9545 | 0.025319 | LGALS9 |
| ENSG00000160753 | 1 | 155290687 | 155300905 | 4 | 2 | 5925 | 1.9527 | 0.02543 | RUSC1 |
| ENSG00000169594 | 15 | 83924655 | 83953466 | 43 | 9 | 5925 | 1.9524 | 0.025444 | BNC1 |
| ENSG00000134780 | 11 | 61447905 | 61514473 | 135 | 21 | 5925 | 1.951 | 0.02553 | DAGLA |
| ENSG00000069702 | 1 | 92145902 | 92371892 | 736 | 65 | 5925 | 1.9477 | 0.025726 | TGFBR3 |
| ENSG00000118997 | 2 | 196602427 | 196933536 | 686 | 32 | 5925 | 1.9472 | 0.025758 | DNAH7 |
| ENSG00000111665 | 12 | 6953957 | 6961230 | 8 | 4 | 5925 | 1.9464 | 0.025806 | CDCA3 |
| ENSG00000133597 | 7 | 140372953 | 140396061 | 38 | 6 | 5925 | 1.9453 | 0.025867 | ADCK2 |
| ENSG00000093072 | 22 | 17660194 | 17702879 | 198 | 42 | 5925 | 1.9444 | 0.025922 | CECR1 |
| ENSG00000065135 | 1 | 110091233 | 110136975 | 71 | 14 | 5925 | 1.9434 | 0.025981 | GNAI3 |
| ENSG00000101407 | 20 | 36611409 | 36661870 | 67 | 14 | 5925 | 1.9377 | 0.026328 | TTI1 |
| ENSG00000106952 | 9 | 117656003 | 117692697 | 98 | 8 | 5925 | 1.937 | 0.026372 | TNFSF8 |
| ENSG00000108666 | 17 | 30651584 | 30677042 | 29 | 8 | 5925 | 1.9354 | 0.02647 | C17orf75 |
| ENSG00000203326 | 19 | 53868946 | 53895470 | 118 | 18 | 5925 | 1.9347 | 0.026516 | ZNF525 |
| ENSG00000204130 | 10 | 70100864 | 70167051 | 139 | 11 | 5925 | 1.9342 | 0.026546 | RUFY2 |
| ENSG00000141449 | 18 | 18822203 | 19105378 | 327 | 25 | 5925 | 1.9339 | 0.02656 | GREB1L |
| ENSG00000213585 | 5 | 133307606 | 133340824 | 83 | 14 | 5925 | 1.9335 | 0.026585 | VDAC1 |
| ENSG00000239886 | 17 | 39382900 | 39383904 | 1 | 1 | 5925 | 1.9331 | 0.02661 | KRTAP9-2 |
| ENSG00000212928 | 14 | 57671888 | 57673291 | 4 | 1 | 5925 | 1.93 | 0.026802 | AL391152.1 |
| ENSG00000197502 | 14 | 50300810 | 50311552 | 15 | 5 | 5925 | 1.9277 | 0.026947 | AL627171.1 |
| ENSG00000163431 | 1 | 201865580 | 201915715 | 121 | 23 | 5925 | 1.9258 | 0.027062 | LMOD1 |
| ENSG00000100079 | 22 | 37966255 | 37978623 | 64 | 11 | 5925 | 1.9247 | 0.027135 | LGALS2 |
| ENSG00000149305 | 11 | 113775399 | 113817287 | 136 | 21 | 5925 | 1.9243 | 0.02716 | HTR3B |
| ENSG00000104613 | 8 | 19674651 | 19709594 | 97 | 17 | 5925 | 1.9239 | 0.027184 | INTS10 |
| ENSG00000131409 | 19 | 51020149 | 51071302 | 131 | 35 | 5925 | 1.9238 | 0.027187 | LRRC4B |
| ENSG00000144591 | 2 | 220363589 | 220371710 | 12 | 5 | 5925 | 1.9236 | 0.027199 | GMPPA |
| ENSG00000170807 | 7 | 123295861 | 123304344 | 22 | 7 | 5925 | 1.9235 | 0.02721 | LMOD2 |
| ENSG00000169208 | 14 | 22037934 | 22038875 | 10 | 6 | 5925 | 1.923 | 0.027239 | OR10G3 |
| ENSG00000010626 | 12 | 6982733 | 7023407 | 93 | 4 | 5925 | 1.9224 | 0.027276 | LRRC23 |
| ENSG00000112787 | 12 | 133066137 | 133161774 | 393 | 34 | 5925 | 1.92 | 0.02743 | FBRSL1 |
| ENSG00000225968 | 7 | 1727755 | 1787590 | 144 | 22 | 5925 | 1.9194 | 0.027466 | ELFN1 |
| ENSG00000118181 | 11 | 118886422 | 118889401 | 15 | 3 | 5925 | 1.9191 | 0.027488 | RPS25 |
| ENSG00000118640 | 2 | 85788685 | 85809154 | 68 | 10 | 5925 | 1.9187 | 0.027513 | VAMP8 |
| ENSG00000177963 | 11 | 207511 | 215113 | 23 | 8 | 5925 | 1.9183 | 0.027537 | RIC8A |
| ENSG00000142583 | 1 | 9095166 | 9148537 | 187 | 29 | 5925 | 1.9182 | 0.027542 | SLC2A5 |
| ENSG00000198203 | 2 | 108905095 | 108926371 | 60 | 13 | 5925 | 1.9171 | 0.02761 | SULT1C2 |
| ENSG00000164756 | 8 | 117962512 | 118188953 | 427 | 31 | 5925 | 1.917 | 0.027616 | SLC30A8 |
| ENSG00000225614 | 16 | 88493879 | 88507165 | 43 | 18 | 5925 | 1.9154 | 0.027719 | ZNF469 |
| ENSG00000165502 | 14 | 50085237 | 50087403 | 7 | 2 | 5925 | 1.9144 | 0.027787 | RPL36AL |
| ENSG00000184029 | 21 | 39323728 | 39493454 | 426 | 51 | 5925 | 1.9129 | 0.027878 | DSCR4 |
| ENSG00000140835 | 16 | 71559136 | 71572649 | 17 | 8 | 5925 | 1.9127 | 0.027891 | CHST4 |
| ENSG00000165548 | 14 | 77582911 | 77725838 | 557 | 44 | 5925 | 1.9119 | 0.027942 | TMEM63C |
| ENSG00000113966 | 3 | 97483365 | 97519953 | 79 | 12 | 5925 | 1.9087 | 0.028148 | ARL6 |
| ENSG00000138138 | 10 | 89511269 | 89601100 | 176 | 15 | 5925 | 1.9083 | 0.028176 | ATAD1 |
| ENSG00000257921 | 12 | 58166811 | 58180829 | 25 | 3 | 5925 | 1.907 | 0.028261 | RP11-571M6.15 |
| ENSG00000215114 | 8 | 59323823 | 59364060 | 93 | 15 | 5925 | 1.9068 | 0.028276 | UBXN2B |
| ENSG00000197763 | 3 | 126290622 | 126373998 | 245 | 30 | 5925 | 1.9036 | 0.028479 | TXNRD3 |
| ENSG00000179918 | 16 | 30454952 | 30457502 | 3 | 2 | 5925 | 1.9033 | 0.028504 | SEPHS2 |
| ENSG00000173120 | 11 | 66886740 | 67025558 | 167 | 25 | 5925 | 1.9013 | 0.028634 | KDM2A |
| ENSG00000128731 | 15 | 28356186 | 28567298 | 192 | 14 | 5925 | 1.9007 | 0.028669 | HERC2 |
| ENSG00000243943 | 2 | 27805897 | 27858041 | 112 | 12 | 5925 | 1.8992 | 0.028767 | ZNF512 |
| ENSG00000091664 | 11 | 22359643 | 22401049 | 128 | 32 | 5925 | 1.8984 | 0.028819 | SLC17A6 |
| ENSG00000140577 | 15 | 91073157 | 91188577 | 336 | 22 | 5925 | 1.8972 | 0.028899 | CRTC3 |
| ENSG00000173124 | 10 | 96953957 | 96988685 | 95 | 7 | 5925 | 1.897 | 0.028913 | C10orf129 |
| ENSG00000188282 | 2 | 218899683 | 218955304 | 143 | 18 | 5925 | 1.8962 | 0.028968 | RUFY4 |
| ENSG00000163357 | 1 | 155006300 | 155023406 | 27 | 7 | 5925 | 1.8959 | 0.028988 | DCST1 |
| ENSG00000162374 | 1 | 50513686 | 50669458 | 204 | 37 | 5925 | 1.8958 | 0.028993 | ELAVL4 |
| ENSG00000173137 | 8 | 145596790 | 145618457 | 29 | 7 | 5925 | 1.8954 | 0.029017 | ADCK5 |
| ENSG00000135437 | 12 | 56114151 | 56118489 | 10 | 4 | 5925 | 1.8952 | 0.029034 | RDH5 |
| ENSG00000090061 | 14 | 99947506 | 100001381 | 100 | 17 | 5925 | 1.8949 | 0.029051 | CCNK |
| ENSG00000204640 | 2 | 101086944 | 101099742 | 58 | 6 | 5925 | 1.8947 | 0.029066 | NMS |
| ENSG00000250091 | 12 | 124410971 | 124419531 | 15 | 3 | 5925 | 1.8946 | 0.029071 | DNAH10OS |
| ENSG00000100335 | 22 | 39895437 | 39914137 | 30 | 8 | 5925 | 1.8944 | 0.029086 | MIEF1 |
| ENSG00000160883 | 5 | 176307870 | 176326333 | 59 | 20 | 5925 | 1.8911 | 0.029303 | HK3 |
| ENSG00000033122 | 1 | 70034081 | 70617628 | 1591 | 77 | 5925 | 1.8911 | 0.029304 | LRRC7 |
| ENSG00000080845 | 20 | 34894258 | 35157040 | 275 | 37 | 5925 | 1.891 | 0.029313 | DLGAP4 |
| ENSG00000127616 | 19 | 11071598 | 11176071 | 291 | 25 | 5925 | 1.8884 | 0.029485 | SMARCA4 |
| ENSG00000100422 | 22 | 47080308 | 47134158 | 209 | 39 | 5925 | 1.885 | 0.029718 | CERK |
| ENSG00000196944 | 1 | 248524883 | 248525929 | 3 | 1 | 5925 | 1.8843 | 0.02976 | OR2T4 |
| ENSG00000143140 | 1 | 147228332 | 147245484 | 67 | 12 | 5925 | 1.8839 | 0.02979 | GJA5 |
| ENSG00000222009 | 1 | 45274154 | 45281257 | 13 | 3 | 5925 | 1.8838 | 0.029794 | BTBD19 |
| ENSG00000179674 | 3 | 160394948 | 160396233 | 2 | 1 | 5925 | 1.8834 | 0.029826 | ARL14 |
| ENSG00000150048 | 12 | 10222153 | 10264226 | 154 | 9 | 5925 | 1.8833 | 0.029828 | CLEC1A |
| ENSG00000258311 | 12 | 56109820 | 56118487 | 15 | 6 | 5925 | 1.8825 | 0.029882 | RP11-644F5.10 |
| ENSG00000163214 | 2 | 39024871 | 39103075 | 170 | 16 | 5925 | 1.882 | 0.029919 | DHX57 |
| ENSG00000172209 | 7 | 107110463 | 107116098 | 7 | 3 | 5925 | 1.8814 | 0.029961 | GPR22 |
| ENSG00000118971 | 12 | 4382938 | 4414516 | 66 | 19 | 5925 | 1.881 | 0.029989 | CCND2 |
| ENSG00000106003 | 7 | 2552163 | 2568811 | 41 | 14 | 5925 | 1.8784 | 0.030166 | LFNG |
| ENSG00000233863 | 8 | 35649365 | 35653384 | 2 | 1 | 5925 | 1.8777 | 0.030211 | AC012215.1 |
| ENSG00000185652 | 12 | 5541278 | 5630702 | 264 | 40 | 5925 | 1.877 | 0.030258 | NTF3 |
| ENSG00000184389 | 1 | 33772367 | 33786699 | 34 | 10 | 5925 | 1.8753 | 0.030378 | A3GALT2 |
| ENSG00000100441 | 14 | 24898492 | 24910540 | 66 | 12 | 5925 | 1.8751 | 0.030391 | KHNYN |
| ENSG00000110344 | 11 | 118230300 | 118269926 | 65 | 13 | 5925 | 1.8744 | 0.030435 | UBE4A |
| ENSG00000270672 | 7 | 142374104 | 142375550 | 7 | 2 | 5925 | 1.8729 | 0.030544 | MTRNR2L6 |
| ENSG00000123427 | 12 | 58165275 | 58176324 | 15 | 2 | 5925 | 1.8728 | 0.030547 | METTL21B |
| ENSG00000256660 | 12 | 10163226 | 10171218 | 20 | 7 | 5925 | 1.8712 | 0.030661 | CLEC12B |
| ENSG00000030110 | 6 | 33540329 | 33548019 | 24 | 7 | 5925 | 1.8703 | 0.030722 | BAK1 |
| ENSG00000089159 | 12 | 120648250 | 120703574 | 80 | 13 | 5925 | 1.87 | 0.030741 | PXN |
| ENSG00000161905 | 17 | 4534197 | 4545589 | 41 | 12 | 5925 | 1.8692 | 0.030797 | ALOX15 |
| ENSG00000090266 | 7 | 140390577 | 140422590 | 47 | 7 | 5925 | 1.869 | 0.030813 | NDUFB2 |
| ENSG00000168746 | 20 | 43080624 | 43093984 | 23 | 8 | 5925 | 1.8671 | 0.030942 | C20orf62 |
| ENSG00000091527 | 3 | 133292574 | 133309105 | 41 | 7 | 5925 | 1.8661 | 0.031017 | CDV3 |
| ENSG00000198648 | 2 | 168810530 | 169104651 | 979 | 49 | 5925 | 1.8658 | 0.031037 | STK39 |
| ENSG00000036530 | 14 | 100150641 | 100193638 | 117 | 16 | 5925 | 1.8657 | 0.031044 | CYP46A1 |
| ENSG00000171794 | 10 | 135043778 | 135045062 | 5 | 2 | 5925 | 1.864 | 0.031163 | UTF1 |
| ENSG00000204446 | 9 | 89763559 | 89774471 | 40 | 7 | 5925 | 1.8632 | 0.031215 | C9orf170 |
| ENSG00000137216 | 6 | 44094651 | 44123256 | 107 | 18 | 5925 | 1.8631 | 0.031227 | TMEM63B |
| ENSG00000168675 | 18 | 13217497 | 13652754 | 1267 | 150 | 5925 | 1.8624 | 0.03127 | LDLRAD4 |
| ENSG00000117036 | 1 | 157090983 | 157108266 | 18 | 10 | 5925 | 1.8619 | 0.031308 | ETV3 |
| ENSG00000172650 | 10 | 75434033 | 75457639 | 6 | 2 | 5925 | 1.8616 | 0.031327 | AGAP5 |
| ENSG00000187051 | 22 | 39925098 | 39928860 | 3 | 1 | 5925 | 1.8611 | 0.031366 | RPS19BP1 |
| ENSG00000161203 | 3 | 183892477 | 183901879 | 23 | 8 | 5925 | 1.861 | 0.031376 | AP2M1 |
| ENSG00000196655 | 11 | 118889142 | 118896164 | 31 | 5 | 5925 | 1.8604 | 0.031416 | TRAPPC4 |
| ENSG00000105717 | 19 | 19672522 | 19729725 | 85 | 15 | 5925 | 1.8598 | 0.031459 | PBX4 |
| ENSG00000244291 | 7 | 156431057 | 156433348 | 5 | 2 | 5925 | 1.859 | 0.031513 | C7orf13 |
| ENSG00000175170 | 20 | 25744102 | 25848861 | 64 | 10 | 5925 | 1.8587 | 0.031533 | FAM182B |
| ENSG00000198104 | 1 | 248550910 | 248551836 | 5 | 1 | 5925 | 1.8573 | 0.031636 | OR2T6 |
| ENSG00000198758 | 1 | 110292702 | 110306649 | 46 | 15 | 5925 | 1.8557 | 0.03175 | EPS8L3 |
| ENSG00000186844 | 1 | 152799949 | 152800573 | 1 | 1 | 5925 | 1.8532 | 0.03193 | LCE1A |
| ENSG00000120837 | 12 | 104510855 | 104532067 | 43 | 12 | 5925 | 1.852 | 0.032012 | NFYB |
| ENSG00000102921 | 16 | 48572637 | 48654059 | 156 | 9 | 5925 | 1.8518 | 0.032029 | N4BP1 |
| ENSG00000259060 | 14 | 21051051 | 21077954 | 119 | 31 | 5925 | 1.8514 | 0.032053 | RNASE11 |
| ENSG00000173464 | 14 | 21051054 | 21078043 | 119 | 31 | 5925 | 1.8514 | 0.032053 | RNASE11 |
| ENSG00000198963 | 9 | 77112281 | 77308093 | 468 | 44 | 5925 | 1.8511 | 0.032074 | RORB |
| ENSG00000171612 | 1 | 9599541 | 9645237 | 62 | 10 | 5925 | 1.8505 | 0.03212 | SLC25A33 |
| ENSG00000111181 | 12 | 299243 | 323736 | 96 | 18 | 5925 | 1.8503 | 0.032135 | SLC6A12 |
| ENSG00000102962 | 16 | 57392684 | 57400102 | 31 | 9 | 5925 | 1.8493 | 0.032206 | CCL22 |
| ENSG00000165553 | 14 | 77731826 | 77737655 | 11 | 6 | 5925 | 1.8488 | 0.032244 | NGB |
| ENSG00000111231 | 12 | 110890289 | 110907073 | 41 | 9 | 5925 | 1.8482 | 0.03229 | GPN3 |
| ENSG00000228835 | 18 | 30349758 | 30354376 | 7 | 3 | 5925 | 1.8468 | 0.032391 | AC012123.1 |
| ENSG00000064726 | 15 | 83685174 | 83736106 | 132 | 21 | 5925 | 1.8466 | 0.032403 | BTBD1 |
| ENSG00000188306 | 3 | 169539710 | 169555563 | 46 | 11 | 5925 | 1.8465 | 0.032412 | LRRIQ4 |
| ENSG00000169507 | 2 | 165752696 | 165812035 | 180 | 21 | 5925 | 1.8459 | 0.032455 | SLC38A11 |
| ENSG00000137834 | 15 | 66994566 | 67074338 | 239 | 36 | 5925 | 1.8444 | 0.032565 | SMAD6 |
| ENSG00000187170 | 1 | 152680636 | 152681967 | 4 | 2 | 5925 | 1.844 | 0.03259 | LCE4A |
| ENSG00000002933 | 7 | 150497491 | 150502208 | 32 | 7 | 5925 | 1.8438 | 0.032607 | TMEM176A |
| ENSG00000177363 | 11 | 62453874 | 62457371 | 3 | 1 | 5925 | 1.8428 | 0.032682 | LRRN4CL |
| ENSG00000002919 | 17 | 46180719 | 46200436 | 65 | 10 | 5925 | 1.8411 | 0.032804 | SNX11 |
| ENSG00000132740 | 11 | 68671310 | 68708070 | 117 | 16 | 5925 | 1.8411 | 0.032805 | IGHMBP2 |
| ENSG00000204524 | 19 | 57751973 | 57766503 | 61 | 7 | 5925 | 1.8408 | 0.032826 | ZNF805 |
| ENSG00000164967 | 9 | 34610483 | 34612101 | 2 | 1 | 5925 | 1.84 | 0.032882 | RPP25L |
| ENSG00000124920 | 11 | 61520114 | 61555990 | 64 | 14 | 5925 | 1.8383 | 0.033009 | MYRF |
| ENSG00000180957 | 22 | 28247657 | 28316122 | 137 | 19 | 5925 | 1.8365 | 0.033144 | PITPNB |
| ENSG00000153015 | 5 | 64064757 | 64314590 | 483 | 25 | 5925 | 1.8363 | 0.033154 | CWC27 |
| ENSG00000196284 | 6 | 44777054 | 45345690 | 1774 | 32 | 5925 | 1.8355 | 0.033214 | SUPT3H |
| ENSG00000186432 | 3 | 160212783 | 160283376 | 129 | 18 | 5925 | 1.8353 | 0.033227 | KPNA4 |
| ENSG00000171502 | 1 | 86194916 | 86622626 | 1472 | 66 | 5925 | 1.8352 | 0.033241 | COL24A1 |
| ENSG00000162777 | 1 | 111729796 | 111747157 | 47 | 17 | 5925 | 1.8326 | 0.033433 | DENND2D |
| ENSG00000128268 | 22 | 39853349 | 39888199 | 60 | 18 | 5925 | 1.829 | 0.0337 | MGAT3 |
| ENSG00000135336 | 6 | 88299839 | 88377169 | 195 | 20 | 5925 | 1.8289 | 0.033707 | ORC3 |
| ENSG00000121741 | 13 | 20532810 | 20665968 | 258 | 17 | 5925 | 1.8274 | 0.033823 | ZMYM2 |
| ENSG00000165996 | 10 | 17631958 | 17659376 | 103 | 18 | 5925 | 1.8266 | 0.033879 | PTPLA |
| ENSG00000164535 | 7 | 6448757 | 6523821 | 315 | 35 | 5925 | 1.8265 | 0.03389 | DAGLB |
| ENSG00000166349 | 11 | 36532259 | 36614706 | 158 | 21 | 5925 | 1.8249 | 0.034009 | RAG1 |
| ENSG00000174255 | 3 | 113953483 | 113956425 | 13 | 5 | 5925 | 1.8244 | 0.034045 | ZNF80 |
| ENSG00000166959 | 11 | 60467047 | 60483284 | 14 | 5 | 5925 | 1.8241 | 0.034067 | MS4A8 |
| ENSG00000009830 | 14 | 77741299 | 77787227 | 156 | 29 | 5925 | 1.8237 | 0.034097 | POMT2 |
| ENSG00000155816 | 1 | 240177648 | 240638489 | 1670 | 100 | 5925 | 1.8234 | 0.03412 | FMN2 |
| ENSG00000168899 | 2 | 85811531 | 85820535 | 24 | 7 | 5925 | 1.8226 | 0.034179 | VAMP5 |
| ENSG00000162814 | 1 | 217804666 | 218045038 | 747 | 42 | 5925 | 1.8196 | 0.03441 | SPATA17 |
| ENSG00000166352 | 11 | 36616051 | 36694823 | 274 | 12 | 5925 | 1.8187 | 0.034476 | C11orf74 |
| ENSG00000105127 | 19 | 15464196 | 15490609 | 46 | 11 | 5925 | 1.8182 | 0.034513 | AKAP8 |
| ENSG00000204634 | 2 | 101624079 | 101869328 | 596 | 43 | 5925 | 1.8181 | 0.034521 | TBC1D8 |
| ENSG00000140044 | 14 | 75894419 | 75940814 | 144 | 26 | 5925 | 1.8176 | 0.03456 | JDP2 |
| ENSG00000169715 | 16 | 56659387 | 56661024 | 8 | 4 | 5925 | 1.8175 | 0.03457 | MT1E |
| ENSG00000107263 | 9 | 134452157 | 134615461 | 426 | 30 | 5925 | 1.816 | 0.034682 | RAPGEF1 |
| ENSG00000105479 | 19 | 48799714 | 48825151 | 86 | 11 | 5925 | 1.8156 | 0.034719 | CCDC114 |
| ENSG00000146282 | 6 | 88224096 | 88299721 | 246 | 18 | 5925 | 1.8147 | 0.034784 | RARS2 |
| ENSG00000066583 | 5 | 128430444 | 128449721 | 70 | 14 | 5925 | 1.8137 | 0.034865 | ISOC1 |
| ENSG00000233927 | 19 | 8386042 | 8388224 | 12 | 5 | 5925 | 1.8131 | 0.034911 | RPS28 |
| ENSG00000144736 | 3 | 72798428 | 72911065 | 167 | 12 | 5925 | 1.8129 | 0.034926 | SHQ1 |
| ENSG00000137098 | 9 | 35808042 | 35812269 | 5 | 2 | 5925 | 1.8123 | 0.034967 | SPAG8 |
| ENSG00000165171 | 7 | 73248920 | 73256865 | 16 | 1 | 5925 | 1.8112 | 0.035055 | WBSCR27 |
| ENSG00000085741 | 11 | 75897369 | 75921780 | 74 | 15 | 5925 | 1.8094 | 0.035197 | WNT11 |
| ENSG00000146476 | 6 | 151773422 | 151791236 | 52 | 10 | 5925 | 1.8093 | 0.035205 | C6orf211 |
| ENSG00000176231 | 19 | 16059818 | 16060768 | 6 | 2 | 5925 | 1.8087 | 0.03525 | OR10H4 |
| ENSG00000010244 | 17 | 30677136 | 30708905 | 53 | 11 | 5925 | 1.8082 | 0.035289 | ZNF207 |
| ENSG00000110203 | 11 | 71825915 | 71850936 | 96 | 12 | 5925 | 1.8076 | 0.035332 | FOLR3 |
| ENSG00000169738 | 17 | 79993012 | 79995608 | 13 | 1 | 5925 | 1.8065 | 0.03542 | DCXR |
| ENSG00000126218 | 13 | 113777128 | 113803843 | 66 | 23 | 5925 | 1.8063 | 0.035437 | F10 |
| ENSG00000165078 | 8 | 68334360 | 68658620 | 892 | 68 | 5925 | 1.8044 | 0.035583 | CPA6 |
| ENSG00000126088 | 1 | 45477819 | 45481247 | 8 | 3 | 5925 | 1.8008 | 0.035865 | UROD |
| ENSG00000136146 | 13 | 48627459 | 48669267 | 142 | 22 | 5925 | 1.7995 | 0.035967 | MED4 |
| ENSG00000162390 | 1 | 55007930 | 55104865 | 399 | 38 | 5925 | 1.7993 | 0.035986 | ACOT11 |
| ENSG00000214919 | 3 | 155459933 | 155461515 | 4 | 2 | 5925 | 1.7947 | 0.036348 | AC104472.1 |
| ENSG00000114248 | 3 | 169556967 | 169587718 | 79 | 12 | 5925 | 1.7943 | 0.036386 | LRRC31 |
| ENSG00000006611 | 11 | 17515442 | 17565963 | 159 | 28 | 5925 | 1.7935 | 0.036444 | USH1C |
| ENSG00000164330 | 5 | 158122928 | 158526769 | 805 | 62 | 5925 | 1.7933 | 0.03646 | EBF1 |
| ENSG00000175866 | 17 | 79008948 | 79091232 | 390 | 54 | 5925 | 1.7933 | 0.036461 | BAIAP2 |
| ENSG00000258053 | 12 | 71441182 | 71512027 | 173 | 15 | 5925 | 1.7931 | 0.036478 | CTD-2021H9.3 |
| ENSG00000080511 | 19 | 10123925 | 10132955 | 47 | 8 | 5925 | 1.792 | 0.036564 | RDH8 |
| ENSG00000168079 | 8 | 27727399 | 27850244 | 545 | 54 | 5925 | 1.7918 | 0.036586 | SCARA5 |
| ENSG00000221963 | 22 | 36044442 | 36064456 | 64 | 9 | 5925 | 1.7894 | 0.036777 | APOL6 |
| ENSG00000104332 | 8 | 41119481 | 41167016 | 123 | 17 | 5925 | 1.7882 | 0.036871 | SFRP1 |
| ENSG00000143819 | 1 | 225997794 | 226033260 | 123 | 36 | 5925 | 1.7881 | 0.036883 | EPHX1 |
| ENSG00000138821 | 4 | 103172198 | 103352415 | 514 | 37 | 5925 | 1.7864 | 0.037019 | SLC39A8 |
| ENSG00000172728 | 8 | 33228342 | 33330940 | 328 | 23 | 5925 | 1.7859 | 0.037057 | FUT10 |
| ENSG00000196950 | 2 | 196440701 | 196602426 | 330 | 32 | 5925 | 1.7856 | 0.037079 | SLC39A10 |
| ENSG00000168003 | 11 | 62623518 | 62656352 | 28 | 7 | 5925 | 1.7846 | 0.037159 | SLC3A2 |
| ENSG00000153936 | 1 | 87380331 | 87602334 | 476 | 20 | 5925 | 1.7845 | 0.037169 | HS2ST1 |
| ENSG00000151632 | 10 | 5029967 | 5060223 | 135 | 22 | 5925 | 1.7843 | 0.037189 | AKR1C2 |
| ENSG00000183091 | 2 | 152341850 | 152591001 | 438 | 45 | 5925 | 1.7827 | 0.037318 | NEB |
| ENSG00000182575 | 17 | 47653220 | 47661189 | 10 | 3 | 5925 | 1.7825 | 0.03733 | NXPH3 |
| ENSG00000186306 | 1 | 158368312 | 158369256 | 8 | 3 | 5925 | 1.7816 | 0.037409 | OR10T2 |
| ENSG00000103018 | 16 | 69458428 | 69500169 | 136 | 18 | 5925 | 1.7795 | 0.037577 | CYB5B |
| ENSG00000268628 | 20 | 19738296 | 19738679 | 2 | 1 | 5925 | 1.7781 | 0.03769 | AL121761.2 |
| ENSG00000185946 | 1 | 104068313 | 104097861 | 45 | 11 | 5925 | 1.7759 | 0.037878 | RNPC3 |
| ENSG00000131914 | 1 | 26737269 | 26756213 | 41 | 13 | 5925 | 1.775 | 0.037949 | LIN28A |
| ENSG00000105991 | 7 | 27132612 | 27135615 | 5 | 3 | 5925 | 1.7747 | 0.037973 | HOXA1 |
| ENSG00000240871 | 17 | 39240459 | 39241396 | 5 | 1 | 5925 | 1.7747 | 0.037977 | KRTAP4-7 |
| ENSG00000117625 | 1 | 211431719 | 211489727 | 183 | 26 | 5925 | 1.7745 | 0.03799 | RCOR3 |
| ENSG00000140575 | 15 | 90931450 | 91045475 | 306 | 17 | 5925 | 1.7725 | 0.038156 | IQGAP1 |
| ENSG00000111674 | 12 | 7022909 | 7032861 | 13 | 4 | 5925 | 1.7723 | 0.038172 | ENO2 |
| ENSG00000100804 | 14 | 23485752 | 23504439 | 28 | 8 | 5925 | 1.7722 | 0.038184 | PSMB5 |
| ENSG00000143033 | 1 | 93544792 | 93604638 | 95 | 12 | 5925 | 1.7719 | 0.038203 | MTF2 |
| ENSG00000111012 | 12 | 58156117 | 58162769 | 7 | 2 | 5925 | 1.7719 | 0.038207 | CYP27B1 |
| ENSG00000166592 | 16 | 66955582 | 66959547 | 5 | 2 | 5925 | 1.7718 | 0.038217 | RRAD |
| ENSG00000130701 | 20 | 60985293 | 61002589 | 56 | 16 | 5925 | 1.7704 | 0.038334 | RBBP8NL |
| ENSG00000106178 | 7 | 75440983 | 75452674 | 61 | 14 | 5925 | 1.7692 | 0.038434 | CCL24 |
| ENSG00000117335 | 1 | 207925402 | 207968858 | 98 | 13 | 5925 | 1.7691 | 0.038439 | CD46 |
| ENSG00000157870 | 1 | 2517930 | 2522908 | 10 | 4 | 5925 | 1.7686 | 0.038478 | FAM213B |
| ENSG00000111237 | 12 | 110928902 | 110939922 | 22 | 5 | 5925 | 1.7685 | 0.038492 | VPS29 |
| ENSG00000165637 | 10 | 76969912 | 76991206 | 48 | 13 | 5925 | 1.768 | 0.038531 | VDAC2 |
| ENSG00000133226 | 1 | 24958207 | 24999758 | 46 | 9 | 5925 | 1.7675 | 0.038571 | SRRM1 |
| ENSG00000162543 | 1 | 20512578 | 20522541 | 10 | 4 | 5925 | 1.7671 | 0.038605 | UBXN10 |
| ENSG00000165698 | 9 | 135753414 | 135765588 | 16 | 6 | 5925 | 1.7666 | 0.038648 | C9orf9 |
| ENSG00000164938 | 8 | 95938200 | 95961639 | 56 | 9 | 5925 | 1.766 | 0.038694 | TP53INP1 |
| ENSG00000187627 | 2 | 87135076 | 87241104 | 5 | 3 | 5925 | 1.7655 | 0.038743 | RGPD1 |
| ENSG00000216490 | 19 | 18283972 | 18288927 | 14 | 3 | 5925 | 1.7653 | 0.03876 | IFI30 |
| ENSG00000196772 | 1 | 247978102 | 247979031 | 4 | 2 | 5925 | 1.7651 | 0.038777 | OR14A16 |
| ENSG00000258989 | 14 | 61995846 | 62124682 | 344 | 55 | 5925 | 1.7648 | 0.038799 | RP11-47I22.4 |
| ENSG00000181143 | 19 | 8959520 | 9092018 | 495 | 51 | 5925 | 1.764 | 0.038863 | MUC16 |
| ENSG00000165312 | 10 | 23728198 | 23731308 | 5 | 3 | 5925 | 1.764 | 0.038865 | OTUD1 |
| ENSG00000133105 | 13 | 32313674 | 32377009 | 190 | 19 | 5925 | 1.764 | 0.038867 | RXFP2 |
| ENSG00000108294 | 17 | 36908989 | 36920484 | 29 | 11 | 5925 | 1.7635 | 0.038906 | PSMB3 |
| ENSG00000203733 | 6 | 142454227 | 142456288 | 5 | 3 | 5925 | 1.7631 | 0.038938 | GJE1 |
| ENSG00000149654 | 20 | 44802372 | 44937137 | 312 | 48 | 5925 | 1.7628 | 0.038964 | CDH22 |
| ENSG00000175097 | 11 | 36597124 | 36619829 | 60 | 7 | 5925 | 1.7628 | 0.038969 | RAG2 |
| ENSG00000096395 | 6 | 33762450 | 33771788 | 37 | 8 | 5925 | 1.7627 | 0.038971 | MLN |
| ENSG00000119242 | 12 | 124403207 | 124457378 | 115 | 10 | 5925 | 1.7618 | 0.039048 | CCDC92 |
| ENSG00000101544 | 18 | 77866915 | 77905406 | 136 | 14 | 5925 | 1.7614 | 0.039089 | ADNP2 |
| ENSG00000125788 | 20 | 123010 | 126392 | 7 | 3 | 5925 | 1.7607 | 0.039147 | DEFB126 |
| ENSG00000249852 | 7 | 227554 | 229557 | 17 | 5 | 5925 | 1.7607 | 0.039148 | AC145676.2 |
| ENSG00000132182 | 3 | 13357737 | 13461809 | 345 | 20 | 5925 | 1.7603 | 0.039179 | NUP210 |
| ENSG00000136167 | 13 | 46700055 | 46786006 | 268 | 39 | 5925 | 1.76 | 0.0392 | LCP1 |
| ENSG00000040608 | 22 | 20228938 | 20270769 | 89 | 21 | 5925 | 1.7597 | 0.03923 | RTN4R |
| ENSG00000221995 | 17 | 27400537 | 27418537 | 48 | 8 | 5925 | 1.7583 | 0.039348 | TIAF1 |
| ENSG00000146005 | 5 | 139175406 | 139224051 | 90 | 12 | 5925 | 1.7573 | 0.039437 | PSD2 |
| ENSG00000104833 | 19 | 6494330 | 6502859 | 22 | 10 | 5925 | 1.7571 | 0.039451 | TUBB4A |
| ENSG00000162695 | 1 | 101361632 | 101447309 | 133 | 16 | 5925 | 1.7566 | 0.039493 | SLC30A7 |
| ENSG00000196844 | 11 | 125646008 | 125648714 | 11 | 3 | 5925 | 1.7558 | 0.039557 | PATE2 |
| ENSG00000186094 | 1 | 48998527 | 50489585 | 2255 | 65 | 5925 | 1.7557 | 0.039566 | AGBL4 |
| ENSG00000073146 | 22 | 50528308 | 50600119 | 377 | 16 | 5925 | 1.7557 | 0.039568 | MOV10L1 |
| ENSG00000140859 | 16 | 57792129 | 57896957 | 388 | 48 | 5925 | 1.7557 | 0.039572 | KIFC3 |
| ENSG00000168995 | 19 | 51645556 | 51656783 | 17 | 6 | 5925 | 1.7554 | 0.039592 | SIGLEC7 |
| ENSG00000071894 | 8 | 145618444 | 145634753 | 22 | 4 | 5925 | 1.7554 | 0.039599 | CPSF1 |
| ENSG00000170852 | 7 | 32907784 | 32933743 | 54 | 9 | 5925 | 1.7546 | 0.039662 | KBTBD2 |
| ENSG00000182853 | 17 | 4688580 | 4689728 | 3 | 1 | 5925 | 1.7541 | 0.039709 | VMO1 |
| ENSG00000174453 | 2 | 215275789 | 215443683 | 461 | 35 | 5925 | 1.7526 | 0.039835 | VWC2L |
| ENSG00000105679 | 19 | 36024314 | 36036218 | 34 | 11 | 5925 | 1.7525 | 0.039841 | GAPDHS |
| ENSG00000006210 | 16 | 57406370 | 57418960 | 41 | 11 | 5925 | 1.7524 | 0.039855 | CX3CL1 |
| ENSG00000166211 | 12 | 101869199 | 101880775 | 35 | 10 | 5925 | 1.7517 | 0.039913 | SPIC |
| ENSG00000088448 | 13 | 111530887 | 111567416 | 100 | 10 | 5925 | 1.7516 | 0.039924 | ANKRD10 |
| ENSG00000156671 | 10 | 76859344 | 76941881 | 169 | 20 | 5925 | 1.7513 | 0.039944 | SAMD8 |
| ENSG00000267467 | 19 | 45445495 | 45452820 | 24 | 5 | 5925 | 1.7504 | 0.040029 | APOC4 |
| ENSG00000224916 | 19 | 45445495 | 45452822 | 24 | 5 | 5925 | 1.7504 | 0.040029 | APOC4-APOC2 |
| ENSG00000119705 | 14 | 78174414 | 78227447 | 199 | 21 | 5925 | 1.7492 | 0.04013 | SLIRP |
| ENSG00000158769 | 1 | 160965001 | 160991138 | 71 | 7 | 5925 | 1.7491 | 0.04014 | F11R |
| ENSG00000198090 | 17 | 39295685 | 39296739 | 5 | 3 | 5925 | 1.7484 | 0.040194 | KRTAP4-6 |
| ENSG00000270149 | 1 | 160967747 | 161008752 | 102 | 14 | 5925 | 1.7484 | 0.040199 | RP11-544M22.13 |
| ENSG00000125457 | 17 | 73262309 | 73267308 | 3 | 1 | 5925 | 1.7481 | 0.040221 | MIF4GD |
| ENSG00000197302 | 16 | 31724550 | 31806190 | 157 | 13 | 5925 | 1.748 | 0.040234 | ZNF720 |
| ENSG00000143924 | 2 | 42396490 | 42559688 | 485 | 25 | 5925 | 1.7478 | 0.040247 | EML4 |
| ENSG00000113262 | 5 | 178405328 | 178423207 | 94 | 12 | 5925 | 1.7466 | 0.040357 | GRM6 |
| ENSG00000227450 | 17 | 36905613 | 36906969 | 3 | 2 | 5925 | 1.7456 | 0.040438 | CTB-58E17.5 |
| ENSG00000164176 | 5 | 83236373 | 83680611 | 945 | 95 | 5925 | 1.7448 | 0.04051 | EDIL3 |
| ENSG00000125944 | 1 | 23630264 | 23670829 | 36 | 7 | 5925 | 1.7447 | 0.040514 | HNRNPR |
| ENSG00000100629 | 14 | 80943330 | 81425861 | 1538 | 57 | 5925 | 1.7437 | 0.04061 | CEP128 |
| ENSG00000099904 | 22 | 20116979 | 20135530 | 62 | 9 | 5925 | 1.7436 | 0.04061 | ZDHHC8 |
| ENSG00000163815 | 3 | 45043040 | 45077563 | 100 | 11 | 5925 | 1.7432 | 0.040652 | CLEC3B |
| ENSG00000149575 | 11 | 118032666 | 118047388 | 45 | 12 | 5925 | 1.743 | 0.040669 | SCN2B |
| ENSG00000085644 | 16 | 3179778 | 3192806 | 18 | 4 | 5925 | 1.7423 | 0.040731 | ZNF213 |
| ENSG00000176749 | 17 | 30813637 | 30818274 | 3 | 1 | 5925 | 1.7412 | 0.040826 | CDK5R1 |
| ENSG00000136487 | 17 | 61957578 | 61959295 | 2 | 1 | 5925 | 1.7409 | 0.040849 | GH2 |
| ENSG00000167769 | 19 | 6306153 | 6333640 | 115 | 18 | 5925 | 1.7399 | 0.040937 | ACER1 |
| ENSG00000129696 | 8 | 33330904 | 33371119 | 127 | 11 | 5925 | 1.7398 | 0.040945 | TTI2 |
| ENSG00000140829 | 16 | 72127461 | 72146811 | 57 | 9 | 5925 | 1.7393 | 0.040988 | DHX38 |
| ENSG00000125447 | 17 | 73232694 | 73258444 | 44 | 7 | 5925 | 1.7389 | 0.041027 | GGA3 |
| ENSG00000105865 | 7 | 107203929 | 107218906 | 21 | 7 | 5925 | 1.7385 | 0.041065 | DUS4L |
| ENSG00000048707 | 1 | 12290124 | 12572099 | 534 | 23 | 5925 | 1.7377 | 0.041134 | VPS13D |
| ENSG00000196578 | 3 | 97806017 | 97806946 | 5 | 3 | 5925 | 1.7367 | 0.041219 | OR5AC2 |
| ENSG00000186073 | 15 | 36871812 | 37102449 | 619 | 50 | 5925 | 1.7365 | 0.041239 | C15orf41 |
| ENSG00000037897 | 12 | 58162254 | 58166576 | 5 | 2 | 5925 | 1.7361 | 0.041274 | METTL1 |
| ENSG00000204880 | 17 | 39253233 | 39254393 | 10 | 2 | 5925 | 1.7359 | 0.041291 | KRTAP4-8 |
| ENSG00000078246 | 12 | 2986389 | 3050306 | 203 | 7 | 5925 | 1.7337 | 0.041486 | TULP3 |
| ENSG00000198785 | 9 | 104331635 | 104500862 | 660 | 30 | 5925 | 1.7334 | 0.041508 | GRIN3A |
| ENSG00000225781 | 7 | 142749417 | 142750444 | 5 | 2 | 5925 | 1.7306 | 0.041765 | OR6V1 |
| ENSG00000197980 | 3 | 156543270 | 156763918 | 632 | 27 | 5925 | 1.73 | 0.041819 | LEKR1 |
| ENSG00000134627 | 11 | 94277006 | 94354587 | 195 | 26 | 5925 | 1.7297 | 0.041846 | PIWIL4 |
| ENSG00000162946 | 1 | 231762561 | 232177018 | 1168 | 124 | 5925 | 1.7292 | 0.041889 | DISC1 |
| ENSG00000134183 | 1 | 110145889 | 110155679 | 21 | 8 | 5925 | 1.7291 | 0.041896 | GNAT2 |
| ENSG00000238244 | 15 | 90890819 | 90892669 | 12 | 2 | 5925 | 1.7283 | 0.041969 | GABARAPL3 |
| ENSG00000140332 | 15 | 70340129 | 70390515 | 157 | 26 | 5925 | 1.7281 | 0.041989 | TLE3 |
| ENSG00000167315 | 18 | 47309869 | 47340330 | 65 | 12 | 5925 | 1.7279 | 0.042001 | ACAA2 |
| ENSG00000250722 | 5 | 42799982 | 42887494 | 259 | 26 | 5925 | 1.7276 | 0.042027 | SEPP1 |
| ENSG00000128000 | 19 | 40534167 | 40562116 | 58 | 5 | 5925 | 1.7274 | 0.042047 | ZNF780B |
| ENSG00000129116 | 4 | 169418217 | 169849608 | 1375 | 115 | 5925 | 1.7272 | 0.04207 | PALLD |
| ENSG00000139549 | 12 | 49483204 | 49488602 | 6 | 3 | 5925 | 1.7269 | 0.042088 | DHH |
| ENSG00000100347 | 22 | 44351301 | 44406411 | 205 | 28 | 5925 | 1.7263 | 0.042146 | SAMM50 |
| ENSG00000141968 | 19 | 6772725 | 6857377 | 317 | 52 | 5925 | 1.7262 | 0.042153 | VAV1 |
| ENSG00000178999 | 17 | 8108056 | 8113918 | 14 | 6 | 5925 | 1.7254 | 0.042226 | AURKB |
| ENSG00000171481 | 9 | 125437315 | 125438432 | 1 | 1 | 5925 | 1.7254 | 0.04223 | OR1L3 |
| ENSG00000066322 | 1 | 43829068 | 43833696 | 8 | 5 | 5925 | 1.7248 | 0.042281 | ELOVL1 |
| ENSG00000164924 | 8 | 101928753 | 101965616 | 97 | 13 | 5925 | 1.7242 | 0.042331 | YWHAZ |
| ENSG00000186090 | 3 | 183749332 | 183757157 | 38 | 9 | 5925 | 1.7234 | 0.042405 | HTR3D |
| ENSG00000171103 | 2 | 29072687 | 29093167 | 33 | 6 | 5925 | 1.7233 | 0.042415 | TRMT61B |
| ENSG00000169764 | 2 | 64068074 | 64118696 | 123 | 24 | 5925 | 1.7233 | 0.042421 | UGP2 |
| ENSG00000104331 | 8 | 57870492 | 57906403 | 79 | 10 | 5925 | 1.7228 | 0.042458 | IMPAD1 |
| ENSG00000188175 | 7 | 92817899 | 92855837 | 68 | 19 | 5925 | 1.7227 | 0.042467 | HEPACAM2 |
| ENSG00000205302 | 5 | 122110691 | 122165803 | 212 | 23 | 5925 | 1.7222 | 0.042518 | SNX2 |
| ENSG00000004660 | 17 | 3763609 | 3798185 | 128 | 35 | 5925 | 1.7222 | 0.04252 | CAMKK1 |
| ENSG00000153113 | 5 | 95860971 | 96115299 | 829 | 71 | 5925 | 1.7218 | 0.042552 | CAST |
| ENSG00000136997 | 8 | 128747680 | 128753674 | 19 | 9 | 5925 | 1.7211 | 0.042612 | MYC |
| ENSG00000188807 | 1 | 9648932 | 9674935 | 42 | 9 | 5925 | 1.7211 | 0.042616 | TMEM201 |
| ENSG00000140948 | 16 | 87439852 | 87525651 | 337 | 17 | 5925 | 1.7206 | 0.04266 | ZCCHC14 |
| ENSG00000055813 | 2 | 56411258 | 56613308 | 639 | 57 | 5925 | 1.7191 | 0.042802 | CCDC85A |
| ENSG00000101752 | 18 | 19284918 | 19450918 | 245 | 24 | 5925 | 1.7185 | 0.042854 | MIB1 |
| ENSG00000122367 | 10 | 88428206 | 88495825 | 264 | 34 | 5925 | 1.7173 | 0.04296 | LDB3 |
| ENSG00000103274 | 16 | 10837643 | 10863208 | 75 | 13 | 5925 | 1.7165 | 0.043033 | NUBP1 |
| ENSG00000171345 | 17 | 39679869 | 39684560 | 7 | 1 | 5925 | 1.7156 | 0.043122 | KRT19 |
| ENSG00000177025 | 19 | 58469805 | 58485902 | 49 | 8 | 5925 | 1.7138 | 0.043281 | C19orf18 |
| ENSG00000205129 | 4 | 186347394 | 186370980 | 69 | 10 | 5925 | 1.7132 | 0.04334 | C4orf47 |
| ENSG00000182912 | 21 | 45937099 | 45945835 | 18 | 6 | 5925 | 1.7118 | 0.04347 | C21orf90 |
| ENSG00000198373 | 16 | 69796209 | 69975644 | 470 | 30 | 5925 | 1.7113 | 0.043516 | WWP2 |
| ENSG00000068001 | 3 | 50355221 | 50360337 | 2 | 1 | 5925 | 1.7098 | 0.04365 | HYAL2 |
| ENSG00000197653 | 12 | 124247042 | 124420753 | 483 | 48 | 5925 | 1.7079 | 0.043831 | DNAH10 |
| ENSG00000132122 | 1 | 48761044 | 48937845 | 307 | 30 | 5925 | 1.7078 | 0.04384 | SPATA6 |
| ENSG00000007541 | 16 | 616995 | 634136 | 58 | 5 | 5925 | 1.7076 | 0.043855 | PIGQ |
| ENSG00000074416 | 3 | 127407909 | 127542051 | 412 | 55 | 5925 | 1.7055 | 0.044047 | MGLL |
| ENSG00000171505 | 9 | 125288637 | 125289572 | 5 | 3 | 5925 | 1.704 | 0.044189 | OR1N1 |
| ENSG00000125834 | 20 | 2082257 | 2157684 | 234 | 27 | 5925 | 1.7036 | 0.044226 | STK35 |
| ENSG00000185681 | 9 | 124922190 | 124962367 | 126 | 23 | 5925 | 1.7034 | 0.044247 | MORN5 |
| ENSG00000186834 | 17 | 43224684 | 43229468 | 7 | 3 | 5925 | 1.7028 | 0.0443 | HEXIM1 |
| ENSG00000196914 | 11 | 120207787 | 120360645 | 276 | 17 | 5925 | 1.701 | 0.044471 | ARHGEF12 |
| ENSG00000160305 | 21 | 47878812 | 47989926 | 412 | 30 | 5925 | 1.7006 | 0.04451 | DIP2A |
| ENSG00000173846 | 1 | 45265897 | 45271662 | 10 | 3 | 5925 | 1.6995 | 0.044617 | PLK3 |
| ENSG00000197705 | 18 | 30252634 | 30353025 | 262 | 11 | 5925 | 1.6986 | 0.0447 | KLHL14 |
| ENSG00000253976 | 8 | 56047763 | 56054918 | 22 | 6 | 5925 | 1.6984 | 0.04472 | RP11-386G21.1 |
| ENSG00000212657 | 17 | 39463952 | 39465505 | 8 | 2 | 5925 | 1.698 | 0.044757 | KRTAP16-1 |
| ENSG00000079387 | 12 | 48436681 | 48500091 | 164 | 17 | 5925 | 1.6965 | 0.044892 | SENP1 |
| ENSG00000100092 | 22 | 38030661 | 38062939 | 121 | 16 | 5925 | 1.6965 | 0.0449 | SH3BP1 |
| ENSG00000168509 | 1 | 145413095 | 145417545 | 3 | 2 | 5925 | 1.6964 | 0.044908 | HFE2 |
| ENSG00000089356 | 19 | 35606732 | 35615228 | 33 | 9 | 5925 | 1.6958 | 0.04496 | FXYD3 |
| ENSG00000160161 | 19 | 19649057 | 19657468 | 19 | 3 | 5925 | 1.6957 | 0.044972 | CILP2 |
| ENSG00000212658 | 17 | 39458078 | 39459103 | 4 | 1 | 5925 | 1.6954 | 0.045001 | KRTAP29-1 |
| ENSG00000145808 | 5 | 128795958 | 129074376 | 726 | 38 | 5925 | 1.6947 | 0.04507 | ADAMTS19 |
| ENSG00000099901 | 22 | 20103461 | 20114878 | 26 | 7 | 5925 | 1.6945 | 0.045086 | RANBP1 |
| ENSG00000154556 | 4 | 186506598 | 186877806 | 1611 | 149 | 5925 | 1.6942 | 0.045114 | SORBS2 |
| ENSG00000132677 | 1 | 156339003 | 156355011 | 52 | 11 | 5925 | 1.6927 | 0.045257 | RHBG |
| ENSG00000175344 | 15 | 32322691 | 32464722 | 277 | 43 | 5925 | 1.6927 | 0.045257 | CHRNA7 |
| ENSG00000121691 | 11 | 34460472 | 34493609 | 80 | 11 | 5925 | 1.6921 | 0.045315 | CAT |
| ENSG00000103540 | 16 | 19535133 | 19564730 | 92 | 12 | 5925 | 1.6917 | 0.045356 | CCP110 |
| ENSG00000198331 | 11 | 125753509 | 125770543 | 43 | 7 | 5925 | 1.691 | 0.045419 | HYLS1 |
| ENSG00000196187 | 1 | 226033237 | 226070069 | 109 | 15 | 5925 | 1.6903 | 0.04549 | TMEM63A |
| ENSG00000008516 | 16 | 3096682 | 3110727 | 49 | 10 | 5925 | 1.6898 | 0.04553 | MMP25 |
| ENSG00000198812 | 12 | 70002351 | 70004942 | 5 | 3 | 5925 | 1.6895 | 0.045565 | LRRC10 |
| ENSG00000124587 | 6 | 42931608 | 42946958 | 54 | 9 | 5925 | 1.689 | 0.045607 | PEX6 |
| ENSG00000129221 | 17 | 6297013 | 6338519 | 231 | 45 | 5925 | 1.6889 | 0.04562 | AIPL1 |
| ENSG00000100603 | 14 | 78183942 | 78227550 | 156 | 18 | 5925 | 1.6886 | 0.045651 | SNW1 |
| ENSG00000180626 | 17 | 5082831 | 5095178 | 28 | 10 | 5925 | 1.6878 | 0.045722 | ZNF594 |
| ENSG00000121270 | 16 | 48200821 | 48281479 | 234 | 12 | 5925 | 1.6877 | 0.045731 | ABCC11 |
| ENSG00000160882 | 8 | 143953772 | 143961262 | 42 | 6 | 5925 | 1.6865 | 0.045853 | CYP11B1 |
| ENSG00000180855 | 19 | 12540521 | 12551926 | 73 | 5 | 5925 | 1.6862 | 0.045876 | ZNF443 |
| ENSG00000198271 | 17 | 39305178 | 39306054 | 4 | 2 | 5925 | 1.686 | 0.045896 | KRTAP4-5 |
| ENSG00000172461 | 6 | 96463860 | 96663488 | 719 | 43 | 5925 | 1.6856 | 0.045935 | FUT9 |
| ENSG00000180389 | 13 | 28519343 | 28519727 | 3 | 2 | 5925 | 1.6841 | 0.046085 | ATP5EP2 |
| ENSG00000253831 | 1 | 157061836 | 157069600 | 41 | 12 | 5925 | 1.6822 | 0.046264 | ETV3L |
| ENSG00000255835 | 1 | 226074396 | 226111978 | 109 | 20 | 5925 | 1.682 | 0.04628 | RP4-559A3.7 |
| ENSG00000125841 | 20 | 327426 | 340304 | 25 | 8 | 5925 | 1.6817 | 0.046309 | NRSN2 |
| ENSG00000235931 | 10 | 61715188 | 61720671 | 18 | 4 | 5925 | 1.6809 | 0.046394 | C10orf40 |
| ENSG00000125247 | 13 | 101256181 | 101327347 | 216 | 18 | 5925 | 1.68 | 0.046477 | TMTC4 |
| ENSG00000154451 | 1 | 89724633 | 89738544 | 21 | 6 | 5925 | 1.6798 | 0.046501 | GBP5 |
| ENSG00000046604 | 18 | 29078006 | 29128971 | 139 | 18 | 5925 | 1.6797 | 0.04651 | DSG2 |
| ENSG00000123144 | 19 | 12841454 | 12845589 | 1 | 1 | 5925 | 1.6793 | 0.04655 | C19orf43 |
| ENSG00000133835 | 5 | 118788138 | 118972894 | 611 | 37 | 5925 | 1.678 | 0.04667 | HSD17B4 |
| ENSG00000137496 | 11 | 71709587 | 71716761 | 12 | 3 | 5925 | 1.6779 | 0.046687 | IL18BP |
| ENSG00000124942 | 11 | 62201016 | 62323707 | 269 | 45 | 5925 | 1.6778 | 0.046695 | AHNAK |
| ENSG00000123213 | 5 | 65018023 | 65167553 | 468 | 45 | 5925 | 1.6769 | 0.046784 | NLN |
| ENSG00000104643 | 8 | 11141925 | 11185646 | 130 | 14 | 5925 | 1.6762 | 0.046852 | MTMR9 |
| ENSG00000116604 | 1 | 156433519 | 156470620 | 73 | 12 | 5925 | 1.6761 | 0.046856 | MEF2D |
| ENSG00000258429 | 16 | 69362524 | 69364498 | 8 | 4 | 5925 | 1.6759 | 0.046874 | PDF |
| ENSG00000196670 | 5 | 180274611 | 180288286 | 49 | 8 | 5925 | 1.6756 | 0.04691 | ZFP62 |
| ENSG00000160654 | 11 | 118215059 | 118225876 | 21 | 6 | 5925 | 1.6753 | 0.046936 | CD3G |
| ENSG00000176563 | 17 | 40950810 | 40963605 | 8 | 4 | 5925 | 1.6743 | 0.047041 | CNTD1 |
| ENSG00000235608 | 4 | 1396720 | 1400119 | 4 | 2 | 5925 | 1.6741 | 0.047058 | NKX1-1 |
| ENSG00000198042 | 8 | 33342268 | 33358778 | 49 | 8 | 5925 | 1.6733 | 0.047132 | MAK16 |
| ENSG00000091039 | 12 | 76745577 | 76953589 | 441 | 23 | 5925 | 1.6732 | 0.047145 | OSBPL8 |
| ENSG00000112309 | 6 | 71566382 | 71666741 | 267 | 44 | 5925 | 1.6727 | 0.047188 | B3GAT2 |
| ENSG00000162391 | 1 | 55074855 | 55089229 | 50 | 15 | 5925 | 1.6727 | 0.047194 | FAM151A |
| ENSG00000255713 | 17 | 56247017 | 56247940 | 4 | 1 | 5925 | 1.6711 | 0.047356 | OR4D2 |
| ENSG00000183067 | 21 | 41117334 | 41174023 | 342 | 19 | 5925 | 1.6706 | 0.047404 | IGSF5 |
| ENSG00000140830 | 16 | 72078188 | 72128330 | 141 | 18 | 5925 | 1.6705 | 0.047415 | TXNL4B |
| ENSG00000115486 | 2 | 85771846 | 85788670 | 46 | 11 | 5925 | 1.6701 | 0.047455 | GGCX |
| ENSG00000137055 | 9 | 26904081 | 26947461 | 145 | 17 | 5925 | 1.6685 | 0.047605 | PLAA |
| ENSG00000119715 | 14 | 76776957 | 76968178 | 791 | 75 | 5925 | 1.6684 | 0.047614 | ESRRB |
| ENSG00000174780 | 4 | 57333081 | 57369839 | 109 | 12 | 5925 | 1.668 | 0.047661 | SRP72 |
| ENSG00000227839 | 1 | 50459990 | 50461874 | 3 | 2 | 5925 | 1.6674 | 0.047721 | AL645730.2 |
| ENSG00000152689 | 2 | 33661391 | 33789817 | 468 | 74 | 5925 | 1.667 | 0.047755 | RASGRP3 |
| ENSG00000268805 | 21 | 46511596 | 46515493 | 20 | 7 | 5925 | 1.6658 | 0.047877 | PRED57 |
| ENSG00000156959 | 3 | 9543481 | 9595486 | 195 | 20 | 5925 | 1.6656 | 0.047894 | LHFPL4 |
| ENSG00000033627 | 17 | 40610862 | 40674629 | 81 | 21 | 5925 | 1.6653 | 0.047922 | ATP6V0A1 |
| ENSG00000253857 | 8 | 56074043 | 56077287 | 8 | 3 | 5925 | 1.6651 | 0.047944 | RP11-386G21.2 |
| ENSG00000184445 | 12 | 123011793 | 123110943 | 176 | 20 | 5925 | 1.6636 | 0.048093 | KNTC1 |
| ENSG00000269121 | 16 | 12062000 | 12062333 | 1 | 1 | 5925 | 1.6627 | 0.04819 | AC007216.2 |
| ENSG00000100711 | 14 | 104182067 | 104200005 | 45 | 11 | 5925 | 1.6614 | 0.048319 | ZFYVE21 |
| ENSG00000121931 | 1 | 111489807 | 111506701 | 21 | 7 | 5925 | 1.6613 | 0.048322 | LRIF1 |
| ENSG00000197586 | 20 | 25176329 | 25207365 | 104 | 15 | 5925 | 1.6606 | 0.048398 | ENTPD6 |
| ENSG00000165283 | 9 | 35099888 | 35103154 | 4 | 1 | 5925 | 1.6594 | 0.048516 | STOML2 |
| ENSG00000170929 | 19 | 9203855 | 9204889 | 2 | 1 | 5925 | 1.6592 | 0.048533 | OR1M1 |
| ENSG00000011454 | 9 | 125703112 | 125867145 | 264 | 22 | 5925 | 1.6583 | 0.048631 | RABGAP1 |
| ENSG00000082641 | 17 | 46125691 | 46138849 | 24 | 7 | 5925 | 1.6572 | 0.048739 | NFE2L1 |
| ENSG00000221843 | 2 | 27799389 | 27805588 | 8 | 3 | 5925 | 1.657 | 0.048757 | C2orf16 |
| ENSG00000163945 | 4 | 1341054 | 1381837 | 165 | 20 | 5925 | 1.6557 | 0.048888 | UVSSA |
| ENSG00000177225 | 11 | 767220 | 777488 | 30 | 6 | 5925 | 1.6556 | 0.048903 | PDDC1 |
| ENSG00000119541 | 18 | 61056423 | 61089716 | 81 | 9 | 5925 | 1.6553 | 0.048929 | VPS4B |
| ENSG00000128567 | 7 | 131185021 | 131242976 | 184 | 42 | 5925 | 1.6553 | 0.048935 | PODXL |
| ENSG00000156282 | 21 | 31538241 | 31538971 | 1 | 1 | 5925 | 1.6542 | 0.04904 | CLDN17 |
| ENSG00000112874 | 5 | 102884556 | 102898494 | 39 | 7 | 5925 | 1.6529 | 0.049175 | NUDT12 |
| ENSG00000153130 | 4 | 141178440 | 141306880 | 326 | 39 | 5925 | 1.6509 | 0.049379 | SCOC |
| ENSG00000178691 | 17 | 30264037 | 30328064 | 147 | 7 | 5925 | 1.6504 | 0.04943 | SUZ12 |
| ENSG00000116690 | 1 | 186265405 | 186283694 | 24 | 6 | 5925 | 1.6492 | 0.049551 | PRG4 |
| ENSG00000254349 | 8 | 75512010 | 75735548 | 579 | 42 | 5925 | 1.6492 | 0.049557 | RP11-758M4.1 |
| ENSG00000183474 | 5 | 68856035 | 68890550 | 1 | 1 | 5925 | 1.6488 | 0.04959 | GTF2H2C |
| ENSG00000129219 | 17 | 4710391 | 4726729 | 53 | 15 | 5925 | 1.6485 | 0.049622 | PLD2 |
| ENSG00000153147 | 4 | 144434616 | 144478639 | 83 | 9 | 5925 | 1.6483 | 0.049645 | SMARCA5 |
| ENSG00000120075 | 17 | 46668619 | 46671323 | 8 | 4 | 5925 | 1.6465 | 0.049833 | HOXB5 |
| ENSG00000177354 | 10 | 50507187 | 50535537 | 88 | 16 | 5925 | 1.6464 | 0.049842 | C10orf71 |
| ENSG00000136197 | 7 | 42948325 | 42951904 | 13 | 3 | 5925 | 1.6462 | 0.049857 | C7orf25 |
| ENSG00000163093 | 2 | 170335688 | 170382432 | 121 | 22 | 5925 | 1.646 | 0.049879 | BBS5 |
| ENSG00000251569 | 2 | 170335990 | 170382432 | 121 | 22 | 5925 | 1.646 | 0.049879 | RP11-724O16.1 |
| ENSG00000183049 | 10 | 12391481 | 12877545 | 1916 | 185 | 5925 | 1.6457 | 0.04991 | CAMK1D |
| ENSG00000106588 | 7 | 42956460 | 42971822 | 30 | 3 | 5925 | 1.6455 | 0.049933 | PSMA2 |
| ENSG00000173335 | 20 | 23583047 | 23586513 | 6 | 2 | 5925 | 1.645 | 0.04998 | CST9 |

**Supplementary Table 7. The results of gene set analysis generated by FUMA**

| **Number of genes** | **BETA** | **BETA STD** | **SE** | **P** |
| --- | --- | --- | --- | --- |
| 69 | 0.39743 | 0.024084 | 0.094586 | 1.33E-05 |
| 28 | 0.63129 | 0.024396 | 0.15495 | 2.32E-05 |
| 19 | 0.72195 | 0.022988 | 0.18029 | 3.12E-05 |
| 54 | 0.33492 | 0.017961 | 0.10253 | 0.00054546 |
| 227 | 0.16749 | 0.018331 | 0.051843 | 0.0006186 |
| 89 | 0.24545 | 0.016883 | 0.078281 | 0.00085935 |
| 66 | 0.30196 | 0.017897 | 0.096479 | 0.00087627 |
| 42 | 0.34238 | 0.016199 | 0.11304 | 0.0012294 |
| 41 | 0.3515 | 0.016431 | 0.1174 | 0.0013785 |
| 217 | 0.17206 | 0.018417 | 0.057472 | 0.0013794 |
| 92 | 0.24214 | 0.016933 | 0.081122 | 0.0014201 |
| 326 | 0.11793 | 0.015426 | 0.039631 | 0.0014642 |
| 66 | 0.27744 | 0.016444 | 0.093707 | 0.0015369 |
| 18 | 0.51974 | 0.016108 | 0.17575 | 0.0015543 |
| 85 | 0.23575 | 0.015849 | 0.079929 | 0.0015938 |
| 23 | 0.44785 | 0.015688 | 0.15193 | 0.0016021 |
| 72 | 0.24333 | 0.015061 | 0.082987 | 0.0016857 |
| 22 | 0.50344 | 0.017248 | 0.172 | 0.0017132 |
| 197 | 0.15751 | 0.016073 | 0.054311 | 0.0018668 |
| 204 | 0.16173 | 0.01679 | 0.056267 | 0.0020272 |
| 12 | 0.60585 | 0.015334 | 0.21132 | 0.002075 |
| 94 | 0.22492 | 0.015898 | 0.078672 | 0.0021275 |
| 41 | 0.37553 | 0.017555 | 0.13184 | 0.0022004 |
| 60 | 0.31544 | 0.017829 | 0.11124 | 0.0022895 |
| 31 | 0.37802 | 0.01537 | 0.13411 | 0.0024134 |
| 276 | 0.13473 | 0.016237 | 0.047952 | 0.0024833 |
| 6 | 0.77416 | 0.013857 | 0.27628 | 0.0025411 |
| 14 | 0.59825 | 0.016354 | 0.21513 | 0.0027137 |
| 8 | 0.77972 | 0.016115 | 0.28114 | 0.0027769 |
| 188 | 0.16105 | 0.016058 | 0.058158 | 0.0028128 |
| 55 | 0.26673 | 0.014436 | 0.096792 | 0.0029311 |
| 31 | 0.42092 | 0.017114 | 0.15294 | 0.0029622 |
| 6 | 0.90574 | 0.016212 | 0.33015 | 0.0030432 |
| 10 | 0.67785 | 0.015662 | 0.24722 | 0.0030577 |
| 154 | 0.1605 | 0.014497 | 0.059096 | 0.0033079 |
| 290 | 0.11832 | 0.014612 | 0.043663 | 0.0033689 |
| 15 | 0.47923 | 0.01356 | 0.17832 | 0.0036027 |
| 254 | 0.13259 | 0.015339 | 0.049379 | 0.003629 |
| 23 | 0.43372 | 0.015193 | 0.16164 | 0.00365 |
| 126 | 0.19094 | 0.015612 | 0.071307 | 0.0037102 |
| 99 | 0.20041 | 0.014535 | 0.074959 | 0.0037565 |
| 368 | 0.10234 | 0.014207 | 0.038543 | 0.0039666 |
| 98 | 0.18483 | 0.013338 | 0.070097 | 0.0041888 |
| 12 | 0.71432 | 0.018079 | 0.27152 | 0.0042625 |
| 15 | 0.51838 | 0.014668 | 0.19731 | 0.0043079 |
| 87 | 0.2148 | 0.014609 | 0.081802 | 0.0043247 |
| 11 | 0.75138 | 0.018208 | 0.28663 | 0.0043818 |
| 45 | 0.33269 | 0.016291 | 0.12691 | 0.004382 |
| 60 | 0.24093 | 0.013618 | 0.092008 | 0.004419 |
| 28 | 0.38372 | 0.014829 | 0.14766 | 0.0046831 |
| 40 | 0.29146 | 0.013458 | 0.11254 | 0.0048046 |
| 138 | 0.17079 | 0.014609 | 0.065987 | 0.0048285 |
| 16 | 0.50929 | 0.014882 | 0.19686 | 0.0048435 |
| 11 | 0.6143 | 0.014886 | 0.23774 | 0.0048893 |
| 47 | 0.25484 | 0.012753 | 0.098849 | 0.0049713 |
| 302 | 0.11593 | 0.014605 | 0.045107 | 0.0050864 |
| 216 | 0.1271 | 0.013573 | 0.049599 | 0.0052009 |
| 208 | 0.13931 | 0.014602 | 0.054414 | 0.0052363 |
| 10 | 0.61021 | 0.014099 | 0.23916 | 0.0053682 |
| 29 | 0.36076 | 0.014188 | 0.14199 | 0.0055359 |
| 31 | 0.32592 | 0.013252 | 0.12853 | 0.0056148 |
| 140 | 0.15433 | 0.013296 | 0.061079 | 0.0057619 |
| 42 | 0.28649 | 0.013555 | 0.11369 | 0.0058733 |
| 23 | 0.39715 | 0.013912 | 0.15877 | 0.0061888 |
| 11 | 0.51708 | 0.01253 | 0.20831 | 0.0065316 |
| 8 | 0.71145 | 0.014704 | 0.28682 | 0.0065645 |
| 20 | 0.37605 | 0.012285 | 0.15205 | 0.0066992 |
| 199 | 0.12514 | 0.012834 | 0.050915 | 0.0069922 |
| 15 | 0.48809 | 0.01381 | 0.19863 | 0.0070053 |
| 135 | 0.1642 | 0.013893 | 0.066847 | 0.0070223 |
| 40 | 0.28792 | 0.013295 | 0.11755 | 0.0071599 |
| 12 | 0.52806 | 0.013365 | 0.21616 | 0.0072898 |
| 15 | 0.56825 | 0.016079 | 0.2337 | 0.0075221 |
| 21 | 0.45059 | 0.015083 | 0.18607 | 0.0077326 |
| 148 | 0.14459 | 0.012805 | 0.059812 | 0.0078195 |
| 42 | 0.27459 | 0.012991 | 0.11363 | 0.0078399 |
| 468 | 0.097535 | 0.015227 | 0.040438 | 0.0079392 |
| 18 | 0.4643 | 0.01439 | 0.19304 | 0.0080885 |
| 197 | 0.14374 | 0.014667 | 0.059788 | 0.0081109 |
| 19 | 0.44166 | 0.014063 | 0.18394 | 0.0081771 |
| 61 | 0.22757 | 0.012969 | 0.095108 | 0.0083657 |
| 17 | 0.47062 | 0.014175 | 0.19713 | 0.0084899 |
| 46 | 0.26521 | 0.01313 | 0.11113 | 0.0085113 |
| 29 | 0.41157 | 0.016186 | 0.17257 | 0.0085466 |
| 169 | 0.13644 | 0.012905 | 0.057386 | 0.0087181 |
| 110 | 0.15801 | 0.012076 | 0.06675 | 0.0089677 |
| 123 | 0.16677 | 0.013473 | 0.070547 | 0.0090466 |
| 44 | 0.27749 | 0.013437 | 0.11754 | 0.0091228 |
| 1098 | 0.056204 | 0.013206 | 0.023809 | 0.0091267 |
| 13 | 0.49863 | 0.013135 | 0.21128 | 0.0091402 |
| 135 | 0.16894 | 0.014294 | 0.071674 | 0.0092167 |
| 22 | 0.40772 | 0.013969 | 0.17349 | 0.0093896 |
| 161 | 0.14515 | 0.013402 | 0.061769 | 0.0093975 |
| 12 | 0.4707 | 0.011913 | 0.20055 | 0.009468 |
| 12 | 0.56037 | 0.014183 | 0.23888 | 0.0094984 |
| 10 | 0.6595 | 0.015238 | 0.28125 | 0.0095217 |
| 50 | 0.24179 | 0.012479 | 0.10326 | 0.009607 |
| 57 | 0.22611 | 0.012457 | 0.096617 | 0.0096417 |
| 275 | 0.10952 | 0.013176 | 0.046864 | 0.0097281 |
| 32 | 0.32265 | 0.013328 | 0.13826 | 0.0098101 |
| 36 | 0.30135 | 0.013202 | 0.12947 | 0.0099719 |
| 82 | 0.20107 | 0.013278 | 0.086689 | 0.010191 |
| 32 | 0.32119 | 0.013268 | 0.13874 | 0.010311 |
| 39 | 0.27709 | 0.012634 | 0.11995 | 0.01045 |
| 16 | 0.49913 | 0.014585 | 0.21609 | 0.010456 |
| 14 | 0.54334 | 0.014853 | 0.23527 | 0.010467 |
| 99 | 0.18633 | 0.013514 | 0.080711 | 0.01049 |
| 162 | 0.14501 | 0.013431 | 0.062851 | 0.010526 |
| 42 | 0.24223 | 0.01146 | 0.10518 | 0.010644 |
| 39 | 0.29508 | 0.013454 | 0.12829 | 0.010727 |
| 11 | 0.50331 | 0.012197 | 0.2202 | 0.011141 |
| 330 | 0.093589 | 0.012316 | 0.040976 | 0.011192 |
| 63 | 0.23171 | 0.013419 | 0.10163 | 0.011309 |
| 26 | 0.38144 | 0.014205 | 0.16736 | 0.011336 |
| 43 | 0.24705 | 0.011826 | 0.10871 | 0.011533 |
| 19 | 0.3656 | 0.011641 | 0.161 | 0.011586 |
| 1547 | 0.044563 | 0.012269 | 0.019641 | 0.011644 |
| 49 | 0.26976 | 0.013783 | 0.1189 | 0.011649 |
| 141 | 0.14788 | 0.012785 | 0.065344 | 0.011822 |
| 16 | 0.35107 | 0.010259 | 0.15581 | 0.01213 |
| 694 | 0.064572 | 0.0122 | 0.028701 | 0.012238 |
| 122 | 0.1498 | 0.012053 | 0.06679 | 0.012461 |
| 242 | 0.10419 | 0.011769 | 0.046464 | 0.012477 |
| 34 | 0.27882 | 0.011871 | 0.12438 | 0.012499 |
| 127 | 0.15491 | 0.012716 | 0.069117 | 0.012511 |
| 125 | 0.16081 | 0.013096 | 0.072082 | 0.012851 |
| 299 | 0.10383 | 0.013017 | 0.046688 | 0.013082 |
| 316 | 0.090445 | 0.011651 | 0.040682 | 0.013106 |
| 18 | 0.43756 | 0.013561 | 0.19683 | 0.013114 |
| 88 | 0.1672 | 0.011437 | 0.075358 | 0.013258 |
| 60 | 0.19258 | 0.010885 | 0.086825 | 0.013285 |
| 37 | 0.28989 | 0.012875 | 0.13072 | 0.013298 |
| 40 | 0.29313 | 0.013535 | 0.13222 | 0.013319 |
| 18 | 0.41365 | 0.01282 | 0.18659 | 0.013323 |
| 64 | 0.22126 | 0.012915 | 0.099896 | 0.013391 |
| 168 | 0.14074 | 0.013273 | 0.063638 | 0.013502 |
| 6 | 0.7163 | 0.012821 | 0.325 | 0.01377 |
| 91 | 0.18111 | 0.012596 | 0.082287 | 0.013874 |
| 14 | 0.39507 | 0.0108 | 0.17955 | 0.013898 |
| 28 | 0.34005 | 0.013141 | 0.15471 | 0.01398 |
| 58 | 0.21952 | 0.0122 | 0.099918 | 0.014017 |
| 1296 | 0.049341 | 0.012525 | 0.022479 | 0.01409 |
| 100 | 0.16416 | 0.011966 | 0.074813 | 0.014113 |
| 23 | 0.33941 | 0.011889 | 0.15479 | 0.014171 |
| 390 | 0.085088 | 0.012152 | 0.038948 | 0.014464 |
| 19 | 0.37494 | 0.011939 | 0.17177 | 0.014531 |
| 86 | 0.18513 | 0.012518 | 0.084834 | 0.014554 |
| 242 | 0.11988 | 0.013542 | 0.054945 | 0.014566 |
| 116 | 0.16142 | 0.012667 | 0.07409 | 0.014684 |
| 45 | 0.25795 | 0.012632 | 0.11841 | 0.014693 |
| 141 | 0.13654 | 0.011805 | 0.06278 | 0.014828 |
| 14 | 0.40876 | 0.011174 | 0.18805 | 0.014873 |
| 16 | 0.42821 | 0.012513 | 0.19704 | 0.014889 |
| 103 | 0.17052 | 0.012613 | 0.078471 | 0.014897 |
| 50 | 0.22513 | 0.011619 | 0.10363 | 0.014915 |
| 53 | 0.22136 | 0.011761 | 0.10248 | 0.015396 |
| 47 | 0.24093 | 0.012057 | 0.11171 | 0.015516 |
| 126 | 0.14236 | 0.01164 | 0.066089 | 0.015623 |
| 12 | 0.51129 | 0.012941 | 0.23738 | 0.01563 |
| 57 | 0.2353 | 0.012964 | 0.10936 | 0.015723 |
| 154 | 0.14492 | 0.01309 | 0.067405 | 0.015786 |
| 13 | 0.4347 | 0.011451 | 0.2022 | 0.015789 |
| 19 | 0.36191 | 0.011524 | 0.1684 | 0.01582 |
| 15 | 0.493 | 0.013949 | 0.22942 | 0.015826 |
| 27 | 0.32063 | 0.012168 | 0.14955 | 0.016023 |
| 98 | 0.15317 | 0.011053 | 0.071761 | 0.016409 |
| 161 | 0.12225 | 0.011288 | 0.057342 | 0.016511 |
| 6 | 0.69224 | 0.012391 | 0.32543 | 0.016711 |
| 667 | 0.06516 | 0.012078 | 0.030651 | 0.016765 |
| 33 | 0.28435 | 0.011928 | 0.13391 | 0.016867 |
| 128 | 0.14141 | 0.011653 | 0.06668 | 0.016978 |
| 25 | 0.37235 | 0.013598 | 0.17591 | 0.017147 |
| 35 | 0.25444 | 0.010991 | 0.12021 | 0.01715 |
| 36 | 0.30961 | 0.013564 | 0.14674 | 0.017439 |
| 46 | 0.27929 | 0.013827 | 0.13252 | 0.017544 |
| 578 | 0.065039 | 0.01125 | 0.030871 | 0.017576 |
| 13 | 0.4055 | 0.010682 | 0.19253 | 0.017603 |
| 42 | 0.25745 | 0.012181 | 0.12251 | 0.017806 |
| 10 | 0.50343 | 0.011632 | 0.23967 | 0.01785 |
| 14 | 0.42656 | 0.01166 | 0.2034 | 0.017996 |
| 138 | 0.14416 | 0.012331 | 0.068787 | 0.018059 |
| 953 | 0.057378 | 0.012612 | 0.027447 | 0.018294 |
| 42 | 0.24905 | 0.011783 | 0.11923 | 0.018368 |
| 14 | 0.44395 | 0.012136 | 0.21308 | 0.018611 |
| 31 | 0.29224 | 0.011882 | 0.14051 | 0.018779 |
| 686 | 0.060686 | 0.011402 | 0.029208 | 0.018876 |
| 254 | 0.10576 | 0.012235 | 0.050933 | 0.018932 |
| 112 | 0.14998 | 0.011566 | 0.072278 | 0.018998 |
| 253 | 0.10125 | 0.011691 | 0.048807 | 0.019025 |
| 48 | 0.23275 | 0.01177 | 0.11227 | 0.019087 |
| 49 | 0.24648 | 0.012594 | 0.11906 | 0.019222 |
| 59 | 0.20011 | 0.011216 | 0.09674 | 0.0193 |
| 17 | 0.39682 | 0.011952 | 0.1919 | 0.019335 |
| 15 | 0.42851 | 0.012125 | 0.20724 | 0.019342 |
| 18 | 0.41918 | 0.012992 | 0.20273 | 0.019345 |
| 92 | 0.15709 | 0.010985 | 0.076053 | 0.019444 |
| 95 | 0.16737 | 0.011892 | 0.081051 | 0.01947 |
| 157 | 0.12684 | 0.011567 | 0.061571 | 0.019704 |
| 34 | 0.31345 | 0.013346 | 0.15236 | 0.019836 |
| 188 | 0.11674 | 0.011639 | 0.056778 | 0.019898 |
| 221 | 0.11073 | 0.011959 | 0.053964 | 0.020099 |
| 57 | 0.22083 | 0.012167 | 0.10765 | 0.020118 |
| 75 | 0.19477 | 0.012303 | 0.09506 | 0.020241 |
| 81 | 0.19076 | 0.01252 | 0.093134 | 0.020278 |
| 35 | 0.28978 | 0.012518 | 0.14149 | 0.020284 |
| 86 | 0.17102 | 0.011564 | 0.083738 | 0.02057 |
| 25 | 0.2576 | 0.0094071 | 0.12619 | 0.020616 |
| 202 | 0.10494 | 0.010842 | 0.051506 | 0.020813 |
| 20 | 0.36462 | 0.011911 | 0.17943 | 0.021078 |
| 121 | 0.1405 | 0.011259 | 0.069169 | 0.021122 |
| 25 | 0.27726 | 0.010125 | 0.13654 | 0.021155 |
| 16 | 0.38514 | 0.011255 | 0.18969 | 0.021164 |
| 327 | 0.091428 | 0.011977 | 0.045046 | 0.021205 |
| 4 | 0.89198 | 0.013037 | 0.43967 | 0.021248 |
| 11 | 0.52853 | 0.012808 | 0.26069 | 0.021319 |
| 149 | 0.12601 | 0.011197 | 0.062277 | 0.021526 |
| 49 | 0.23447 | 0.01198 | 0.11605 | 0.021675 |
| 39 | 0.26835 | 0.012235 | 0.13299 | 0.021815 |
| 23 | 0.35426 | 0.012409 | 0.17578 | 0.021943 |
| 88 | 0.17611 | 0.012046 | 0.087416 | 0.021979 |
| 410 | 0.073507 | 0.010758 | 0.036516 | 0.022064 |
| 10 | 0.45826 | 0.010588 | 0.2278 | 0.022137 |
| 28 | 0.26069 | 0.010074 | 0.12967 | 0.022196 |
| 28 | 0.28473 | 0.011003 | 0.14171 | 0.022265 |
| 45 | 0.2072 | 0.010146 | 0.10315 | 0.022289 |
| 31 | 0.52283 | 0.021258 | 0.26088 | 0.022535 |
| 817 | 0.058502 | 0.011952 | 0.029205 | 0.022588 |
| 18 | 0.39885 | 0.012362 | 0.19912 | 0.022589 |
| 234 | 0.10461 | 0.011622 | 0.052231 | 0.022604 |
| 41 | 0.24505 | 0.011455 | 0.12262 | 0.022843 |
| 255 | 0.10101 | 0.011709 | 0.05058 | 0.022915 |
| 7 | 0.50271 | 0.0097189 | 0.25193 | 0.023007 |
| 50 | 0.253 | 0.013057 | 0.12689 | 0.02309 |
| 10 | 0.51446 | 0.011887 | 0.25813 | 0.02314 |
| 18 | 0.32872 | 0.010188 | 0.16502 | 0.023191 |
| 14 | 0.48607 | 0.013287 | 0.24421 | 0.023282 |
| 74 | 0.18279 | 0.011469 | 0.091837 | 0.023284 |
| 10 | 0.45186 | 0.010441 | 0.2271 | 0.023319 |
| 82 | 0.19162 | 0.012654 | 0.096326 | 0.023342 |
| 133 | 0.13713 | 0.011517 | 0.069107 | 0.023621 |
| 38 | 0.25239 | 0.011359 | 0.12733 | 0.02374 |
| 15 | 0.41932 | 0.011865 | 0.21222 | 0.024094 |
| 17 | 0.43817 | 0.013198 | 0.22236 | 0.024397 |
| 12 | 0.44501 | 0.011263 | 0.22585 | 0.024407 |
| 91 | 0.17303 | 0.012035 | 0.087864 | 0.024465 |
| 44 | 0.23734 | 0.011493 | 0.12071 | 0.024648 |
| 124 | 0.14061 | 0.011405 | 0.071522 | 0.024662 |
| 30 | 0.2964 | 0.011856 | 0.1508 | 0.024683 |
| 10 | 0.50325 | 0.011628 | 0.25629 | 0.024795 |
| 73 | 0.17768 | 0.011073 | 0.090573 | 0.024907 |
| 68 | 0.18652 | 0.011221 | 0.095172 | 0.025016 |
| 92 | 0.1587 | 0.011098 | 0.081004 | 0.025057 |
| 57 | 0.21576 | 0.011887 | 0.11016 | 0.025091 |
| 118 | 0.14003 | 0.011082 | 0.0715 | 0.025099 |
| 119 | 0.13909 | 0.011054 | 0.071061 | 0.025161 |
| 10 | 0.48861 | 0.01129 | 0.24965 | 0.025174 |
| 525 | 0.069701 | 0.011507 | 0.035669 | 0.025352 |
| 20 | 0.39929 | 0.013044 | 0.20448 | 0.025432 |
| 35 | 0.24128 | 0.010423 | 0.12366 | 0.025526 |
| 31 | 0.27469 | 0.011169 | 0.14091 | 0.025627 |
| 23 | 0.3397 | 0.011899 | 0.17435 | 0.025693 |
| 402 | 0.077364 | 0.011214 | 0.039722 | 0.025738 |
| 174 | 0.11482 | 0.011018 | 0.058971 | 0.025768 |
| 12 | 0.45359 | 0.01148 | 0.23318 | 0.025883 |
| 15 | 0.33873 | 0.0095844 | 0.17425 | 0.02596 |
| 39 | 0.23702 | 0.010807 | 0.12199 | 0.026018 |
| 19 | 0.3549 | 0.011301 | 0.18278 | 0.026095 |
| 14 | 0.39085 | 0.010684 | 0.20156 | 0.026252 |
| 79 | 0.15404 | 0.0099854 | 0.079475 | 0.026306 |
| 30 | 0.29091 | 0.011636 | 0.15009 | 0.026306 |
| 51 | 0.19756 | 0.010297 | 0.1021 | 0.0265 |
| 9 | 0.50664 | 0.011106 | 0.26205 | 0.026605 |
| 6 | 0.56817 | 0.01017 | 0.29391 | 0.026619 |
| 7 | 0.44774 | 0.0086563 | 0.23162 | 0.026623 |
| 63 | 0.19943 | 0.01155 | 0.10329 | 0.026762 |
| 11 | 0.46277 | 0.011214 | 0.23989 | 0.026866 |
| 686 | 0.061838 | 0.011618 | 0.032108 | 0.027066 |
| 50 | 0.20921 | 0.010798 | 0.10867 | 0.027106 |
| 12 | 0.44094 | 0.01116 | 0.22929 | 0.027246 |
| 23 | 0.26122 | 0.0091503 | 0.13612 | 0.027496 |
| 68 | 0.19142 | 0.011516 | 0.099786 | 0.027542 |
| 20 | 0.32853 | 0.010732 | 0.17162 | 0.027804 |
| 47 | 0.21223 | 0.01062 | 0.1109 | 0.027838 |
| 12 | 0.45383 | 0.011486 | 0.2372 | 0.027863 |
| 35 | 0.26265 | 0.011346 | 0.13748 | 0.028051 |
| 46 | 0.2247 | 0.011125 | 0.11772 | 0.028156 |
| 34 | 0.2311 | 0.0098397 | 0.12108 | 0.028164 |
| 258 | 0.086377 | 0.01007 | 0.045285 | 0.028241 |
| 131 | 0.1333 | 0.011112 | 0.069935 | 0.028327 |
| 9 | 0.43401 | 0.0095137 | 0.22778 | 0.028375 |
| 77 | 0.1662 | 0.010637 | 0.087233 | 0.028385 |
| 16 | 0.4019 | 0.011744 | 0.21097 | 0.028395 |
| 29 | 0.30568 | 0.012022 | 0.16047 | 0.028404 |
| 15 | 0.39762 | 0.011251 | 0.20881 | 0.028446 |
| 119 | 0.13454 | 0.010692 | 0.070776 | 0.028668 |
| 8 | 0.58545 | 0.0121 | 0.30867 | 0.028945 |
| 97 | 0.13904 | 0.0099822 | 0.073422 | 0.029142 |
| 17 | 0.34791 | 0.010479 | 0.18376 | 0.029167 |
| 152 | 0.12191 | 0.010941 | 0.064422 | 0.029225 |
| 12 | 0.44352 | 0.011225 | 0.23444 | 0.029263 |
| 12 | 0.42846 | 0.010844 | 0.22657 | 0.029316 |
| 48 | 0.22307 | 0.011281 | 0.11798 | 0.029339 |
| 5 | 0.68074 | 0.011124 | 0.36012 | 0.029366 |
| 159 | 0.11999 | 0.011011 | 0.063557 | 0.029524 |
| 15 | 0.34466 | 0.009752 | 0.1826 | 0.029553 |
| 11 | 0.46941 | 0.011375 | 0.24882 | 0.029618 |
| 70 | 0.17093 | 0.010432 | 0.090628 | 0.029655 |
| 32 | 0.23426 | 0.0096769 | 0.12476 | 0.030224 |
| 797 | 0.050308 | 0.010157 | 0.026794 | 0.030226 |
| 400 | 0.070212 | 0.010153 | 0.037454 | 0.030432 |
| 14 | 0.38919 | 0.010639 | 0.20795 | 0.030644 |
| 83 | 0.15273 | 0.010147 | 0.081669 | 0.030742 |
| 8 | 0.46746 | 0.0096612 | 0.25006 | 0.030792 |
| 426 | 0.067531 | 0.01007 | 0.036137 | 0.030836 |
| 28 | 0.29399 | 0.011361 | 0.15736 | 0.030873 |
| 20 | 0.32639 | 0.010662 | 0.17473 | 0.030893 |
| 12 | 0.49858 | 0.012619 | 0.26706 | 0.030964 |
| 130 | 0.13159 | 0.010928 | 0.070497 | 0.030983 |
| 86 | 0.14545 | 0.0098356 | 0.07794 | 0.031015 |
| 115 | 0.12701 | 0.0099239 | 0.068113 | 0.03112 |
| 1484 | 0.036914 | 0.0099727 | 0.019803 | 0.031167 |
| 60 | 0.1759 | 0.0099422 | 0.094385 | 0.031194 |
| 13 | 0.40554 | 0.010683 | 0.21793 | 0.031387 |
| 80 | 0.14613 | 0.0095321 | 0.078642 | 0.031581 |
| 429 | 0.074395 | 0.011132 | 0.040063 | 0.031666 |
| 5 | 0.54115 | 0.0088425 | 0.29157 | 0.031738 |
| 94 | 0.14824 | 0.010478 | 0.079986 | 0.031929 |
| 375 | 0.072608 | 0.010173 | 0.039187 | 0.031962 |
| 7 | 0.69239 | 0.013386 | 0.37384 | 0.032015 |
| 179 | 0.10527 | 0.010244 | 0.056883 | 0.03212 |
| 52 | 0.19435 | 0.010229 | 0.10505 | 0.032153 |
| 27 | 0.29331 | 0.011131 | 0.15866 | 0.032261 |
| 12 | 0.42052 | 0.010643 | 0.22774 | 0.032418 |
| 5 | 0.83146 | 0.013586 | 0.45092 | 0.032606 |
| 39 | 0.25347 | 0.011557 | 0.13747 | 0.03261 |
| 60 | 0.17384 | 0.0098259 | 0.094365 | 0.032728 |
| 11 | 0.44212 | 0.010714 | 0.24017 | 0.032829 |
| 13 | 0.39796 | 0.010483 | 0.21686 | 0.033253 |
| 26 | 0.26908 | 0.010021 | 0.14675 | 0.033366 |
| 107 | 0.14712 | 0.011091 | 0.080242 | 0.033372 |
| 19 | 0.32221 | 0.010259 | 0.1758 | 0.033424 |
| 29 | 0.24609 | 0.009678 | 0.13442 | 0.033576 |
| 8 | 0.48756 | 0.010077 | 0.26632 | 0.033581 |
| 9 | 0.53765 | 0.011786 | 0.2938 | 0.033633 |
| 276 | 0.083269 | 0.010036 | 0.045592 | 0.033903 |
| 32 | 0.25586 | 0.010569 | 0.14025 | 0.034061 |
| 12 | 0.4292 | 0.010863 | 0.23541 | 0.034143 |
| 15 | 0.39259 | 0.011108 | 0.21534 | 0.034151 |
| 1023 | 0.044891 | 0.010203 | 0.024642 | 0.034257 |
| 50 | 0.19923 | 0.010282 | 0.10942 | 0.034333 |
| 80 | 0.16281 | 0.01062 | 0.089456 | 0.034392 |
| 13 | 0.30355 | 0.0079962 | 0.16691 | 0.034493 |
| 21 | 0.30096 | 0.010074 | 0.1656 | 0.034582 |
| 674 | 0.051154 | 0.0095298 | 0.028159 | 0.034645 |
| 37 | 0.21672 | 0.0096249 | 0.11937 | 0.034738 |
| 384 | 0.075488 | 0.0107 | 0.0416 | 0.034801 |
| 164 | 0.10896 | 0.010153 | 0.060053 | 0.034818 |
| 154 | 0.17938 | 0.016202 | 0.098888 | 0.034851 |
| 35 | 0.2259 | 0.0097583 | 0.12456 | 0.034885 |
| 191 | 0.09911 | 0.0099597 | 0.054706 | 0.035027 |
| 12 | 0.37908 | 0.0095945 | 0.20937 | 0.035111 |
| 15 | 0.3614 | 0.010226 | 0.19996 | 0.035361 |
| 227 | 0.096916 | 0.010607 | 0.05363 | 0.035379 |
| 807 | 0.048686 | 0.009888 | 0.026979 | 0.035577 |
| 158 | 0.10397 | 0.0095108 | 0.057618 | 0.035594 |
| 468 | 0.062913 | 0.009822 | 0.034897 | 0.035716 |
| 13 | 0.45155 | 0.011895 | 0.25049 | 0.035729 |
| 12 | 0.42676 | 0.010801 | 0.2368 | 0.035765 |
| 12 | 0.43582 | 0.01103 | 0.24183 | 0.03577 |
| 31 | 0.25973 | 0.010561 | 0.14431 | 0.035948 |
| 67 | 0.16098 | 0.0096134 | 0.089569 | 0.036151 |
| 18 | 0.33024 | 0.010235 | 0.18386 | 0.036239 |
| 18 | 0.30887 | 0.0095728 | 0.17208 | 0.03634 |
| 88 | 0.15438 | 0.010559 | 0.086025 | 0.03637 |
| 14 | 0.3904 | 0.010672 | 0.21755 | 0.03637 |
| 26 | 0.28841 | 0.01074 | 0.1608 | 0.03645 |
| 14 | 0.32447 | 0.0088699 | 0.18106 | 0.036567 |
| 26 | 0.30495 | 0.011357 | 0.17018 | 0.036581 |
| 19 | 0.33066 | 0.010529 | 0.18457 | 0.03661 |
| 14 | 0.39543 | 0.01081 | 0.22079 | 0.036656 |
| 9 | 0.44034 | 0.0096525 | 0.24594 | 0.036702 |
| 88 | 0.14036 | 0.0096004 | 0.078426 | 0.036761 |
| 61 | 0.17859 | 0.010178 | 0.099936 | 0.036975 |
| 23 | 0.33771 | 0.01183 | 0.18909 | 0.037057 |
| 29 | 0.2692 | 0.010587 | 0.15076 | 0.037087 |
| 14 | 0.31343 | 0.0085679 | 0.17556 | 0.037115 |
| 53 | 0.17733 | 0.0094219 | 0.099347 | 0.037144 |
| 13 | 0.36737 | 0.0096774 | 0.20595 | 0.03724 |
| 56 | 0.19748 | 0.010784 | 0.11071 | 0.037246 |
| 10 | 0.45455 | 0.010503 | 0.25499 | 0.037334 |
| 34 | 0.23447 | 0.0099833 | 0.13162 | 0.037433 |
| 18 | 0.38398 | 0.011901 | 0.21592 | 0.037682 |
| 4 | 0.60955 | 0.008909 | 0.34304 | 0.037802 |
| 21 | 0.30205 | 0.010111 | 0.1701 | 0.037899 |
| 102 | 0.13091 | 0.0096364 | 0.073765 | 0.037985 |
| 50 | 0.19585 | 0.010108 | 0.11044 | 0.038096 |
| 99 | 0.11414 | 0.0082785 | 0.064436 | 0.038255 |
| 25 | 0.30531 | 0.011149 | 0.17238 | 0.03828 |
| 53 | 0.21199 | 0.011264 | 0.11972 | 0.038314 |
| 23 | 0.3194 | 0.011188 | 0.18041 | 0.038335 |
| 10 | 0.42083 | 0.0097235 | 0.23788 | 0.038446 |
| 12 | 0.35079 | 0.0088783 | 0.19847 | 0.038584 |
| 19 | 0.29158 | 0.0092843 | 0.16542 | 0.038984 |
| 23 | 0.29911 | 0.010478 | 0.16985 | 0.039125 |
| 9 | 0.54178 | 0.011876 | 0.30802 | 0.039306 |
| 72 | 0.1507 | 0.0093276 | 0.085687 | 0.039324 |
| 393 | 0.074495 | 0.01068 | 0.042377 | 0.03939 |
| 893 | 0.044152 | 0.0094102 | 0.025137 | 0.039515 |
| 20 | 0.29676 | 0.0096945 | 0.16897 | 0.039532 |
| 9 | 0.48187 | 0.010563 | 0.27446 | 0.039579 |
| 55 | 0.19396 | 0.010498 | 0.11048 | 0.039589 |
| 50 | 0.18974 | 0.0097926 | 0.10811 | 0.039627 |
| 254 | 0.082779 | 0.0095765 | 0.04717 | 0.039644 |
| 28 | 0.2907 | 0.011234 | 0.16589 | 0.039863 |
| 46 | 0.20238 | 0.01002 | 0.11554 | 0.03993 |
| 18 | 0.27504 | 0.0085242 | 0.15706 | 0.039972 |
| 45 | 0.21837 | 0.010693 | 0.12477 | 0.040058 |
| 263 | 0.081604 | 0.009604 | 0.046633 | 0.040075 |
| 140 | 0.10608 | 0.0091392 | 0.060681 | 0.040226 |
| 496 | 0.061313 | 0.0098469 | 0.035106 | 0.040369 |
| 24 | 0.24189 | 0.0086553 | 0.13854 | 0.040409 |
| 12 | 0.40415 | 0.010229 | 0.2316 | 0.040498 |
| 24 | 0.27315 | 0.0097736 | 0.1566 | 0.04057 |
| 383 | 0.070033 | 0.009914 | 0.040152 | 0.040572 |
| 48 | 0.19687 | 0.0099558 | 0.11294 | 0.040662 |
| 12 | 0.4671 | 0.011822 | 0.26802 | 0.040696 |
| 9 | 0.4194 | 0.0091936 | 0.2408 | 0.040787 |
| 194 | 0.099476 | 0.010074 | 0.057218 | 0.041066 |
| 13 | 0.34256 | 0.0090239 | 0.19711 | 0.041122 |
| 199 | 0.097493 | 0.009998 | 0.056116 | 0.041172 |
| 222 | 0.096586 | 0.010455 | 0.055612 | 0.04122 |
| 10 | 0.41353 | 0.0095549 | 0.23856 | 0.041521 |
| 317 | 0.073293 | 0.0094563 | 0.042322 | 0.041664 |
| 39 | 0.2282 | 0.010405 | 0.13197 | 0.041893 |
| 75 | 0.1417 | 0.0089511 | 0.081962 | 0.041924 |
| 256 | 0.080022 | 0.0092935 | 0.046306 | 0.041993 |
| 34 | 0.24018 | 0.010226 | 0.13901 | 0.04202 |
| 1570 | 0.034805 | 0.0096473 | 0.020155 | 0.042108 |
| 12 | 0.32888 | 0.0083238 | 0.19063 | 0.042257 |
| 13 | 0.38226 | 0.01007 | 0.22164 | 0.042302 |
| 7 | 0.46042 | 0.0089014 | 0.26713 | 0.042401 |
| 60 | 0.16205 | 0.0091594 | 0.094056 | 0.042459 |
| 28 | 0.27457 | 0.010611 | 0.15937 | 0.042469 |
| 27 | 0.29182 | 0.011074 | 0.16962 | 0.042681 |
| 18 | 0.36981 | 0.011461 | 0.2151 | 0.042798 |
| 321 | 0.071584 | 0.0092928 | 0.041647 | 0.042834 |
| 16 | 0.28702 | 0.0083874 | 0.16711 | 0.042946 |
| 24 | 0.28182 | 0.010084 | 0.16415 | 0.043012 |
| 26 | 0.2439 | 0.0090831 | 0.1421 | 0.043058 |
| 21 | 0.26652 | 0.0089215 | 0.15531 | 0.043086 |
| 53 | 0.18152 | 0.0096447 | 0.10582 | 0.043149 |
| 31 | 0.23105 | 0.0093942 | 0.1348 | 0.043277 |
| 1240 | 0.03722 | 0.0092565 | 0.02173 | 0.043377 |
| 12 | 0.41124 | 0.010408 | 0.24028 | 0.043504 |
| 72 | 0.19283 | 0.011935 | 0.11273 | 0.043598 |
| 155 | 0.10098 | 0.0091501 | 0.059044 | 0.043621 |
| 18 | 0.37885 | 0.011742 | 0.22156 | 0.043646 |
| 33 | 0.21401 | 0.0089772 | 0.12523 | 0.043739 |
| 8 | 0.39056 | 0.0080718 | 0.2287 | 0.043851 |
| 65 | 0.15465 | 0.0090968 | 0.090681 | 0.044066 |
| 25 | 0.24677 | 0.0090118 | 0.14473 | 0.044102 |
| 22 | 0.27857 | 0.0095439 | 0.16344 | 0.044159 |
| 18 | 0.35326 | 0.010949 | 0.20742 | 0.044285 |
| 22 | 0.28035 | 0.0096048 | 0.16488 | 0.044545 |
| 12 | 0.35406 | 0.0089612 | 0.2084 | 0.044673 |
| 28 | 0.24758 | 0.0095678 | 0.14581 | 0.044768 |
| 11 | 0.42015 | 0.010181 | 0.24761 | 0.044877 |
| 20 | 0.25925 | 0.0084691 | 0.15286 | 0.044948 |
| 10 | 0.42855 | 0.0099021 | 0.2527 | 0.04496 |
| 60 | 0.15867 | 0.0089685 | 0.093764 | 0.045307 |
| 21 | 0.33318 | 0.011153 | 0.19703 | 0.045424 |
| 28 | 0.24692 | 0.0095421 | 0.14618 | 0.04561 |
| 135 | 0.10825 | 0.0091593 | 0.064129 | 0.045712 |
| 24 | 0.28506 | 0.0102 | 0.16897 | 0.045812 |
| 10 | 0.35529 | 0.0082093 | 0.21103 | 0.046135 |
| 20 | 0.2752 | 0.0089903 | 0.16348 | 0.046159 |
| 89 | 0.1486 | 0.010221 | 0.088296 | 0.046201 |
| 72 | 0.15845 | 0.0098076 | 0.094355 | 0.046553 |
| 21 | 0.29392 | 0.0098386 | 0.17507 | 0.046597 |
| 43 | 0.19589 | 0.0093774 | 0.11679 | 0.046756 |
| 248 | 0.080812 | 0.0092393 | 0.048223 | 0.046898 |
| 19 | 0.26526 | 0.0084464 | 0.15863 | 0.047254 |
| 54 | 0.18786 | 0.010075 | 0.11236 | 0.04727 |
| 16 | 0.34522 | 0.010088 | 0.20654 | 0.047325 |
| 14 | 0.34984 | 0.0095632 | 0.20942 | 0.047421 |
| 89 | 0.12943 | 0.0089027 | 0.077516 | 0.047501 |
| 22 | 0.29695 | 0.010174 | 0.17787 | 0.047518 |
| 61 | 0.16591 | 0.0094553 | 0.099428 | 0.047599 |
| 60 | 0.16273 | 0.0091978 | 0.09753 | 0.047616 |
| 23 | 0.2781 | 0.0097417 | 0.1668 | 0.047742 |
| 263 | 0.082766 | 0.0097408 | 0.049643 | 0.047743 |
| 33 | 0.2366 | 0.0099247 | 0.14194 | 0.047774 |
| 250 | 0.07944 | 0.0091186 | 0.047662 | 0.047792 |
| 18 | 0.30645 | 0.0094978 | 0.18391 | 0.047837 |
| 14 | 0.37652 | 0.010293 | 0.22597 | 0.047839 |
| 101 | 0.13789 | 0.010101 | 0.082792 | 0.047919 |
| 12 | 0.34152 | 0.0086438 | 0.20523 | 0.048053 |
| 12 | 0.33801 | 0.0085549 | 0.20318 | 0.048109 |
| 44 | 0.20128 | 0.0097466 | 0.1211 | 0.048261 |
| 18 | 0.36523 | 0.01132 | 0.22031 | 0.048686 |
| 12 | 0.39601 | 0.010023 | 0.23892 | 0.048715 |
| 45 | 0.18675 | 0.0091452 | 0.11268 | 0.048726 |
| 180 | 0.096761 | 0.0094422 | 0.058401 | 0.048786 |
| 37 | 0.21358 | 0.0094856 | 0.12892 | 0.048803 |
| 140 | 0.10768 | 0.0092771 | 0.065036 | 0.048899 |
| 22 | 0.25698 | 0.0088043 | 0.15527 | 0.048959 |
| 27 | 0.27942 | 0.010604 | 0.16882 | 0.048959 |
| 9 | 0.42615 | 0.0093415 | 0.25763 | 0.049058 |
| 108 | 0.12842 | 0.0097257 | 0.077679 | 0.049154 |
| 43 | 0.20411 | 0.009771 | 0.12349 | 0.049189 |
| 13 | 0.41948 | 0.01105 | 0.25388 | 0.049245 |
| 10 | 0.40836 | 0.0094355 | 0.24731 | 0.04936 |
| 10 | 0.42976 | 0.0099299 | 0.26071 | 0.049644 |
